# Gene ancestries reveal diverse microbial associations during eukaryogenesis

**DOI:** 10.1038/s41586-026-10639-9

**Published:** 2026-06-10

**Authors:** Moisès Bernabeu, Saioa Manzano-Morales, Marina Marcet-Houben, Toni Gabaldón

**Affiliations:** 1 Barcelona Supercomputing Centre (BSC), Plaça Eusebi Güell, Barcelona, Spain; 2 Institute for Research in Biomedicine (IRB Barcelona), The Barcelona Institute of Science and Technology, Baldiri Reixac, Barcelona, Spain; 3 CIBER de Enfermedades Infecciosas, Instituto de Salud Carlos III, Madrid, Spain; 4 Catalan Institution for Research and Advanced Studies (ICREA), Barcelona, Spain

**Keywords:** Evolutionary genetics, Phylogenetics

## Abstract

The origin of eukaryotes remains a central enigma in biology^1^. Continuing debates agree on the pivotal role of a symbiosis between an alphaproteobacterium and an Asgard archaeon^2,3^. However, the nature, timing and contributions of other potential bacterial partners^4–6^ and the role of interactions with viruses^7–9^ remain contentious. To address these questions, we used advanced phylogenomic approaches and comprehensive datasets spanning the known diversity of cellular life and viruses. Our analysis provided a revised reconstruction of the last eukaryotic common ancestor (LECA) proteome, in which we traced the phylogenetic origin of each protein family. We found compelling evidence for multiple waves of horizontal gene transfer from diverse bacterial donors, with some likely to have preceded mitochondrial endosymbiosis. We inferred plausible traits of the major donors and their functional contributions to the LECA. Our findings support a contribution of horizontal gene transfers to shaping the proteomes of pre-LECA ancestors and suggest a facilitating role of *Nucleocytoviricota* viruses. Taken together, our results suggest that ancient eukaryotes may have originated within complex microbial ecosystems through a succession of diverse associations that left a footprint of horizontally transferred genes.

## Abstract (web summary)

Phylogenomic reconstruction of the proteome of the last eukaryotic common ancestor sheds light on the origin of eukaryotes, indicating an important role of horizontal transfer of genes from diverse bacterial and viral donors.

## Main

The sharp divide in terms of cellular complexity between eukaryotes and prokaryotes has been deemed “the greatest single evolutionary discontinuity to be found in the present-day world”^10^, and the possibility of extensive gene transfer through prokaryotic (endo)symbiosis has long been considered a stepping stone in this process^11^. The current consensus on eukaryogenesis revolves around scenarios that always involve an endosymbiotic relationship with extensive gene transfer between an alphaproteobacterial endosymbiont and a host with an Asgard archaeal ancestry^2,3^. Phylogenomics has shed light on the phylogenetic placement and potential traits of these two established partners^12–16^. However, some proposed eukaryogenesis models involve at least one other partner^5^ or even serial interactions with various non-alphaproteobacterial symbionts acting as gene donors^4,6^. The large dominance of bacterial over archaeal contributions in reconstructed last eukaryotic common ancestor (LECA) proteomes^4,17^ and the observation that only a small fraction of bacterial-derived proteins can be confidently traced back to Alphaproteobacteria may indicate further bacterial contributions^4,18^. However, alternative explanations of such observations include the difficulty of reconstructing ancient phylogenetic relationships and the presence of horizontal gene transfer (HGT) carried by the Asgard archaeal or alphaproteobacterial partners.

Here we addressed the question of whether protein families within the LECA could be traced back to ancestors other than Alphaproteobacteria or Asgard archaea with a level of support similar to those traced to these broadly accepted partners. To alleviate potential artefacts arising from phylogenetic reconstruction, including the effects of unsampled lineages, contamination and recent HGT, we used state-of-the-art methodologies and compiled curated datasets including representative proteomes with the highest possible qualities, from which we purged low-quality sequences, recent paralogues and sparsely distributed proteins. Our results identify at least two major signals of bacterial ancestry different from Alphaproteobacteria and a consistent set of gene acquisitions inferred to be mediated by *Nucleocytoviricota* viruses.

## Reconstruction of the proteome

To leverage the recent explosion of genome data across the tree of life, particularly among eukaryotes, we reinferred the LECA proteome using an automated approach similar to those used in previous studies^4,19–21^. To minimize known methodological issues in homology and phylogenetic inference, we subsampled existing data to obtain a balanced representation across the eukaryotic tree of life (eTOL), while ensuring a tractable size and the highest possible quality (Methods, Supplementary Methods and Supplementary Tables 1 and 2). We also curated the selected proteomes to remove low-quality and low-complexity proteins. Given our focus on deep evolutionary nodes, we kept a single representative of clusters of recent eukaryotic in-paralogues. We replicated this procedure to generate three alternative 100-proteome datasets (eTOLDBA, eTOLDBB and eTOLDBC) that overlapped with respect to about 46% of their proteins (Supplementary Fig. 1) for assessment of the data-dependency of our results. We clustered proteins in these datasets into orthologous groups (OGs) and defined putative descendants of the LECA (LECA-OGs) as OGs that contained at least five different species, at least three of nine eukaryotic supergroups and the two main eukaryotic stems after removal of potential contaminants (Methods and Supplementary Figs. 1 and 2). LECA-OGs were highly consistent across datasets (more than 96%). To further refine these families, we used protein alignment profile similarity searches against a broad database (broadDB) comprising order-level prokaryotic pangenomes reconstructed from more than 65,000 genomes available at GTDB^22^ and sequence representatives of more than 1.3 million clusters of viral sequences^23^ (Methods). This approach ensured maximal coverage of extant diversity while minimizing the impact of database biases and recent HGT. We next reconstructed maximum likelihood phylogenies from the LECA-OG expanded with the closest broadDB hits (Methods). This was used for assessment of the monophyly of the eukaryotic proteins, which were split into different, monophyletic LECA-OGs (mLECA-OGs) if necessary. We repeated the same procedure with this new set of mLECA-OGs by building new alignment profiles and repeating the broadDB search and phylogenetic reconstructions (Methods). This resulted in a final set of mLECA-OGs (with 79% consistency across datasets) and their phylogenies in the context of their closest non-eukaryotic homologues. Analysis of the earliest splits in the mLECA-OG phylogenies indicated that only 3% of the OGs could possibly result from HGT between eukaryotic supergroups (Methods).

Our reconstructed LECA proteomes comprised an average of 12,907 or 7,751 mLECA-OGs (OGs hereafter) depending on whether the relaxed (3 supergroups) or strict (5 supergroups) criterion was used (Supplementary Table 3), consistent with recent estimates (for instance, 10,233 in ref. ^21^). We approximated the functional potential of the LECA by annotating these OGs using a taxonomically delimited set of the KEGG Orthology database (KOs) (Supplementary Methods); then, we inferred a relaxed consensus annotated proteome of 5,317 KOs (consensus proteome hereafter; see the Zenodo repository^24^ for the list of KOs) consistently predicted in two or more datasets or in one dataset with the strict criterion (Methods). Given the limitations of an automated approach (Supplementary Discussion), the purpose of this reconstruction was to approximate gene families and functional categories likely to be present in the ancestral LECA proteome, rather than to provide a detailed picture of LECA traits.

**Fig. 1:**
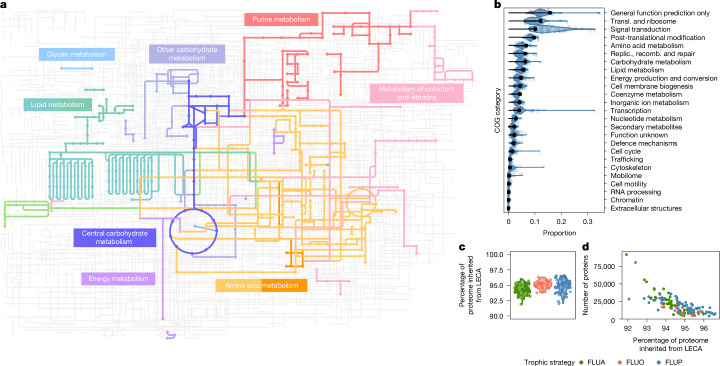
Reconstruction of the ancestral LECA proteome and its metabolic features. **a**, Broad overview of the reconstructed LECA metabolism. Light grey indicates the metabolic map of KEGG; other colours indicate the modules inferred to have been present in the LECA using the consensus proteome, which accounted for the KOs that were present in at least two LECA reconstructions of different eTOLDBs or supported by a LECA group with five eukaryotic supergroups. Each colour refers to a metabolic category. Detailed maps of different modules are available at the Zenodo repository accompanying this article^24^. **b**, Frequency of COG database functional categories in the consensus LECA proteome (black) and in 63 FLUPs, distribution in blue. **c**, Percentages of KOs shared between the ancestral proteome of the LECA and extant FLUAs, FLUOs and FLUPs, respectively. **d**, Relationship between the percentage of the proteome inherited from LECA and the extant genome size. Recomb., recombination; Replic., replication; Transl., translation.

**Extended Data Fig. 1:**
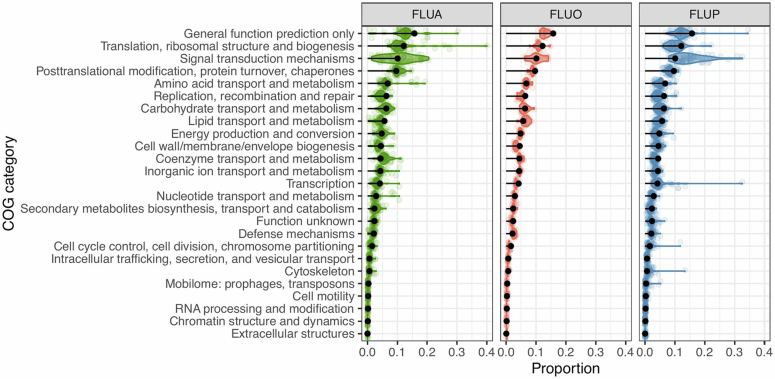
COG categories distribution for free living unicellular eukaryotic genomes with different trophic strategies. Each panel shows a trophic strategy, FLUA: autotrophs, FLUO: osmotrophs and FLUP: phagotrophs. Each point refers to a genome with a given trophic strategy and the black lollipop shows the proportion of that category in the consensus proteome of LECA.

To assess the completeness and validity of our reconstructed LECA proteome, we assessed the reconstructed functional potential in terms of its congruence with previous inferences about the LECA. Our reconstruction confirmed findings of earlier studies depicting a complex LECA, covering the main features of extant eukaryotes^25^ (Fig. 1a,b and Supplementary Discussion). The LECA had the core metabolism and machinery for nucleic acid and protein processing, endocytosis and processing of extracellular particles, and it contained phagosomes, lysosomes and peroxisomes, as well as mitochondria with capacity for aerobic respiration and haem and Fe–S cluster assembly, among other pathways. We inferred a cytoplasm similar to that of extant eukaryotes with a cargo-based transport using motor proteins (dynein, dynactin and kinesin). Conversely, the cell cycle seemed to be incipient, with the presence of basic machinery and very limited regulation (Supplementary Discussion). With respect to metabolism, the low completeness of the autotrophic Wood–Ljungdahl pathway and Arnon–Buchanan cycle indicated a heterotrophic lifestyle. Moreover, the reconstruction lacked enzymes related to anaerobiosis (pyruvate:ferredoxin oxidoreductase (PFO), [FeFe] hydrogenase and enzymes involved in the rhodoquinone synthesis), suggesting that the LECA was most likely to be an aerobe (Supplementary Table 4 and Supplementary Discussion). We compared the reconstructed consensus proteome with the proteomes of representative extant free-living unicellular phagotrophs (FLUPs), osmotrophs (FLUOs) and autotrophs (FLUAs) sampled across the eTOL (Supplementary Table 5). Although FLUPs share the inferred trophic lifestyle of the LECA^2^, we did not observe clear differences in terms of absolute numbers of LECA KOs (Fig. 1c) and COG categories inherited by osmotrophs and phagotrophs (Extended Data Fig. 1 and Supplementary Table 5). Among a wide range of individual variations, specific discrepancies in the relative abundance of functional classes indicated post-LECA adaptations (Extended Data Fig. 1). Notably, all trophic groups had notably expanded repertoires of signal transduction proteins, suggesting post-LECA diversification in ecological and environmental interactions.

## Determining the ancestries of LECA

**Fig. 2:**
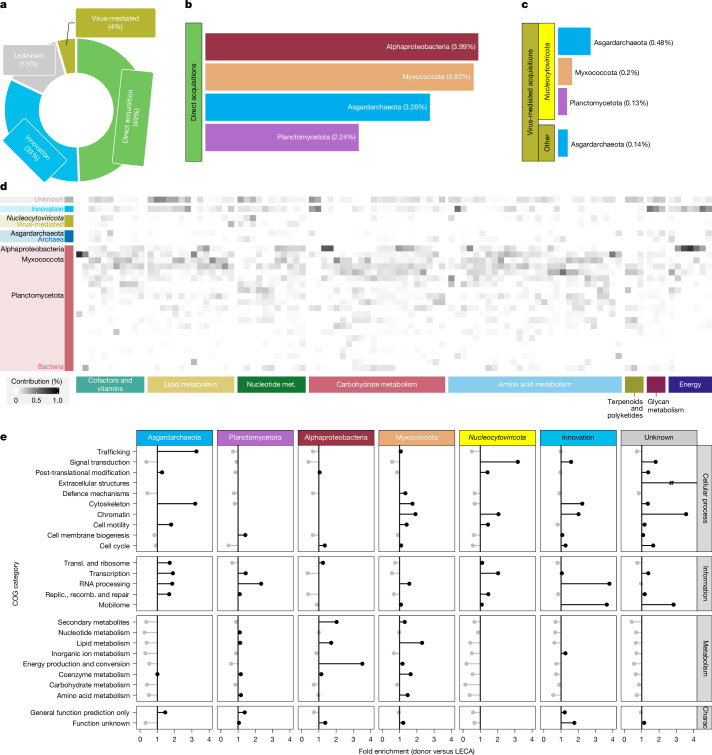
Summary of LECA ancestries. **a**, Circular diagram showing average representation over the LECA proteome across eTOLDBs of innovations, direct- and virus-mediated acquisitions, and families of unknown origin. **b**, Distribution of the ancestries of the LECA proteome. The bar plot shows the main inferred prokaryotic affiliations of genes directly acquired (from prokaryotes to LECA) and their average percentage of representation over the LECA inferred proteomes. **c**, Bar plot as in **b** but for virus-mediated acquisitions (from prokaryotes to viruses to LECA). **d**, Heatmap showing proportions of contributions of each inferred ancestry (rows) to LECA metabolic modules (columns). The intensity of the colour indicates the percentage of annotated KOs from a given metabolic module that are from the taxa ancestry indicated in the rows (for details, see Supplementary Table 4 and the Zenodo repository^24^). **e**, Fold enrichment of functional contributions of the non-eukaryotic genes to the LECA consensus proteome. This was obtained by dividing the proportion of transferred genes with that ancestry in that COG category by the proportion represented by that category in the LECA. Therefore, values greater 1 mean that the gene is more frequent among the donor’s contributions than in the LECA. Charac, poorly characterized; met., metabolism.

We next investigated the topologies of the reconstructed trees for each OG and inferred the phylogenetic sister group as the best proxy for ancestry (Methods and Supplementary Fig. 3). A sizeable fraction of OGs (33%) had only eukaryotic homologues passing our filters; we classified these as putative innovations (Fig. 2a). Such putative innovations may have included some protein families with protein domains or KOs annotations present in prokaryotes, as domain reshuffling or extensive sequence divergence may have resulted in failure of homology detection by our procedures. Considering only KOs in the consensus proteome predicted exclusively as innovations and annotated with eukaryote-exclusive and taxonomically broad KOs, we compiled a list of 918 KOs that could be considered to represent a revised set of eukaryotic signature proteins^26^ (Supplementary Table 6). For OGs with non-eukaryotic homologues (that is, acquisitions; approximately 53% on average; Fig. 2a), we found a diversity of taxonomic assignments, in line with earlier findings^4,17,18,21,27^.

As some affiliations could have resulted from phylogenetic noise, and we were interested in identifying potential relevant partners, we assessed the impact of different stringency thresholds on the proportion of trees assigned to the generally accepted main LECA contributors: Alphaproteobacteria and Asgard archaea (Supplementary Discussion and Supplementary Fig. 4a). Using the thresholds that maximized the proportion of these bona fide affiliations among selected trees, we identified two consistent bacterial affiliations beyond Alphaproteobacteria (3.99% of the LECA proteome, on average) and Asgard archaea (3.28%) that kept a non-negligible proportion of genes: namely Myxococcota (3.92%) and Planctomycetota (2.24%) (Fig. 2b). Further minor bacterial affiliations contributing fewer genes were also identified (Supplementary Fig. 5), including Chloroflexota and Gammaproteobacteria, consistent with previous observations^4^; however, these failed to pass thresholds across all datasets and were therefore not considered in subsequent analysis. The overall representation of the chosen major taxonomic assignments remained highly similar across datasets and stringency criteria, indicating that this result was independent of the eTOLDBs composition (Supplementary Fig. 3). In addition, we found that trees pointing to these alternative taxonomic assignments were not significantly more difficult to resolve than those with alphaproteobacterial sister groups (Supplementary Fig. 6). On the basis of these analyses, we conclude that phylogenetic noise alone cannot result in the observed diversity of taxonomic assignment patterns.

Given the presence of putative other bacterial donors, we considered the possibility that they could result from wrong phylogenetic placement due to incomplete sampling of Alphaproteobacteria. That is, in the absence of a close alphaproteobacterial relative of a given gene in our database, this gene might be assigned to a different bacterial group. We assessed this possibility by applying our OG reconstruction and phylogenetic assignment procedure to alphaproteobacterial genomes, while removing this group from the broadDB (Methods). As expected, this ‘negative control’ showed most of the trees assigned to the closest sister of the missing taxon, Gammaproteobacteria, followed by alternative taxonomic assignments with abundances that decreased as they corresponded to more distant relatives (control bars in Supplementary Fig. 4b). This expected pattern was not observed for our Myxococcota or Planctomycetota affiliations, supporting their representing acquisitions from donors distinct from the mitochondrial ancestor (see different trends in control and LECA bars in Supplementary Fig. 4c and Supplementary Discussion).

Notably, approximately 4.5% of the mLECA-OG phylogenies showed a viral sister group (Fig. 2a), with *Nucleocytoviricota*, a phylum containing giant viruses, being the most frequent viral sister (74%), resulting in 3.32% of the LECA proteome. Trees containing viral sister groups were further analysed in search of the first non-viral sister, resulting in detection of putative virus-mediated acquisitions from diverse prokaryotes (Fig. 2c, Methods and Supplementary Discussion). In addition to innovations and acquisitions (either direct or virus-mediated), a constant fraction of the trees (approximately 13%, on average) had an uncertain taxonomic origin or acquisition directionality, owing to poor representation of the non-eukaryotic sequences in the tree; we labelled these as of ‘unknown origin’. Consistently, signals for all the major prokaryotic affiliations identified here had also been detected to different degrees by previous large-scale studies^4,17,18,21,27^, which used far less complete eukaryotic and prokaryotic genome databases and did not include viruses. Database completeness rather than methodological choices is likely to drive differences across studies, as supported by a reanalysis of the phylogenies of one of these studies with the analytical procedures used here (Supplementary Discussion and Supplementary Fig. 7).

We next assessed the predicted functions of acquisitions assigned to different taxonomic affiliations and found generally broad and patchy distributions across the reconstructed LECA metabolism (Fig. 2d). Conversely, most metabolic modules and cellular processes showed contributions from innovations and acquisitions of different affiliations, suggesting a high degree of chimerism in the LECA (Supplementary Discussion, Supplementary Fig. 8 and Supplementary Table 7). Nevertheless, some functional categories showed overrepresentation of certain origins (Fig. 2e), confirming earlier described trends such as the information/metabolism divide in terms of archaeal and bacterial ancestries^27^ or alphaproteobacterial origins of energy production (Supplementary Discussion).

## Donor relative timing and metabolism

To investigate whether genes showing different ancestries might have been acquired in the form of ‘transfer waves’ (when the inferred timings of different HGTs cluster around a similar period), we applied a previously described branch length ratio approach^4^, as recently implemented into a Bayesian inference framework^28^. This approach involves measuring the length of the branch separating the ancestor of the LECA group from the most recent common ancestor of its non-eukaryotic sister group, normalized using the median LECA-to-tip branch length within the LECA clade, which refers to the same geological time (from LECA to present) and therefore provides a relative time for the acquisition (Methods). In this framework, longer normalized lengths are interpreted as indicating more ancient relatedness.

**Fig. 3:**
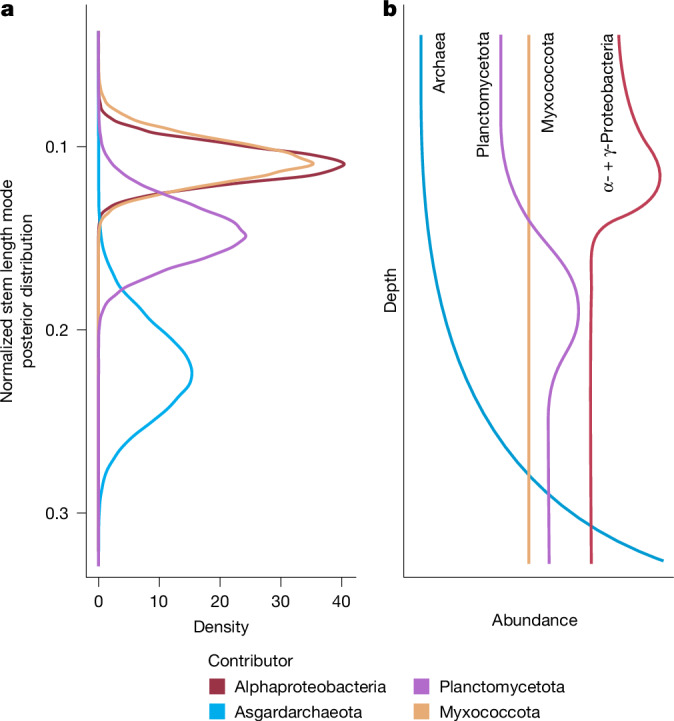
LECA waves of acquisition in the context of microbial mats. **a**, Posterior distribution of the mode (distribution peak) for the normalized stem lengths of each putative donor. Shorter stem lengths refer to more recent transfers, whereas longer ones indicate earlier acquisitions. Colours indicate different donors to the LECA. The posterior distributions for each database and criterion are available in Extended Data Fig. 2, which also includes non-donor signals Gammaproteobacteria and Thermoproteota. **b**, Schematic relative abundances of the inferred donors across the different layers of a microbial mat according to ref. ^29^.

**Extended Data Fig. 2:**
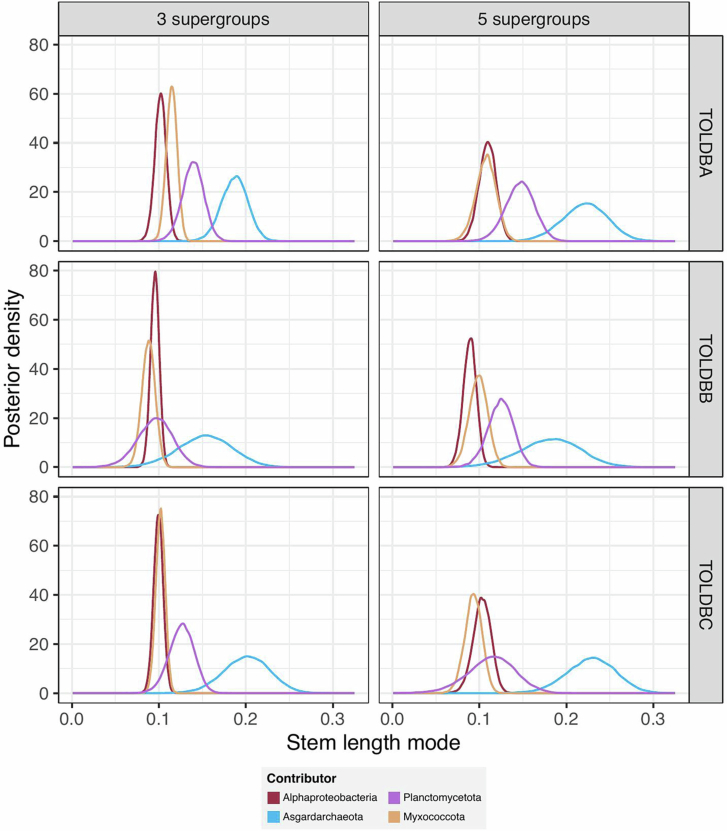
Acquisition waves in LECA. Posterior distribution of the mode of the stem length distribution for each taxonomic affiliation. The panels show the results from different databases (in rows) and selection criteria (in columns).

**Extended Data Fig. 3:**
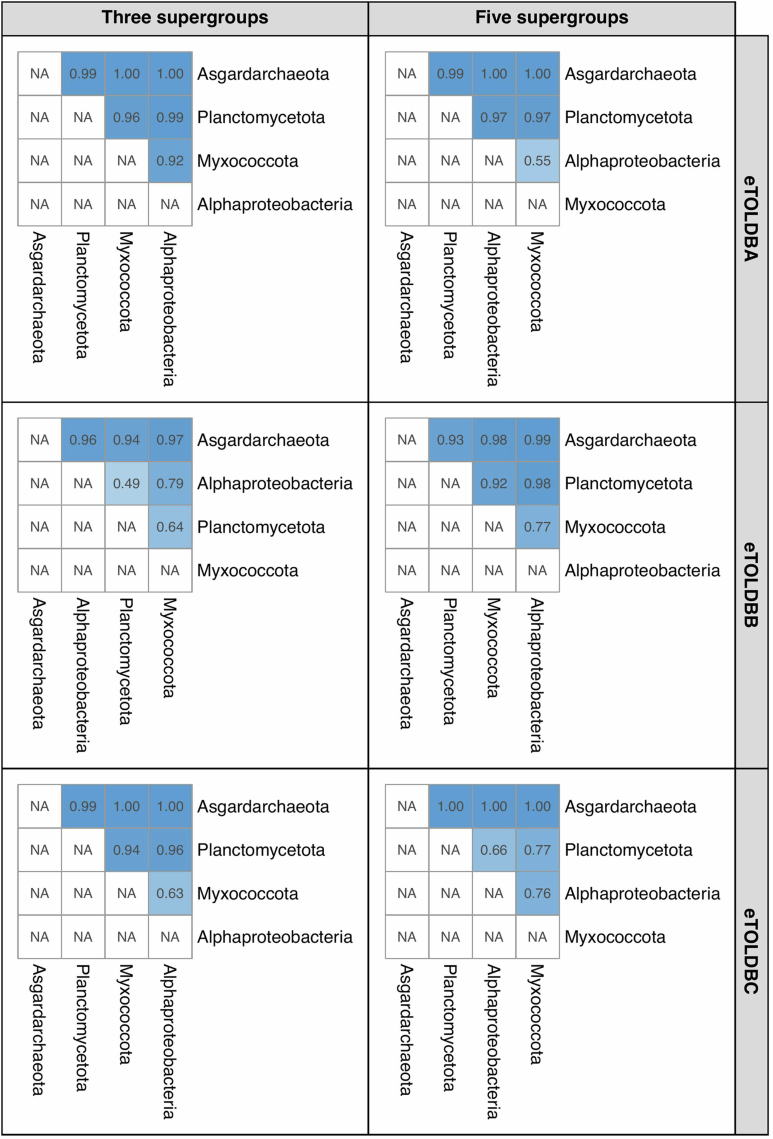
Probability of the waves occurring at different evolutionary relative time points. Each heatmap value shows the pairwise probability of the wave from the donor in the row happening before the wave of the donor in the column. The donors are sorted by the acquisition time from older to more recent.

Our results for the donor lineages (Fig. 3a and Extended Data Fig. 2) depicted distinct branch length distributions peaking at different values, suggesting different relative timings for the acquisitions. With slight variations, we detected consistent trends in the relative ordering of the distributions across datasets and stringency levels (Extended Data Fig. 3). Asgard archaea represented the most ancestral signal. Among the bacterial donors, Planctomycetes tended to show the longest stem lengths, suggesting an early interaction, followed by later contributions from the mitochondrial ancestor and Myxococcota, with largely overlapping transfer waves. Notably, all identified donor bacterial taxa are common components of microbial mats, and the inferred order of acquisition of Asgard archaea, Planctomycetota and Alphaproteobacteria was similar to the relative depth in which each of these taxa has been found in a deeply characterized mat^29^ (Fig. 3b).

**Fig. 4:**
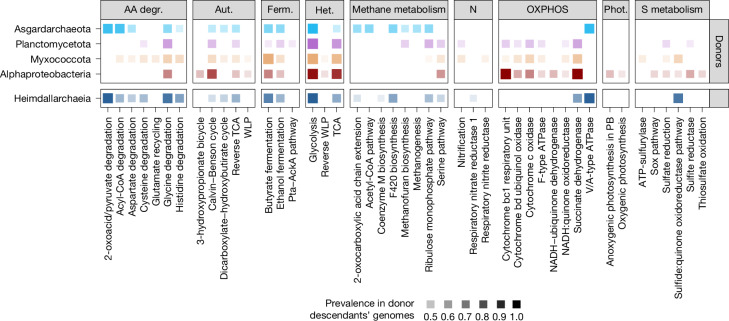
Potential metabolic features of the prokaryotic donors. Prevalence of some metabolic features for each donor. The opacity of a square (with colours congruent with those used in Fig. 2) indicates the proportion of genomes with at least 50% of metabolic pathway completeness. The proposed Asgard sister to eukaryotes (Heimdallarchaeota, at the bottom) is included for comparison. Pathways and KOs with more than 0.4 prevalence are shown. A detailed version of this plot, with prevalence values for all the metabolic pathways and KOs assessed, is available in Extended Data Fig. 4. The equivalent plot considering the origin in the LECA of genes from each donor involved in these metabolic features is available in Extended Data Fig. 5. AA degr., amino acid degradation; Aut., autotrophy; Ferm., fermentation; Het., heterotrophy; N, nitrogen metabolism; OXPHOS, oxidative phosphorylation; PB, purple bacteria; Phot., photosynthesis; S metabolism, sulfur metabolism; TCA, tricarboxylic acid; WLP, Wood–Ljungdahl pathway.

**Extended Data Fig. 4:**
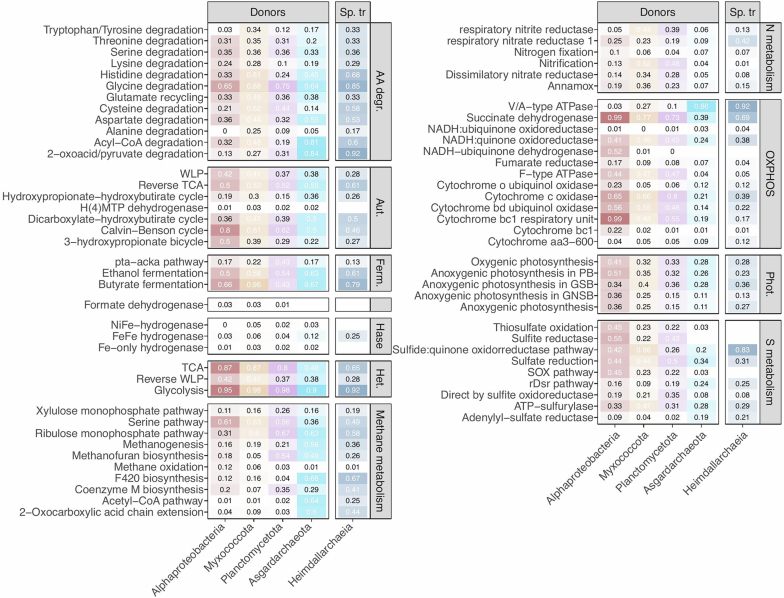
Prevalence of pathways and enzymes in the genomes of the donor’s descendants. Each box shows a group of metabolic pathways and enzymes. The opacity of the colour shows the prevalence (the proportion of genomes with the feature) of that specific pathway or enzyme in extant genomes from the donor’s clades that share at least 50% of the KOs that these donors transferred to LECA. Abbreviations, AA degr.: amino acid degradation; Aut.: autotrophy, Ferm.: fermentation; Hase: hydrogenases; Het.: heterotrophy; N metabolism: nitrogen metabolism; OXPHOS: oxidative phosphorylation; Phot.: photosynthesis; S metabolism: sulphur metabolism.

**Extended Data Fig. 5:**
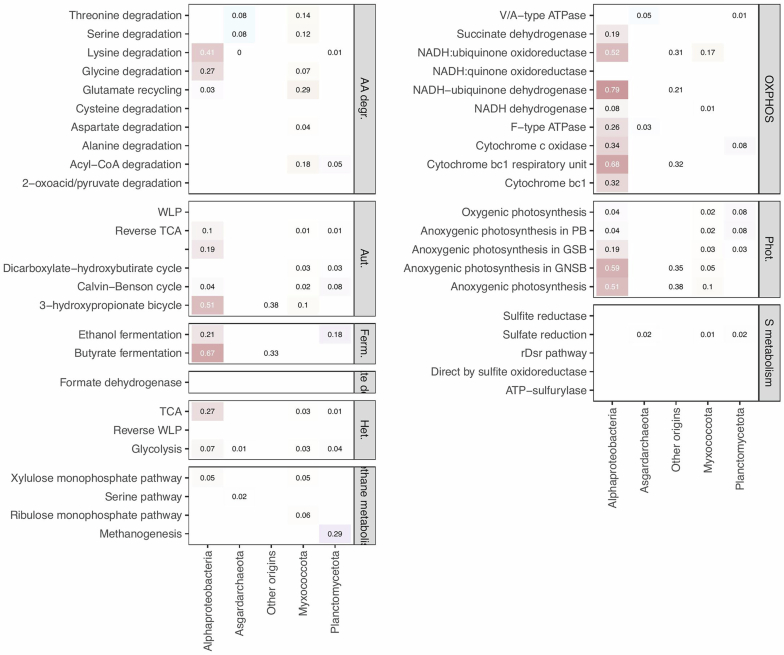
Origins of the pathways and enzymes in the consensus proteome of LECA. Each box shows a group of metabolic pathways and enzymes. The opacity of the colour shows the origin’s proportion. For each KO, we calculated the proportion of origins for all the databases assigned to a clade, and weighted to the total number of KOs in the module, resulting in a proportion for the feature. Abbreviations, AA degr.: amino acid degradation; Aut.: autotrophy, Ferm.: fermentation; Het.: heterotrophy; OXPHOS: oxidative phosphorylation; Phot.: photosynthesis; S metabolism: sulphur metabolism.

We used the above branch length distributions to refine the set of proteins likely to have been acquired from the same donor in a single wave by removing protein families with anomalous branch lengths (Methods). We then inferred the metabolic capacities possibly present in each considered donor by assessing metabolic pathways commonly present and complete among the extant genomes from each donor clade or category that also shared more than 50% of the LECA’s KOs assigned to this ancestry (donor’s descendant genomes; Methods). For comparison, we also considered more restrictive reconstructions by constraining the search to genomes of the assumed ancestor of the archaeal lineage in eukaryotes (Heimdallarchaeia)^12,30^. These reconstructions, which expand KO acquisitions in the LECA with commonly co-occurring KOs in extant organisms of that donor clade, provide an indirect approximation for the core set of metabolic pathways possibly present in the ancestral donors (Fig. 4 and Extended Data Figs. 4 and 5; data available in Zenodo repository^24^). Note that this reconstruction was different from standard ancestral state reconstruction, which necessitates higher resolution on the donor’s taxonomic affiliation and the underlying species phylogeny. Instead, our approach relied on the assumption that KO co-occurrence patterns commonly observed in extant genomes of one phylum were likely to be in place in an unknown ancestor of that phylum, which transferred certain functions to the LECA. The inferred pathways discussed here maintained their overall tendency when we altered the database composition with an iterative resampling approach, penalizing overrepresented taxa (Supplementary Methods).

Considered together, these observations depict a complex scenario involving a series of HGT waves from a diverse set of donors into the protoeukaryotic lineage, as proposed by the premitochondrial symbioses hypothesis^6^. The inferred taxonomic identity, metabolic features and order of acquisition support previous ideas of syntrophic origins within microbial-rich environments such as microbial mats^31^ (Fig. 3b) but also provide further insights. For instance, whereas the hydrogen–sulfur-transfer-based syntrophy (HS syntrophy) hypothesis positioned Deltaproteobacteria (which includes Myxococcota) as the eukaryogenesis host^5^, our analysis suggests that Planctomycetota is an earlier contributor. This, together with the cellular complexity of this group^32^, including the remarkable potential for cell engulfment observed in one genus^33,34^, may lend some support to Planctomycetota as an alternative bacterial host, as previously debated^35–37^. Nevertheless, our data do not provide information on the host or endosymbiotic nature of any partner.

With respect to potential metabolic interactions, although the incomplete and indirect nature of our reconstructions made it difficult to draw specific conclusions, some of our findings were in contrast to other proposed models. For instance, our results indicate that the Asgard ancestor may not have a methanogenic nature, as proposed in various early models^5,31,38^, as none of the Asgard archaeal genomes that resembled the LECA footprint had a complete methyl-coenzyme M reductase (MCR) and coenzyme M methyltransferase (MtbA) (Supplementary Discussion). There was support for fermentative metabolism (with 0.63 prevalence for ethanol fermentation and 0.67 for butyrate fermentation in the archaeal ancestor; Extended Data Fig. 4) as suggested in the reverse-flow model^39^. Our inference moderately supports a potentially photosynthetic mitochondrial ancestor (with prevalence values for the different photosynthetic systems ranging from 0.34 to 0.51), as proposed previously^5,40,41^. Sulfate reduction was inferred, with support for the Myxococcota donor as proposed in the revised HS syntrophy hypothesis^5^ (0.44 prevalence for sulfate reduction) but also for the Planctomycetota donor (0.5 prevalence), suggesting that the model could be adjusted to this alternative host to accommodate our relative timing observations. Both cultured Asgard archaea grow in syntrophy with sulfate-reducing Desulfobacterota partners^42,43^, and sulfur-based interactions have been proposed between extant Asgard archaea and Planctomycetota in mangrove sediments^44^, suggesting that this type of syntrophy can be established between Asgard archaea and diverse bacterial groups. We also observed a weak potential for ammonium oxidizing (anammox) metabolism in the Planctomycetota donor (prevalence of 0.23). Notably, reconstruction of the potential metabolic features present in the mitochondrial ancestor strongly supported the presence of aerobic respiration but not that of Fe–Fe Ni–Fe or Fe-only hydrogenases, as implied by the hydrogen hypothesis and reverse-flow models^31,38,39^. Nevertheless, further phylogenetic, genomic, ecological and biochemical research is needed to elucidate the nature of these early interactions.

## Viruses as mediators of gene acquisition

The contribution of viruses to shaping the LECA proteome has been discussed in previous studies^45–47^. A recent analysis surveying viral sequences across eukaryotes identified a notable amount of virus-to-eukaryote transfer across the eTOL, despite the dominance of eukaryotic-to-virus transfers^8^. Our analysis provides a more systematic and specific assessment of potential virus-mediated HGT into the LECA, which we found to account for at least 4.5% of all acquisitions, after strict filtering to ensure timing and directionality. Most such transfers (74%) were mediated by *Nucleocytoviricota*, also known as nucleocytoplasmic large DNA viruses, a phylum of viruses that are known to infect diverse eukaryotes and include giant viruses with large genomes^48^. We explored trees that showed *Nucleocytoviricota* as the first sister to the LECA and included further prokaryotic sister groups, as their topology suggested indirect acquisition of prokaryotic genes by the LECA through viral mediation. These prokaryotic sisters contained, as well as other prokaryotic groups, all the donor groups detected in the previous analysis, with the most represented donor corresponding to Asgard archaea (approximately 10%; Fig. 2c and Supplementary Table 8). Functional analysis of viral-transferred genes indicated overabundance of genes related to signal transduction mechanisms, mainly kinases, chromatin and post-translational modification proteins (Fig. 2e). This observation was consistent with other findings indicating that viruses of this type infected protoeukaryotes^45^ and indicates the possibility of viral-mediated transfers to the LECA lineage from coexisting but now-extinct eukaryote-like lineages, including genes of prokaryotic origin previously acquired by these lineages^49^.

## Discussion

Our results confirm and expand earlier results supporting sizeable gene flow from diverse prokaryotic ancestors preceding the LECA^4,21^ and uncover a role for viruses as potential mediators of such transfers. Considering the distributions of phylogenetic distances to the closest relatives, our data support premitochondrial waves of gene acquisitions from additional major prokaryotic donors as the most likely scenario^6,50^, against a backdrop of continuous HGT contributions from a diversity of other minor sources. We found support for at least two major non-proteobacterial bacterial donors transferring substantial amounts of genes: Planctomycetota and Myxococcota. Transfers from these donors have been identified in earlier studies^4,18,21^, including small-scale detailed ones such as the acquisition of some steroid biosynthesis enzymes from Myxococcota^51,52^. Our results support the existence of sizable genetic exchange with these alternative donors, suggesting stable interactions beyond those between the Asgard archaeal ancestor and the alphaproteobacterial ancestor of mitochondria. Although different forms of interactions beyond symbiosis could explain this genetic exchange, our results support the expansion of current eukaryogenesis models involving a single interaction between an Asgard archaeal ancestor and the alphaproteobacterial ancestor of mitochondria to cover further interactions with other relevant partners. Models involving interactions with other bacterial partners could be refined in light of the potential donors identified here^5,6^. Our results also uncover a role of viruses, particularly *Nucleocytoviricota*, as mediators of some of these prokaryotic gene transfers. This finding can be considered in the light of previously proposed roles of viruses during eukaryogenesis (Supplementary Discussion).

Our relative timing estimates suggest early interactions between an Asgard archaea and a Planctomycete (bacterium), followed by later contributions of Myxococcota and acquisition of the mitochondrion. Thus, the prokaryotic-to-eukaryotic transition was probably a gradual and complex process, with a constant flow of HGT from diverse sources. Although our data do not provide information on potential types of interaction (endosymbiotic, ectosymbiotic, parasitic, and so on), the inferred metabolic capabilities of the detected donors could be used in comparisons with existing metabolic scenarios for eukaryogenesis to either adjust these scenarios or propose new ones. Microorganisms are known to form complex communities such as microbial mats or complex biofilms^53^, of which viruses also form active part, and it is reasonable to consider that the ancestors of the LECA lived in such complex environments. Successive, perhaps overlapping, long-term interactions with different microorganisms and the mediation of viruses would have led to extensive gene transfer, progressively shaping the chimeric proteome of the LECA.

Taken together, our results challenge models depicting eukaryogenesis as a one-off event resulting from a binary symbiotic interaction and support a paradigm shift towards more gradual scenarios in which the prokaryotic-to-eukaryotic transition was achieved through serial interactions with a diverse range of bacterial partners, leading to substantial gene transfer, with viruses playing a part as mediators.

## Methods

### Data retrieval, curation and proteome database reconstruction

#### eTOLDB construction

To guide dataset selection and LECA reconstruction, we used the phylogenetic backbone of the eTOL and the nine eukaryotic supergroups classification depicted in Supplementary Fig. 1a. This represented a consensus between recent eTOL reconstructions^54–58^ and an operational definition of two stems and nine supergroups that largely reflected previous classifications^59^. Further details are provided in the Supplementary Methods.

We manually selected and retrieved 276 eukaryotic proteomes (Supplementary Table 1; see Zenodo repository^24^ and Supplementary Information) downloaded from NCBI^60^, EukProt^61^, P10K^62^,UniProt^63^, Ensembl^64^ and SGD^65^, aiming to cover all supergroups represented in our reference phylogenetic backbone (Supplementary Fig. 1). We aimed for a balanced representation of divisions and prioritized the most complete and highest-quality genomes for each division on the basis of information available in the hosting databases. For each proteome, we kept only the longest isoform per gene, as indicated in the GFF files or FASTA headers. The isoform-free proteomes were then analysed for completeness using BUSCO v.5.5 (ref. ^66^) with default parameters and eukaryotic_odb10, and we extracted basic statistics using seqkit stats v.2.8.2 (ref. ^67^). To assess for the presence of uncertain amino acids (Xs) and low-complexity regions in the proteomes, we ran SEG^68^ with default parameters. We used an in-house script (‘Code availability’) to trim the low-complexity ends of the proteins and calculated the coverage of low-complexity regions for each protein. If the coverage of low-complexity regions accounted for more than 25% of a protein, we excluded that protein from downstream analyses. Moreover, we calculated the proportion of low-complexity proteins in the whole proteome as a measure of the quality of the protein set and also discarded proteins that were larger than 10,000 amino acids or smaller than 50 amino acids. This resulted in a set of clean proteomes. To remove redundancy and recent duplications (with respect to eukaryotic evolution), we clustered each proteome individually using MMseqs v.2 (ref. ^69^) with strict thresholds: minimum amino acid identity percentage of 80%, minimum target coverage of 0.5 and coverage mode 2 (–min-seq-id 0.8 -c 0.5–cov-mode 2). The representative sequences of the clusters constituted the final proteome. To assess the effects of the cleaning and clustering steps, we again ran BUSCO v.5.5 and seqkit stats v.2.8.2. We discarded proteomes that showed a combined reduction in number of proteins greater than 50%, those with a proportion of low-complexity proteins greater than 50% and those with BUSCO completeness reduction greater than 30%. This resulted in exclusion of 30 proteomes, with 256 proteomes remaining.

We built three eTOL datasets of 100 proteomes each, which we named eTOLDBA, eTOLDBB and eTOLDBC. For this, we first classified the 256 proteomes on the basis of taxonomy and distributed them among 9 different supergroups and 28 divisions (Supplementary Fig. 1). When fewer than four species were available for a division, we included all in the three eTOLDB databases. For the remaining divisions, we selected different, partially overlapping species sets for each eTOLDB. eTOLDBA contained, whenever possible, the most complete proteomes and the fewest parasites. More incomplete proteomes were added in turn to eTOLDBB and in eTOLDBC. Proteomes for model species *Homo sapiens*, *Arabidopsis thaliana* and *Saccharomyces cerevisiae* were included in the three databases despite being part of large taxonomic groups. This was done for later annotation purposes. The list of chosen species for each dataset can be found in Supplementary Table 1. Finally, a total of 185 species were present in the three eTOLDBs with overlaps of 58 species between eTOLDBA and eTOLDBB, and 53 species between eTOLDBA and eTOLDBC and between eTOLDBB and eTOLDBC. This resulted in an average protein overlap of 46% (Supplementary Fig. 1).

#### BroadDB construction

To ensure even sampling of the prokaryotic genome repertoire while minimizing the impact of intradomain HGT as a confounding factor of prokaryotic ancestry, we downsampled available prokaryotic genomes using a pangenome approach. For this, we took all species representatives in GTDB r207 (ref. ^22^) (62,291 bacteria and 3,412 archaea; see Zenodo repository^24^) and selected one genome per genus (sensu GTDB), choosing the one with the maximal CheckM^70^ completeness and, in cases of ties, minimal CheckM contamination. The median CheckM completeness was 94.50%, and the median contamination was 0.990%. The proteomes of each genus representative were then grouped at order level and clustered using mmseqs2 easy-linclust^69^ (–min-seq-id 0.3 -c 0.5–cov-mode 0–cluster-mode 2 -e 0.001). We kept a sequence representative for clusters present in 15% or more of genus representatives in that order, as 15% is the cutoff for the ‘cloud’ component of the pangenome^71^. To this dataset we added representative sequences from 1,319,927 viral protein clusters at 95% identity percentage available at RVDB v.25.0 (ref. ^23^) (see Zenodo repository^24^). For taxonomic assignment of the sequences, we followed the GTDB and RVDB taxonomies.

### Reconstruction of LECA

#### LECA-OG reconstruction

We ran OrthoFinder v.2.5.5 (ref. ^72^), using BLAST v2.15.0+^73^ for the homology search with the filter for low complexity (-seg yes). Before the clustering step, BLAST results were filtered to remove hits with an e-value greater than 1 × 10^−5^ and an overlap less than 0.5. We also removed hits with disparate sizes (when the hit was more than 2.5 longer or less than 0.5 shorter than the query) to avoid artefactual clustering of families due to domain sharing. The clustering part of OrthoFinder was executed using two different inflation parameters (*I* = 1.5 and *I* = 3.0). Comparison between the two datasets showed that *I* = 3.0 gave 30,000 more groups on average than *I* = 1.5. When we filtered to retain only LECA groups, there was an increase of 990 groups on average between *I* = 3.0 and *I* = 1.5. Given the small difference, we chose to continue with the larger groups (*I* = 1.5), as they could be split on the basis of tree topology at a later point.

OGs as defined by OrthoFinder were filtered to select those likely to be present in the LECA (LECA-OG) on the basis of their distribution across the eTOL. LECA-OGs had to comprise proteins from at least five species and to have representatives of the two stems and three or more different supergroups for a relaxed definition of LECA and five or more supergroups for a strict definition of LECA. The same criteria for identification of the LECA were used in downstream analyses.

#### Contamination assessment and filtering of LECA-OG sequences

To assess potential non-eukaryotic contaminant proteins, we used taxonomy-based prok-euk contamination detection. First, we built a broad database to assess contamination. This database contained all prokaryotes from the reference release of GTDB r222 (ref. ^22^) and all eukaryotic proteomes in UniProt^63^, EukProt^61^ and a selection of the P10K^62^ project proteomes. To reduce redundancy and the size of the database, we clustered the proteomes of each class using mmseqs v.2 (ref. ^74^) with the following thresholds: 75% identity and 66% coverage. To flag proteins as potential contaminants, we followed the approach described in ref. ^58^. We performed a diamond blastp v.2.1.9 (ref. ^75^) search against a broad clustered database using an e-value threshold of 1 × 10^−3^, retrieving a maximum of 15 sequences. We considered a protein to be a contaminant if 85% of its first 15 hits were prokaryotic and they showed an identity higher than 85%.

To assess the possibility of eukaryotic contamination, we used two decontamination strategies: taxonomy-based and identity-based euk-euk contamination detection. For the taxonomy-based strategy, we built a database using the eTOLDB sequences and performed an all-versus-all diamond blastp v.2.1.9 search. We retrieved the 15 first hits with identity higher than 85% and considered as contaminants sequences with 85% of their first hits from the opposite stem (following the taxonomic framework in Supplementary Fig. 1). In the identity-based strategy, sequences from different stems with a blast based identity above 95% were considered contaminants, as we could not establish the directionality of the contamination.

Finally, to remove putative contaminants within the same LECA-OG, we performed a multiple sequence alignment of the OG and then calculated pairwise identities between all the sequences of the OG belonging to different stems. Sequences with at least 85% global pairwise identity were considered contaminants. We removed all the sequences considered to be contaminants from the LECA-OGs and reconsidered which LECA-OGs passed the LECA criteria with the remaining sequences.

#### Expanding LECA-OGs with closest non-eukaryotic homologues

We aligned the sequences with MAFFT v.7.525 (ref. ^76^) and built a profile with HMMer’s HMMbuild v.3.3.2 (ref. ^77^). We ran hmmsearch v.3.3.2 with this profile against the broadDB described above. Results were filtered using an e-value threshold of 0.1 for both the complete sequence and the first domain found. Then, to ensure that the coverage requirements were maintained, we performed a BLASTP v.2.15.0+^73^ search for each resulting non-eukaryotic sequence against the database of eukaryotic sequences from the OG. BLAST results were filtered with an e-value threshold of 1 × 10^−5^ and a coverage threshold of 0.5 over the full length of the non-eukaryotic sequence, ensuring that the target sequence was not 2.5 times longer or 2.5 times shorter than the query sequence. The top hits that passed these filters were considered to be the closest non-eukaryotic homologues and were added to the sequences of the corresponding LECA-OG for computation of the phylogenies. The number of non-eukaryotic sequences in the trees was limited in the following way: if there were fewer than 400 eukaryotic sequences, the non-eukaryotic sequences were added until there were 500 sequences. When more than 400 eukaryotic sequences were present, up to 100 non-eukaryotic sequences were added. Eukaryotic groups with more than 500 sequences were processed differently. First, genes were assigned a KO (see below); then, sequences from the same OG with the same KO were grouped together and processed as separate OGs.

#### Phylogenetic tree reconstructions

We used the PhylomeDB tree reconstruction pipeline^78^ with slight modifications. In brief, we computed a consensus alignment using MergeAlign v.3 (ref. ^79^) from alignments obtained using three different alignment programs (MUSCLE v.5.2 (ref. ^80^), MAFFT v.7.525 (ref. ^76^) and Kalign v.3.4.0 (ref. ^81^)) in forward and reverse orientations. This alignment was then trimmed using trimAl v.1.4.rev15 (ref. ^82^) with the -gappyout option. Finally, a maximum likelihood tree was reconstructed using IQ-TREE v.3.0.1 (ref. ^83^) and parameters --mset DCmut, JTTDCMut, VT, WAG, LG --madd LG4X, LG4M, LG + C10 + R4, LG + C20 + R4, LG + C30 + R4, LG + C40 + R4 --mrate E, G, R, I, G + I, R + I --mfreq FU, F -B 1000 --bnni --alrt 1000 --boot-trees --wbtl. Among the available substitution models, we selected single matrix models DCmut^84^, JTTDCMut^84^, VT^85^, WAG^86^ and LG^87^ and mixture models LG4X, LG4M^88^, LG + C10 + R4, LG + C20 + R4, LG + C30 + R4 and LG + C40 + R4 (ref. ^89^). We included different rate heterogeneity parameters (lack of heterogeneity (E), gamma distributed rates (G), the FreeRate model (R), and their combinations with the proportion of invariant sites (I)). We further added two different equilibrium frequency parameters, that provided by the model (FU) and empirical frequencies (F). ModelFinder was used to select from the combinations of all the models and parameters. Branch support was assessed with 1,000 ultrafast bootstrap replicates optimized using nearest-neighbour interchange correction (the –bnni option)^90^ and the Shimodaira–Hasegawa approximate likelihood ratio test^91^.

#### mLECA-OGs

For each LECA-OG, we reconstructed a phylogeny following the phylogenetic reconstruction pipeline detailed above. We annotated the tree with the taxonomy of each group and retrieved the largest monophyletic group containing only eukaryotic sequences and fulfilling the relaxed LECA criterion (five species, three supergroups and two stems). This was considered to be the ‘starting LECA node’. We rooted the phylogeny in the non-eukaryotic node farthest from the starting LECA node. This rooting method was shown to be the one least likely to have a negative impact on the selection of the LECA group and its sister owing to the lack of a proper outgroup (Supplementary Methods). We next iterated through the sister clades of the starting LECA node and incorporated them into the LECA clade if:

1. the sister was mostly eukaryotic: that is, the fraction of non-eukaryotic sequences was less than or equal to 0.5, for sister clades with up to 20 sequences, or less than or equal to 0.25 if the sister had more than 20 tips.
2. If the first sister was mainly non-eukaryotic, but the second sister was mostly eukaryotic (according to the criteria above), the first sister was considered to be a transfer from the eukaryotic clade. In this case, we incorporated the first sister if all the following conditions were met:

- the first non-eukaryotic sister had a number of sequences less than 75% of the number of sequences in the LECA clade;
- the number of non-eukaryotic phyla was less than 3 (assuming low diversity of the first non-eukaryotic sister); and
- the fraction of non-eukaryotic sequences in the second sister was less than 25%.

Once the LECA clade had been defined using the above criteria, we removed it from the tree and performed the search iteratively until no more starting LECA nodes could be found in the tree.

For each LECA node, we selected only the eukaryotic sequences (mLECA-OG hereafter). Finally, we aligned these eukaryotic filtered sequences to compute a profile-based search of non-eukaryotic homologues using the abovementioned criteria. We reconstructed the tree of the resulting set of eukaryotic and non-eukaryotic sequences using the phylogenetic reconstruction described above and identified LECA nodes in this tree following the described procedure. This resulted in a final set of mLECA-OGs that was a refinement of the previous one. Only this final set was used in subsequent analyses.

mLECA-OGs were scanned for the possibility that they emerged owing to HGT events between eukaryotic supergroups. For this, we located the LECA node in the phylogenetic tree, which by definition was ancestral to sequences from stem 1 and stem 2. We then inspected the two clades defined by the two first children nodes of the LECA node to determine whether either of the two nodes contained species from both stem 1 and stem 2 and thus contained the root of the eukaryotic tree. In 97% of the trees, at least one of the children nodes satisfied this condition, making the LECA node unlikely to be the result of an HGT event between diverging supergroup taxa. Given that 29% of the LECA nodes were duplication nodes according to the species overlap algorithm, we searched for the first speciation node within the LECA node and repeated the analysis. In this case, 96% of the trees had children nodes that mapped to the root of eukaryotes, validating the results.

mLECA-OGs were also analysed to assess the possibility of artificial splitting by OrthoFinder by clustering with MCL mLECA-OGs sharing at least 50% of their sister groups (Supplementary Methods); this resulted in minimal difference in our downstream inferences.

#### Functional annotations of mLECA-OGs

We annotated all the proteins with KofamScan v.1.3.0 (https://github.com/takaram/kofam_scan)^92^ and hmmsearch against the COG database (https://ftp.ncbi.nlm.nih.gov/pub/COG/COG2024/data/). Results from KofamScan were filtered in the following way. First, we selected the hits considered significant by KofamScan. When multiple hits were found, we selected the hit with the lowest e-value and highest hmmsearch score. In addition, for proteins with no hits passing the significance threshold of KofamScan, we selected the hit with lowest e-value and highest hmmsearch score. To benchmark the accuracy of our predictions, we used 36 proteomes in our dataset that had been obtained from UniProt and had mappings to genes in KEGG. This enabled us to establish KOs for a total of 154,248 proteins, independent of KofamScan. For those proteins, we calculated the average correspondence of KO predictions per each species. We obtained 95% correct predictions on average, validating our KO annotation method. We further assigned each protein to a COG family by performing hmmsearch using the COG profiles and selecting the first hit on the basis of bit score. We kept those COGs with a bit score higher than the 90th percentile. Using the NCBI COG^93^ definitions table (https://ftp.ncbi.nlm.nih.gov/pub/COG/COG2024/data/cog-24.def.tab), we assigned a COG category to each protein.

Once all proteins had been functionally annotated, we assigned a KO and a COG for each set of sequences (LECA-OGs, mLECA-OGs or donor group, which in virus-mediated acquisitions would be the first prokaryotic sister) using the same approach used to functionally annotate eggNOG OGs. This approach takes into account the proportions of the KO and COG within the set of sequences and their overrepresentation with respect to the background KO distribution (the whole set of annotated proteins). We chose a single KO and COG or multiple KOs and COGs in cases of ties by minimizing the sum of the KO rank in the proportions list and the overrepresentation list. To annotate the mLECA-OGs, we used the common KOs assigned to the LECA clade and its sister if they agreed, and that of the LECA clade if they disagreed (see Supplementary Methods and Supplementary Fig. 9 for details of the algorithm for assignation of KOs to a mLECA-OG). The mLECA-OGs with no non-eukaryotic homologues (innovations) with KOs found only in non-eukaryotic genomes were left unannotated. This can happen owing to local homology between a non-eukaryotic KO and a eukaryotic protein.

#### Inference of the LECA proteomes

For each eTOLDB version (A, B and C) and each LECA criterion (three supergroups and five supergroups), we obtained the set of mLECA-OGs that fulfilled the criteria and inferred their origins and KO annotations. We used these proteomes to infer the general proportion of acquisitions and innovations, as well as the general metabolism of the LECA. Furthermore, we calculated a consensus proteome with the KO annotations that were pervasive and well supported across the datasets to make inferences about LECA features and metabolism. For this consensus, we considered KOs if they were present in two of the three TOLDB datasets or supported by at least five supergroups in any TOLDB dataset.

#### Inference of LECA metabolism and features

We inferred the presence of metabolic pathways and their completeness for each LECA proteome (one per eTOLDB version and LECA criterion) using the anvi-estimate-metabolism module of the anvi’o v.8 (ref. ^94^) package. Finally, we plotted a reconstructed metabolic map using ggKEGG v.1.2.3 (ref. ^95^) (Supplementary Fig. 10).

For other molecular components that were not necessarily associated with a single KEGG map, such as the complexes that form the flagellum, the spliceosome or the cytoskeleton, we collected the set of genes associated with these components as depicted by KEGG BRITE, complemented them on the basis of the literature when necessary, and then checked the assigned origins of the different genes. We assessed the completeness of these features by extracting the subset of KOs that were general to eukaryotes (to minimize the effects of clade-specific KOs in the inference of completeness) and determined the proportions at which they were present in our reconstruction of the LECA by any database (for the origins) and the consensus proteome (for presence and absence of the protein).

#### Comparison with FLUPs, FLUOs and FLUAs

To benchmark the inferred LECA proteome, we compared it to the core and individual proteomes of selected unicellular, heterotrophic eukaryotic organisms. For this, we selected FLUPs, FLUAs and FLUOs from the eTOLDB proteomes, the P10K project^62^ and specific project repositories, (Supplementary Table 5). After cleaning and performing quality checks on the proteomes (‘eTOLDB construction’), we functionally annotated the proteomes following the same protein annotation pipeline as for the eTOLDB genomes (‘mLECA-OGs’).

On the basis of the KO predictions for each genome of the selected FLUPs, FLUOs and FLUAs, we calculated the percentage of each genome that was shared with the LECA in terms of number of KOs (number of shared KOs as a percentage of the number of KOs predicted in the eukaryotic proteome). We also obtained the frequency with which each COG functional category appeared in each genome in the three datasets as the number of genes that had a COG functional category divided by the number of genes with at least one COG category assigned.

### Tracing mLECA-OG ancestries

#### Identifying the origins of mLECA-OGs

First, we analysed each tree by identifying the type of gene family and assigning it to one of three categories: innovations, acquisitions and unknown. We considered mLECA-OGs without non-eukaryotic homologues to be innovations, whereas those that had the LECA group nested within non-eukaryotic sequences were considered acquisitions. Among the acquisitions, we identified two subcategories: viral-mediated acquisitions, which were those pointing to a viral first sister followed by prokaryotic sisters; and direct acquisitions, which were inferred to have acquired the gene directly from prokaryotes. We established another category for those trees whose directionality was not clear (unknown-origin mLECA-OGs). We classified trees as having an unknown origin when they had only viral homologues, fewer than three non-eukaryotic orders or fewer than five sequences. It is important to note, with respect to these thresholds, that the breadth of prokaryotic sequences was heavily downsampled by the pangenomic approach. The sequences present in the sisters do not represent only themselves but also a cluster of other prokaryotic sequences. Therefore, each LECA clade may have been deeper within the prokaryotic group and nested, although we only detected sister-to relationships.

To detect the non-eukaryotic groups that transferred a given gene family to the protoeukaryote, we analysed the first sister of the mLECA-OG in the case of direct acquisitions and the first non-viral sister for virus-mediated acquisitions. First, we discarded a non-eukaryotic origin for the mLECA-OGs for those trees in which the sister clade contained more than 25% eukaryotic sequences, as although the sister would be mainly non-eukaryotic, the presence of eukaryotic sequences could have been the result of secondary transfers from eukaryotes to a broad set of bacteria; this would hamper identification of a confident sister. For the trees that passed this initial filter, we retrieved the proportion of each non-eukaryotic group present in the sister clade. Finally, to assign a taxon to the sister clade, we selected the most abundant group in the first sister (that is, the one with the maximum proportion). In the case of more than one non-eukaryotic group with maximum proportion in the first sister, we assumed that the clade was mixed and calculated its common ancestor; this could be Bacteria, Archaea, Viruses or a mixture of them.

Trees in which the first sister to the mLECA-OG was assigned to *Nucleocytoviricota* were assessed in more detail to assess the directionality of the transfer or the presence of secondary transfers from other groups. For this, we scanned the trees to search for other, non-viral sequences. For each tree with a sister in *Nucleocytoviricota*, we located the point at which the LECA and its closest sister were located and went back in the tree towards the root to locate the first sister for which the largest signal did not belong to viruses. When such cases were found, the taxonomy of that sister was assessed and considered as the potential source of genes transferred to the LECA through the viral lineage. Trees with only viruses as sisters and no other non-eukaryotic signal in the tree were classified as of unknown origin, as it was difficult to assess whether they were the result of a more recent HGT event from eukaryotes to viruses.

#### ‘Stress test’ for identification of main non-eukaryotic contributors

To assess the congruence of the LECA and its relationship to its sister, we checked the ultrafast bootstrap support (UfBS) values of the branch separating the LECA and its first sister (Supplementary Fig. 11). However, the evolutionary depth of this project required a bootstrap support measure that accounted for broader taxonomic patterns rather than specific sequences moving through different nodes. Therefore, we designed the taxonomic bootstrap, a measure of the support of a given taxonomic affiliation for a certain node of the tree. This version of the bootstrap calculates the proportion of ultrafast bootstrap trees that support the same taxonomic assignment for the sister of the maximum likelihood tree regardless of the congruence of the sequences. In this case, we analysed the taxonomic bootstrap of the clade sister to LECA. To gain further insights about the assigned donor, we checked its proportion in the first sister and its presence in the second sister.

We used these measures to obtain the set of non-eukaryotic non-negligible donors beyond the assumed contributors (Alphaproteobacteria and Asgard archaea). We first established a set of parameters that were important for establishing reliable donors: the UfBS, the taxonomic bootstrap described above, the LECA criteria (3–9 supergroups) and the donor certainty (the proportion of the donor in the first sister). We then scanned the whole set of trees to obtain the thresholds for the parameters that maximized the proportion of trees assigned to Alphaproteobacteria and Asgard archaea, assuming that the selected thresholds properly removed noise and kept only the reliable signals. Then, we checked which other donors kept a substantial number of trees using the same thresholds. Although the maximum Alphaproteobacteria and Asgard archaea proportion was given when using an UfBS threshold lower than 75%, we searched for the next maximum with at least 75% UfBS (Supplementary Fig. 4). The resulting thresholds that maximized the proportion of assignments to Alphaproteobacteria and Asgard archaea were: (i) a UfBS higher than 75%, (ii) a taxonomic bootstrap support higher than 95%, (iii) 7 supergroups criterion for identification of the LECA, and (iv) a donor certainty higher than 95%. This resulted in identification of three contributors beyond Alphaproteobacteria and Asgardarchaeota: two bacterial clades (Planctomycetota and Myxococcota) and a viral phylum (*Nucleocytoviricota*).

#### Verticality test

To assess the possibility that bacterial signals emerged through vertical evolution of alphaproteobacterial donors, we repeated the whole LECA prediction process as described above. First, we collected from the GTDB species representatives a set of 35 representative alphaproteobacterial proteomes. We selected a representative proteome per order (maximum CheckM completeness and, in case of ties, minimal CheckM contamination) and then randomly selected 35 orders out of this set. Then, we filtered the pangenome to exclude all alphaproteobacterial protein sequences. Tree reconstruction and identification of ancestral alphaproteobacterial groups were done as described above. An ancestral group was defined by the presence of five Alphaproteobacteria species, depending on the dataset. Then, we identified the sister groups to those ancestral groups and classified them into the groups derived from the GTDB bacterial tree (for instance, S1 was represented by Gammaproteobacteria + Magnetococcia + Zetaproteobacteria). Then, we counted the number of trees that had a sister in each of the categories (Supplementary Fig. 4b). These values served as the baseline vertical signal for each of the main donor groups and represent the expected proportion of trees associated with secondary donors rather than the main alphaproteobacterial donor (Supplementary Fig. 4c).

#### Inferring the acquisition waves

We defined a LECA gene acquisition wave as a set of genes potentially acquired from the same donor during the same period of time. To identify such acquisition waves, we computed the normalized stem lengths as in ref. ^4^ and plotted the distributions of these branch length values with respect to the taxonomic affiliation obtained according to the above procedure. For duplications occurring during eukaryogenesis and resulting in more than one LECA group, we considered the LECA node with the shortest branch length to the non-eukaryotic sister, as in ref. ^21^. This resulted in distributions with different modes for each taxonomic affiliation (Supplementary Fig. 12), whereas applying this approach to trees for OGs annotated to different functions resulted in overlapping distributions with similar modes (Supplementary Fig. 13). We inferred the acquisition module using the density function; the genes that were around the peak of the distribution were selected as members of the acquisition module. To obtain these genes, we set the threshold to the first minimum after the first maximum or, in its absence, the first inflexion point of the density curve (dashed lines in Supplementary Fig. 12). Genes that transferred before the threshold (that is, those that were in the peak) were included in the module. By contrast, those with longer stem lengths were discarded, as we assumed that they were transferred posteriorly or the values resulted from heterotachy or ghost lineages^28,50^.

To infer the relative timing of the transfers and their timeline, we inferred the mode of the stem length distribution using a Bayesian framework^28^. In brief, this process uses a Markov chain Monte Carlo algorithm (JAGS v.4.3.2) to infer a posterior distribution for the parameters, mode and variance of a gamma distribution, which has been shown to properly fit the empirical distribution of the stem lengths^28^. We used a pairwise comparison of the posterior distributions of the mode from different donors (assumed as relative ages) to establish a probabilistic timeline for the different transfers to the protoeukaryote.

#### Inferring the metabolism and features of the donors

To infer the ancestral features of the organisms that transferred genes to the LECA, for a given donor group, we first selected from its genus representatives the genomes sharing at least 50% of the genes transferred from that group to the LECA in its acquisition wave and annotated the genomes (Supplementary Fig. 14). These genomes constituted the ‘donor-like’ genomes set. Second, we calculated the frequency of each KO present in these genomes. Then, we ran anvi’o v.8 (ref. ^94^) for each donor-like genome. For both the metabolic modules and the KOs, we calculated their prevalence (Supplementary Fig. 15), that is, the proportion of genomes containing a given KO or module. In the case of the metabolic modules, we transformed the prevalence by multiplying it by the mean module completeness in all the donor’s descendant genomes to take into account the completeness of the module. Using this metabolic reconstruction, we plotted the inferred donor’s metabolism using ggKEGG v.1.2.3 (ref. ^95^). We further annotated each protein of the donor-like genome using hmmsearch against the COG database and related the results to the COG categories. Using the annotated COG categories, we calculated the overrepresentations of each COG category proportion for a given donor with respect to the LECA consensus proteome. In this case, overrepresentation against the LECA consensus proteome would mean functional enrichment of the donor’s contribution to the LECA. Using the modules and the KO prevalence, we assessed the presence and absence patterns of features relevant to the different eukaryogenesis models for all the donors. No statistical contrast for the enrichment was used, as the sample sizes were non-homogeneous and therefore did not provide enough statistical power, although we could study the general trends.

#### Inferring eukaryotic signature proteins

To define a new set of eukaryotic signature proteins, as defined in ref. ^26^, we selected the KOs from the consensus proteome that were exclusively found as innovations.

### Reporting summary

Further information on research design is available in the Nature Portfolio Reporting Summary linked to this article.

## Online content

Any methods, additional references, Nature Portfolio reporting summaries, source data, extended data, supplementary information, acknowledgements, peer review information; details of author contributions and competing interests; and statements of data and code availability are available at 10.1038/s41586-026-10639-9.

## Supplementary information

Supplementary Information Supplementary Methods, Discussion, Figs. 1–15 and References.

Reporting Summary

Supplementary Tables Supplementary Tables 1–8.

Peer Review File

## Data Availability

All the data used in this study are publicly available from various sources (NCBI, JGI, UniProt, EukProt, P10K, SGD and public repositories of data such as Figshare). A detailed list of the proteomes used in our analyses is given in Supplementary Table 1. All the alignments, trees, annotations and supporting files required to carry out this research are available at Zenodo (10.5281/zenodo.13897946)^24^. The NCBI COG data were downloaded from https://ftp.ncbi.nlm.nih.gov/pub/COG/COG2024/data/.

## Code availability

The code required to reproduce our analysis is available at GitHub (https://github.com/Gabaldonlab/LECAdonors), and an archived version is available at Zenodo (10.5281/zenodo.13897946)^24^.
