# Supplementary material for: Gene ancestries reveal diverse microbial associations during eukaryogenesis
Source: Nature. 2026 Jun 10;656(8127):399–405. doi: 10.1038/s41586-026-10639-9 (PMC13468126; doi:10.1038/s41586-026-10639-9)
Supplement: Supplementary file 2 [file 41586_2026_10639_MOESM2_ESM.pdf]

nature portfolio

Corresponding author(s): Toni Gabaldón

Last updated by author(s): Apr 28, 2026

Reporting Summary

Nature Portfolio wishes to improve the reproducibility of the work that we publish. This form provides structure for consistency and transparency in reporting. For further information on Nature Portfolio policies, see our Editorial Policies and the Editorial Policy Checklist.

Statistics

For all statistical analyses, confirm that the following items are present in the figure legend, table legend, main text, or Methods section.

n/a	Confirmed	
☐	☒	The exact sample size (*n*) for each experimental group/condition, given as a discrete number and unit of measurement
☒	☐	A statement on whether measurements were taken from distinct samples or whether the same sample was measured repeatedly
☒	☐	The statistical test(s) used AND whether they are one- or two-sided<br>*Only common tests should be described solely by name; describe more complex techniques in the Methods section.*
☒	☐	A description of all covariates tested
☐	☒	A description of any assumptions or corrections, such as tests of normality and adjustment for multiple comparisons
☐	☒	A full description of the statistical parameters including central tendency (e.g. means) or other basic estimates (e.g. regression coefficient) AND variation (e.g. standard deviation) or associated estimates of uncertainty (e.g. confidence intervals)
☒	☐	For null hypothesis testing, the test statistic (e.g. *F*, *t*, *r*) with confidence intervals, effect sizes, degrees of freedom and *P* value noted<br>*Give P values as exact values whenever suitable.*
☐	☒	For Bayesian analysis, information on the choice of priors and Markov chain Monte Carlo settings
☒	☐	For hierarchical and complex designs, identification of the appropriate level for tests and full reporting of outcomes
☒	☐	Estimates of effect sizes (e.g. Cohen's *d*, Pearson's *r*), indicating how they were calculated

Our web collection on statistics for biologists contains articles on many of the points above.

Software and code

Policy information about availability of computer code

Data collection

No specific software was used to collect the data we used.

Data analysis

Scripts for custom analyses have been deposited in: https://github.com/Gabaldonlab/LECAdonors and archived on Zenodo under DOI https://doi.org/10.5281/zenodo.13897946

BUSCO, v5.5, https://busco.ezlab.org/
seqkit stats, v2.8.2, https://bioinf.shenwei.me/seqkit/usage/
SEG, v1, https://ftp.ncbi.nih.gov/pub/seg/seg/
MMseqs, v2, https://github.com/soedinglab/MMseqs2
Orthofinder, v2.5.5, https://github.com/davidemms/OrthoFinder
BLAST, v2.15.0+, https://blast.ncbi.nlm.nih.gov/doc/blast-help/downloadblastdata.html
MAFFT, v7.525, https://mafft.cbrc.jp/alignment/software/
HMMER, v3.3.2, http://hmmer.org/
MergeAlign, v3, http://www.stevekellylab.com/software/mergealign
MUSCLE, v5.2, https://www.drive5.com/muscle/
Kalign, v3.4.0, https://github.com/TimoLassmann/kalign
trimAl, v1.4.rev15, https://vicfero.github.io/trimal/
IQ-TREE, v3.0.1, http://www.iqtree.org/
KOfamScan, v1.3.0, https://github.com/takaram/kofam_scan
Anvi'o, v8, https://anvio.org/
ggKEGG, v1.2.3, https://github.com/noriakis/ggkegg
ETE, v3, http://etetoolkit.org/
pypythia, v1.1.4, https://github.com/tschuelia/PyPythia

R, v4.3.2, https://cran.r-project.org/
rjags, v4.16, https://cran.r-project.org/web/packages/rjags/index.html
jags, v4.3.2, https://mcmc-jags.sourceforge.io/
ggmcmc, v1.5.1.1, https://cran.r-project.org/web/packages/ggmcmc/index.html
cumstats, v1.0, https://cran.r-project.org/web/packages/cumstats/index.html
python, v3.11.8, https://www.python.org/
pandas, v2.2.2, https://pandas.pydata.org/
numpy, v1.26.4, https://numpy.org/
polars, v0.20.31, https://pola.rs/
ggplot2, v3.5.1, https://ggplot2.tidyverse.org/

For manuscripts utilizing custom algorithms or software that are central to the research but not yet described in published literature, software must be made available to editors and reviewers. We strongly encourage code deposition in a community repository (e.g. GitHub). See the Nature Portfolio guidelines for submitting code & software for further information.

Data

Policy information about availability of data

All manuscripts must include a data availability statement. This statement should provide the following information, where applicable:

- Accession codes, unique identifiers, or web links for publicly available datasets
- A description of any restrictions on data availability
- For clinical datasets or third party data, please ensure that the statement adheres to our policy

All the data used in this study is publicly available in different sources (NCBI, JGI, UniProt, EukProt P10K, SGD and public repositories of data, such as FigShare). A detailed list of the proteomes used in our analyses is given in the Supplementary Table 1. All the alignments, trees, annotations and supporting files have been deposited in a Zenodo repository: http://doi.org/10.5281/zenodo.13897946. COG database: https://ftp.ncbi.nlm.nih.gov/pub/COG/COG2024/data/

Research involving human participants, their data, or biological material

Policy information about studies with human participants or human data. See also policy information about sex, gender (identity/presentation), and sexual orientation and race, ethnicity and racism.

Reporting on sex and gender	*N/A*
Reporting on race, ethnicity, or other socially relevant groupings	*N/A*
Population characteristics	*N/A*
Recruitment	*N/A*
Ethics oversight	*Identify the organization(s) that approved the study protocol.*

Note that full information on the approval of the study protocol must also be provided in the manuscript.

Field-specific reporting

Please select the one below that is the best fit for your research. If you are not sure, read the appropriate sections before making your selection.

☐ Life sciences ☐ Behavioural & social sciences ☒ Ecological, evolutionary & environmental sciences

For a reference copy of the document with all sections, see nature.com/documents/nr-reporting-summary-flat.pdf

Ecological, evolutionary & environmental sciences study design

All studies must disclose on these points even when the disclosure is negative.

Study description	Phylogenomic study of the origin of gene families inferred to be present in the last eukaryotic common ancestor.
Research sample	A collection of 186 eukaryotic genomes distributed in three datasets, the representative genome collection of GTDB r207 for prokaryotic genomes and the clustered version of the RVDB v25.0 proteins database for viruses.
Sampling strategy	NA
Data collection	We assessed the quality of most of the eukaryotic genomes available using BUSCO completeness, type of data (genome, transcriptome or SAG) and the proportion of low-complexity proteins. These criteria were used to select the final set of 186 eukaryotic genomes based on maximum quality and eukaryotic group representation.
Timing and spatial scale	NA
Data exclusions	Low quality genomes (low BUSCO completeness, and proportion of low-complexity regions) and sequences with a high proportion of low-complexity regions were removed to avoid phylogenetic misinferences
Reproducibility	All the code used to parse the data and analyse the trees is available in the GitHub repository https://github.com/Gabaldonlab/LECAdonors.
Randomization	NA
Blinding	NA

Did the study involve field work? ☐ Yes ☒ No

Reporting for specific materials, systems and methods

We require information from authors about some types of materials, experimental systems and methods used in many studies. Here, indicate whether each material, system or method listed is relevant to your study. If you are not sure if a list item applies to your research, read the appropriate section before selecting a response.

Materials & experimental systems

n/a	Involved in the study
☒	☐ Antibodies
☒	☐ Eukaryotic cell lines
☒	☐ Palaeontology and archaeology
☒	☐ Animals and other organisms
☒	☐ Clinical data
☒	☐ Dual use research of concern
☒	☐ Plants

Methods

n/a	Involved in the study
☒	☐ ChIP-seq
☒	☐ Flow cytometry
☒	☐ MRI-based neuroimaging

Plants

Seed stocks	*Report on the source of all seed stocks or other plant material used. If applicable, state the seed stock centre and catalogue number. If plant specimens were collected from the field, describe the collection location, date and sampling procedures.*
Novel plant genotypes	*Describe the methods by which all novel plant genotypes were produced. This includes those generated by transgenic approaches, gene editing, chemical/radiation-based mutagenesis and hybridization. For transgenic lines, describe the transformation method, the number of independent lines analyzed and the generation upon which experiments were performed. For gene-edited lines, describe the editor used, the endogenous sequence targeted for editing, the targeting guide RNA sequence (if applicable) and how the editor was applied.*
Authentication	*Describe any authentication procedures for each seed stock used or novel genotype generated. Describe any experiments used to assess the effect of a mutation and, where applicable, how potential secondary effects (e.g. second site T-DNA insertions, mosiacism, off-target gene editing) were examined.*
