# Supplementary material for: Gene ancestries reveal diverse microbial associations during eukaryogenesis
Source: Nature. 2026 Jun 10;656(8127):399–405. doi: 10.1038/s41586-026-10639-9 (PMC13468126; doi:10.1038/s41586-026-10639-9)
Supplement: Supplementary file 3 [file 41586_2026_10639_MOESM4_ESM.pdf]

nature portfolio

Peer Review File

Gene ancestries reveal diverse microbial associations during eukaryogenesis

Corresponding Author: Dr Toni Gabaldon

Any redactions in this file are there to maintain patient confidentiality, the confidentiality of unpublished data, or to remove third-party material.

This file contains all reviewer reports in order by version, followed by all author rebuttals in order by version.

Version 0:

Reviewer comments:

Referee #1

(Remarks to the Author)
The origin of eukaryotes represents an important knowledge gap in the evolution of life on Earth. Current data supports scenarios in which the eukaryotic cell evolved via an endosymbiosis between an archaeal host cell and a bacterial (mitochondrial) endosymbiont. Yet, many genes present in the Last Eukaryotic Common Ancestor (LECA) cannot be traced back to either of these two lineages, indicating that additional donors that contributed to the gene content of LECA. The manuscript by Bernabeu et al aims to trace the origin of these genes, reporting that the genome content of LECA and its ancestors evolved “through a succession of symbiotic interactions”, a conclusion that is similar to previous work by Pittis and Gabaldon, and others, which proposed a that the LECA proteome had a chimaeric phylogenetic origin prior to the acquisition of the mitochondrial endosymbiont. The main novelty in Bernabeu et al involves the claim that a significant proportion of LECA gene content is seemingly of viral origin, as it seems to contrast with observations made recently by Irwin and co-workers (Irwin et al, Nature Microbiol, 2022), who reported dominant eukaryote-to-virus transfer, but relatively limited influx of viral genes during eukaryotic evolution. In addition, Bernabeu et al report specific bacterial contributions from Planctomycetes and Chloroflexi to the LECA gene content, something that was not observed in previous studies.
If the above claims made by Bernabeu et al are correct, this would be relevant for scenario’s eukaryogenesis. Yet, given that these findings seemingly contrast with earlier reports, the authors need to show that their results do not stem from methodological issues (see below). Furthermore, some parts of the manuscript are not particularly strong, and therefore misleading, and should be omitted, such as the inference metabolism of prokaryotic donors, and the discussion about biofilms and its relevance for eukaryogenesis.

Main comments

Contrast with earlier studies.
A main claim made in the paper involves that 10% of LECA genes are of viral origin, of which 6% stemming from Nucleocytoviricota. This finding seems to contrast with a recent study that specifically investigated eukaryote-virus HGT (Irwin et al, 2022), where relatively few transfers were observed from virus to eukaryotes (only several hundreds, but not restricted to genes ancestral to all eukaryotes). Another remarkable finding represents that Planctomycetes and Chloroflexi seemingly have donated relatively large numbers of genes to LECA, considering the relatively low number of inferred acquisitions from Planctomycetota and Chloroflexi in Pittis and Gabaldon (2016) and Vosseberg et al. (2021). The question arises whether this could be a result of taxon sampling (or renaming!), or due to the use of other methods? Given that the authors use a different approach to identify/classify LECA genes of non-eukaryotic origin, it should be ruled out that the obtained findings are the result of methodological issues/choices or definitions (e.g. taxon reclassification).

Metabolisms of prokaryotic donors.
The authors also inferred metabolisms of potential prokaryotic donors, which represents a weaker part of the paper. First, the metabolism is inferred based on pan-proteomes defined at (super)phylum or lower taxonomic level. E.g. recent data support that eukaryotes branched from Heimdallarchaeia (Eme et al, 2023), and mitochondria from a sistergroup of all Alphaproteobacteria (Munoz-Gomez et al, 2022). As such, it seems inappropriate to use phylum-level pan-proteomes to infer metabolic properties of the alleged donor lineages. Alos, it is unlikely that present day proteomes are representative for the

donor lineages that donated genes some 2 billion years ago. This becomes clear when looking at Figure 3d, where e.g. methane metabolism is inferred for the Asgard ancestor, which is not in line with most recent data, which do not support a methanogenic host (note that hypothesis in the citations mentioned in line 311 are not supported by any data).

Definition of transfer waves.
The authors make the assumption that transfers traced back to the same taxonomic group were transferred together. What is this assumption based on? Furthermore, 15 genes seem a rather low number to regard transfers as a wave. How many transfers are in the "waves" for the 8 groups considered? The method used is only available as preprint (Bernabeu et al, bioRxiv 2024.09.30.615760) and seemingly obscures the distribution of stem lengths across the individual gene trees. The authors need to also show these.

Handling gene duplications.
It is unclear how the authors dealt with gene duplications in their analysis. Numerous gene duplications occurred during eukaryogenesis. If not in same OrthoFinder OG, would these duplications be in separate trees with largely the same non-eukaryotic sequences, potentially inflating the number of acquisitions? If in the same OrthoFinder OG, the "LECA" node in the tree would actually be a duplication node, influencing the interpretation of branch lengths.

Minor comments
General: "prokaryotic" used for prokaryotic and viral genomes/sequences; change to "non-eukaryotic" or "prokaryotic and viral".

Line 40-41. Please note that the taxonomic (re-)classification of Asgard archaea is currently pending, as another proposal for the nomenclature has been published (Tamarit et al, Syst. & Applied Microbiology, 2024). I suggest you cite both these papers, or neither of the two.

Line 60. "significant": because this is not based on a statistical test, use a different adjective.

Line 98. This sentence is unclear; why mention KOs here where previous sentence mentions mLECA-OGs? What is the connection?

Line 146-148. How does the number of genes without prokaryotic or viral homologs compare with previous estimates?

Line 214-215: "loss or incomplete sampling"; could also be due to phylogenetic artefacts (e.g., LBA).

Line 246: "depending on the dataset", explain or refer to the corresponding Supplementary Figure.

Line 246-249. "Notably, all identified donor bacterial taxa are common components of microbial mats, and the inferred order of acquisition of Asgard archaea, Planctomycetota and Proteobacteria is reminiscent of the relative depth in which each of these taxa is found in this type of environments". This statement is rather ad hoc and cannot be generalized: the composition of a single present-day biofilm is not representative of all microbial mats. Furthermore, what evidence exists for the eukaryotic cell having evolved in a biofilm? Why would organisms present in the same biofilm exchange genes

Line 304-306: one example of phagocytosis-like cell engulfment does not make it likely that the inferred Planctomycetota donor was phagocytotic.

Line 311: methanogenesis not particularly strong in figure 3d, and not consistent with Asgard metabolism. Why is WL not inferred in Asgard archaea?

Line 592-594: eTOLDBA contains the most complete proteomes but how were the other sets selected?

Line 613: "(Supplementary figure cutoffs)"?

Line 625-627: "Little difference was found (...)"; mention this difference.

Line 657-658: does this mean that only one non-eukaryotic sequence was added in cases of 499 eukaryotic sequences?

Line 669-672: two different sets of substitution models mentioned for the same analysis.

Line 724: "Using the definitions table"; which table?

Line 828-829: "set of representative 35 Asgard archaea and 35 Alphaproteobacterial proteomes"; how were these selected? This paragraph describes the analysis presented in figure 2c, but this explanation is also not sufficiently clear.

Figures:

Figure 2:
A: Bars are not proportional to the numbers they represent: not only innovations but also the viruses bar is considerably

shorter than archaea (both 10%). Remove innovations from the figure, so the figure is no longer misleading.
B: Three different values are varied for the stringency measure but it is unclear how this relates to all these different categories on the x axis and what determines their order.
C: Unclear what this panel shows (based on figure legend and main text)

Figure 3:
A: Thermoproteota are not shown.
B: “Archaea” is mentioned where presumably “Asgard archaea” is meant?
C: Rename Mitoancestor and Arcancestor to resp. Alphaproteobacteria and Asgard archaea to be consistent with other figures and the main text and to remain close to the actual results instead of the interpretation; limits x axis not sufficient to show the value of chromatin genes from Nucleocytoviricota.

(Remarks on code availability)
N.A.

Referee #2

(Remarks to the Author)
The nature of the last eukaryotic common ancestor (LECA) and the set of events that gave rise to it (eukaryogenesis) are topics of fundamental biological importance. The past decades have seen vigorous debate over these topics, centring on competing models for eukaryogenesis; and in a growing appreciation for the widespread nature of microbial symbioses has led to symbiogenetic eukaryogenesis models rising to prominence. These models invoke microbial partners other than the alphaproteobacteria-related endosymbiont that gave rise to mitochondria or the Asgard archaeon-related host. Until now, the lack of clear signal for a prokaryotic group other than alphaproteobacteria in the ancestries of eukaryotic proteins has presented a major weakness for these models.

Here, Bernabeu, Gabaldón and colleagues seek to provide an updated reconstruction of the proteome of the LECA, incorporating a wealth of recent datasets that provide broader taxon sampling than was previously possible. They infer the ancestral proteome of the LECA from three different datasets, incorporating two different degrees of stringency. By reconstructing the phylogenies of these proteins, they pinpoint their likely ancestries, and use the branch lengths to draw inferences about the patterns of frequency of their acquisitions. Finally, they reconstruct the metabolism of potential contributors to the eukaryotic lineage.

The work is timely and of broad interest, and the manuscript well written; the authors have gone to a lot of trouble to ensure correct ortholog identification and identify patterns of ancestry in their data. However, I have concerns about the methods that should be addressed in order to fully evaluate the manuscript’s claims, and which may affect its conclusions. The paper also presents a great deal of data derived from functional annotations using automated tools, without taking into account that the databases that these tools rely on are heavily biased toward non-model organisms, and accordingly without carrying out the degree of careful interpretation and manual curation that this approach requires. As a result, conclusions regarding the LECA proteome and new Eukaryotic Signature Proteins need significant revision and clarification.

Major points

Phylogenetic methods
* I’m a little confused by the following passage (starting line 688): “Finally, a maximum likelihood (ML) tree was reconstructed using IQ-TREE v2.3.4 and parameters -wbtl -B 1000 -m TEST -mset WAG,LG,JTT --madd LG4X,LG4M --mrate ALL. We used ModelFinder to select the fittest substitution model among a subset of the available models in IQ-TREE. We selected the single matrix substitution models DCmut, JTTDCMut, VT, WAG and LG and the mixture models, LG4X and LG4M”. In the second line, three models are mentioned that are not included in the command shown in the first line.

* Appropriately, the authors do not simply use ModelFinder with default parameters, but include LG4M and LG4X among the models evaluated. However, it seems clear that they do not include any of the C-series (C20, C40 etc.) profile mixture models, even though these incorporate mixtures of at least 20 amino acid site frequency profiles that are better able to model site heterogeneity (see Baños et al. 2024 https://doi.org/10.1093/sysbio/syad063). This is particularly important given the nature of these datasets, covering large time divergences and possibly functional divergence between lineages.

* If they did use the command shown in line 688, they sought to include all rate heterogeneity parameters in the model evaluation by using the --mrate all option. However, the standard model selection, which they used (-m TEST) does not evaluate FreeRate models (e.g. R3-R10. LG4M and LG4X are also FreeRate models, but these were added explicitly, although note that these are not tested together with other parameters such as +I or +F unless listed in the -mset option rather than with -madd). -m TESTNEW would be the appropriate option to allow these to be evaluated as well. See http://www.iqtree.org/doc/Command-Reference for more details. I don’t have access to the authors’ own raw IQ-TREE output files, but by running some commands myself, I find that the best model recovered when I use -m TESTNEW in most cases includes one of the FreeRate parameters, which is not the case when I only use -m TEST.

* The authors evaluated support for the sister relationship between eukaryotes and their closest sister group using ultrafast bootstrap support values. As far as I can tell, however, they did not optimize UFBoot trees by nearest neighbor interchange (-

bnni option), which reduces the risk of overestimating branch supports due to severe model violations (which is likely to occur in at least a subset of trees here due to compositional heterogeneity, heterotachy or both), even when mixture models are used as recommended above, so this option should be used

* Importantly, to evaluate potential donors to the LECA lineage, the authors used an Ultrafast bootstrap support value cut-off of greater than or equal to 75% in their stress tests. According to the developers of the Ultrafast bootstrap method, this is not appropriate, as support values of 95% correspond to a p-value of 0.05 (see Minh et al. 2013 https://doi.org/10.1093/molbev/mst024, http://www.iqtree.org/doc/Frequently-Asked-Questions). Accordingly, 95% ultrafast bootstrap support should be used as a cut-off value, potentially affecting the major conclusions of the paper.

I realise that some of the changes outlined above are more computationally and time-intensive than the ones that the authors used, but I don't consider them excessive given their importance to the paper's fundamental conclusions.

Other methods/interpretations
* The authors examined the distribution of branch lengths in single-gene phylogenies as a proxy for the timing of HGT, in order to identify waves of such events from a single donor that might correspond to a tighter/more prolonged association: "To identify such acquisition waves, we computed the normalised stem lengths as in 3 and plotted the distributions of these branch length values with respect to the potential prokaryotic donor. [...] We inferred the acquisition module using the density function, the genes that are around the peak of the distribution are selected as members of the acquisition module. To obtain these genes, we set the threshold to the first minimum after the first maximum or, in its absence, the first inflexion point of the density curve. Those genes that transferred before the threshold (are in the peak) are included in the module. In contrast, the ones having longer stem lengths are discarded, assuming that they were transferred posteriorly or the values result from heterotachy or ghost lineages".
This method would seem to inevitably lead to the production of a normal distribution of events over time, which makes using it to try to identify such distributions a circular approach. What do the distributions look like when events with longer stem lineages (described as 'anomalous' in the main text) are not discarded? Is a major peak still apparent in each case?

* I'd also like to see what the distribution looks like for an example or two of less well represented donor groups, for comparison.

* Fig. 2b depicts the number of trees in which eukaryotes are sister to Asgards and Alphaproteobacteria, respectively. However, given that one of the messages of Fig. 2a is that several other groups are presented to an approximately equal degree in the inferred ancestries of LECA proteins, it would be informative to include these lineages in Fig. 2b as well.

* 795, "In the case of more than one eukaryotic group with the maximum proportion in the first sister, we [...] calculated its common ancestor" - how was this done? Did the authors consider the branching order of members of different clades in the sister group (i.e., members of a group branching first are more likely to be the true sister group, with the other group represented having subsequently acquired the gene from the same source)

* It's difficult to tell what exactly will be included in the supplementary data files since these are not currently available, but these should include the list of datasets originally considered (not just those retained for final analyses), and the complete list of representative prokaryotic datasets used, and these should be referenced in the text.

Interpretation of functional annotations

* The paper presents the LECA proteome as one of its major conclusions, but it doesn't seem to me that Figure 1 conveys the most important or surprising findings of this effort for the reader. Though this is not the authors' fault, the overall pathway figure can't show enough detail to be useful, and the larger categories shown in it are so broad ("Lipid metabolism", "metabolism of cofactors and vitamins") as to not be informative in the context of a LECA proteome reconstruction either. "Defense mechanisms" in Fig. 1b is a category that would be interesting to comment on in more detail, but the paper doesn't do this. I'd suggest replacing this figure with one summarizing the key features of the LECA (similar to the general idea of Supplementary Table 9).

* More seriously, if a LECA proteome is being presented, then the functional work needs more specialized curation/interpretation by collaborators familiar with the specific pathways/cellular functions under discussion (this is too much work to realistically expect the small number of authors here to do well). If taken seriously, this would be enough material for a stand-alone paper, beyond the messages focusing on donor lineages of this one, though it would need to be taken with a large grain of salt, and the annotation effort would need to take into account issues with the LECA frequently only having had one ortholog of multiple named orthologs in databases. It would also need to take into account issues annotating LECA proteins with only a subset of the domains found in orthologs with functional data, thanks to domain gain/shuffling in LECA descendents.
Below, I'm choosing some examples found more or less at random that jumped out at me, but I'm sure that someone with more background in some of these proteins could find many more examples than I. For instance:

- Supplementary Table 4, 'Inference of the KEGG modules inferred using the KOs of the consensus proteome of LECA', isn't informative as such, without an extra degree of curation. As it stands, it makes sense that rows highlighted in red are considered by the authors not to have likely been present in the LECA, but what about rows not highlighted in red? Is the reader to infer that the LECA may have had photorespiration? What about heparan sulfate degradation (heparan sulfate being a basement membrane component produced by animals and so unlikely to have been around the LECA much)? It

could be argued that the figure just represents pathways that could hypothetically be reconstructed from the proteins present in the LECA, rather than inferences about their actual function, but that seems pointless (and misleading to any readers not familiar with these pathways). So I would strongly suggest removing it as it stands. Supplementary Table 10 summarises pathway components arising in different ancestors with a similar degree of unquestioning model animal-centric annotations, and I feel that it too should be removed as being uninformative.

- Meanwhile, Supplementary Table 9 summarises the findings, but in such a general way as not to be particularly useful either, as it's difficult to even know when the functional interpretations are likely to be correct. The only descriptor given for 'transcription factors' is "Almost all the complexes are complete." What on earth does this mean? Caspases, including initiator are also extremely unlikely to be present in the LECA – this is most likely misannotation of caspase-related cysteine proteases (metacaspases, paracaspases, orthocaspases) with different cleavage specificities and functions.

- Supplementary Table 8 lists several proteins annotated with the names of bacterial proteins among the Eukaryotic Signature Proteins, including hyfF, sprT, fliT, rub, archaeal flagellar protein flaD, cpeA (phycoerythrin alpha chain, a phycobilisome protein…) and so on. Something has clearly gone wrong here with either the ESP identification or the functional annotation. The list also includes animal-specific proteins (such as NF-kappa-B-repressing factor) that happen to have domains that are more broadly found across eukaryotes, which likely explains the misannotation of these proteins.

* The abbreviations used in Fig. 3 (and Extended Data Fig. 3) should be fully explained in the figure legend(s).

* The authors haven't actually shown that the interactions that gave rise to the patterns they observe were symbiotic (though they favour this explanation, and present plausible possible scenarios for it). Repeated exchange of plasmids between members found abundantly over different time periods within the same type of environment would be equally plausible. "Diverse ancestries reveal complex interactions during eukaryogenesis" would be more accurate.

Minor points

It would be really helpful in the future to number the lines in the supplementary methods/discussion as well as in the main text

Figure 2b shows shows the relative contributions of Alphaproteobacteria and Asgardarchaeota through different stringency parameters, i.e. different Ultrafast bootstrap support values for the branc connecting the LECA node with its prokaryotic sister group. These values should be shown on the axis for ease of reading (similarly, Supplementary Figure 6).

Figure 3b depicts alpha+gammaproteobacteria with a single colour, the same one used to depict only alphaproteobacteria in Figure 3a; it would be helpful to differentiate between these colours (if there is no way to differentiate between the two proteobacterial phyla at different depths; or to clarify in the legend if both alpha- and gammaproteobacteria independently display the same relative abundance curves over depth)

Figure 4 shows a violin plot, but no statistical analyses that would allow the authors to say 'significantly' here. Visually the Alphaproteobacteria and Nucleocytoviricota look as though they could be significantly different, but without statistical tests this can't be confirmed.

39, "a genome merger" - I understand where the authors are coming from, but I'm not a fan of this term as EGT isn't fundamentally different from any other kind of repeated LGT from a single donor lineage. I'd replace this with "a symbiotic relationship with extensive gene transfer"

59, "which we purged from low-quality sequences" should read "from which we purged low-quality sequences"

191-193, "We found no significant differences in terms of the amount of phylogenetic signal among sets of trees with different taxonomic assignments" - the referenced Supplementary
678 (and Supplementary Materials), "monophyletic clade": clades are by definition monophyletic, so this should just be 'clade'

306, 369 and Supplementary Methods (p.13) have a few instances of a typo, 'Plantomycetota' instead of 'Planctomycetota'

681, "farthest to": should be "farthest from"

Supplementary Methods (p.2), "we [...] grouped them into another artificial supergroup that we named TRASH just for labelling purposes in our analysis" - I appreciate this name and hope that it catches on

In the Supplementary Methods (p.2), it seems strange to choose to base the position of the root of eukaryotes in part on Torruella et al. 2024 (bioRxiv) without also mentioning and citing Williamson et al. 2024 https://doi.org/10.1101/2024.09.04.611237. This preprint also supports the split between Stem 1 and Stem 2 that the authors have chosen.

(Remarks on code availability)
The code was not available (Error: 404)

Referee #3

(Remarks to the Author)
This is a very thorough study of the gene/protein donors to the last eukaryotic common ancestor (LECA). Using a combination of previous approaches (stem length analysis like in Pittis and Gabaldon 2016 Nature) and newer contributions (e.g., production of a new LECA proteome prediction), the authors carry out a broad suite of analyses concluding that eukaryogenesis involved “multiple waves of horizontal gene transfer”, some of which may have predated the evolution of proto-mitochondria from alphaproteobacteria. They also posit that Nucleocytoviricota facilitated eukaryogenesis.

The logic behind the dataset assembly, curation and analysis is generally sound, and I commend the authors for their rigor and attention to detail on many levels. I like how the paper discusses the fit of their results with various hypotheses for eukaryogenesis and the potential partners / donors involved. At the same time, however, I think the symbiosis emphasis in the title and abstract is oversold. I would say that their paper indicate that the data are complicated, and on balance, the multiple waves model presented is still consistent with several eukaryogenesis scenarios. So one can question how far this investigation pushes the boundaries of knowledge. There is also the question of the robustness of the stem length / branch length approach taken by Gabaldon and colleagues in the past. -Line 269: “We used these branch length distributions to refine the set of proteins likely acquired from the same donor in a single wave by removing protein families with anomalous branch lengths (see Methods)”. I know that a lot of thought and care has gone into ruling out biases, normalizing the data etc. etc. But I still think that proteins coming from the same donor in a single wave could have very different branch lengths for molecular/cell biological /evolutionary reasons, in which case how can we be sure that proteins with the same branch lengths come from the same wave? I can’t actually tell whether the authors have refined their methodologies in this area in this paper compared to their earlier strong contribution (2016).

I have a number of comments about the figures, which are summarized under specific comments below. Before that I will address what is for me the biggest sticking point with the results as presented and interpreted in this paper, i.e., the inferred contribution of viruses to eukaryogenesis. Everything that we know about viruses in general and large ddDNA viruses in particular tells us that they do not evolve in remotely the same way as cellular life. Despite the original debate about the significance of Mimivirus, and then Pandoravirus, etc. (e.g., exceptionally large genomes and the presence of a handful of ‘cellular genes’ (translation factors etc)), I can’t see any evidence indicating that they have a core genome that could be considered stable enough for an analysis of this sort. There is every reason to assume that the genomes of ddDNA viruses around at the time of eukaryogenesis would have been every bit as dynamic as extant viruses.

A few examples of text to contextualize my point:
-Line 283. “The viral contribution to LECA is enriched in signalling and cell cycle control functions, which are complemented with innovations.”
-Line 342: “Functional analysis of viral-transferred genes indicated over-abundance of genes related to signal transduction mechanisms, mainly kinases, and post-translational modification proteins.”

These enrichments / over-abundances appear to be robust in the author’s analysis, but research also shows that signaling and cell cycle control are functions that giant viruses have acquired from their hosts, where they can play a role in host manipulation. There are many examples of this, including in viruses that today infect eukaryotes but also bacteriophage like the cyanophage that manipulate photosynthesis of their hosts. This is the nature of host-virus interactions today, and I think we can be confident that it was no different in LECA times.

- Line 337 – “We explored trees that showed Nucleocytoviricota as the direct sister to LECA and that were in the same acquisition wave. 226 out of the 505 genes (45%) had a bacterial sister that preceded the divergence between LECA and the viral taxonomic groups, suggesting the DNA viruses may have mediated gene transfer into the LECA lineage.” This is interesting, but what about the other 55%? What were their sister lineages in those cases? Archaeal? A mix of sequences from archaea and proper eukaryotes?

The viral data examined in this paper have passed certain bioinformatic criteria and thresholds for inclusion / interpretation. I’ve looked closely at their methods and Supplemental Discussion, and while the authors touch on the issue of directionality, they don’t directly address it, as Irwin et al. did in 2022, where it was inferred that euk-to-virus transfers are much more abundant than the reverse (this paper is cited in the intro, but not in a specific way).

So I am questioning whether the same criteria used for analysing prokaryotic and eukaryotic proteins are suitable for folding viral homologs into the broader analysis and interpretation. Can we be reasonably sure that the ddDNA viral pangenome core is stable enough to be included in an analysis like this? If it is, it is not demonstrated in this paper. The fact that papers carrying out deep phylogenetic analyses of viruses struggle to find more than a half-dozen genes that are even shared by all the viral lineages of interest – let alone genes that are suitable for phylogenetic analysis – suggests to me that there is no useful core for a deep level, eukaryogenesis analysis such as this.

To be fair, the authors are suitably cautious in some places with respect the role of viruses. For example, “viruses may have mediated gene transfer into the LECA lineage” (above). But in their discussion (line 361) they say: “Our results also underscore a central CONTRIBUTION FROM Nucleocytoviricota, and suggest a potential role of these viruses in mediating some of the gene transfers from the bacterial donors.” In the abstract, they speak of a “facilitating role of Nucleocytoviricota viruses”, and their Supp Discussion starts off: “Our results suggest an important role of viruses during eukaryogenesis.

In the end I’m still left wondering what the role of viruses in eukaryogenesis actually was, beyond gene transfer vehicles

within and between the cellular lineages that were in the mix. Is this very surprising? As long as there have been cells there have been viruses infecting them. From the quotations above, the authors are saying their data speak to viruses as facilitators of gene transfers between cellular life forms during LECA times; they are not saying that viruses themselves were somehow important players in evolution as discrete biological entities with stable gene contents and metabolisms that might be relevant to how different types of cells / biological entities came together and gave rise to eukaryotic cells. This tempered conclusion (which I think is appropriate) is in contrast to the sentiments of some of the papers cited, such as Guglielmini et al. 2019, Forterre and Prangishvili 2013, and Forterre and Gaia 2016. This last paper discusses the viral eukaryogenesis theory (and shows a figure illustrating how an engulfed virus might have given rise to the nucleus.). It may be the case that the viral factory gave rise to the protonucleus. If so, given everything we know about the genes extant genes viruses contain and how viruses evolve today, I see no evidence that we can obtain results for or against these hypotheses.

Let me apologize for this rant about viral evolution, but I think it's really important to get right in a paper like this. If over interpreted or misleadingly summarized, there is a danger that it can be picked up on too strongly by others. So to conclude: I would encourage the authors to reconsider how the viruses feature their analyses and whether or not they are confident that the data in hand actually have something meaningful to contribute to the eukaryogenesis question.

Specific additional comments.
-Line 41/42 – cites references 14-16, some of which were published long before the discovery of the second of the two "established partners", i.e., Asgard archaea. So I would suggest dropping them out. They are not relevant to the specific phylogenomics question at hand.

-Line 49 – archaeal not archeal

-Line 59 – purged OF low-quality sequences…

-FLUAs, FLUOs and FLUPs. These acronyms are a bit cumbersome. I would suggest the authors define them again in the legend to Figure 1 so that readers don't have to go searching for them.

-Line 149 – reference for original description of the ESP concept (at least, when it became explicit in that way, presumably the very first Ettema papers on Asgard).

-Line 333 – "Nucleocytoviricota, also referred to as nucleocytoplasmic large DNA viruses, is a phylum of viruses known to infect diverse eukaryotes and to comprise giant viruses with large genomes39." Nucleocytoviricota includes some 'giant' viruses with large genomes but it doesn't comprise them. As the authors are no doubt aware, the consensus now is that giant viral genomes evolved multiple times independently from ancestors with much smaller genomes by gene acquisition, mostly from their hosts (related to comments above).

- Fig 2b/c. What is the black line connected with dots in part b, decreasing from low to high stringency? It is the number of trees, right? (labeled on the right part of the graph). Is there a way to make this more obvious (e.g., "Stress test for the number of trees (black dot connected by line)? As is, there is a lot going on in the figure, and it doesn't help that the color coding for part C is also used for part B (and includes a dark gray (not black) LECA box, which I can't see anywhere on the plots, nor the light gray control, which is I believe the point of these 'negative controls').

Fig 2c. "This is in stark contrast to the observed relationships of LECA genes with bacterial ancestors (Fig. 2c), indicating ancestries that cannot be explained by vertical inheritance through the mitochondrial ancestor." Where are the bacterial signals here, beyond Alphaproteobacteria? Again, is the point that they are not there in Fig 2c? This is important to know, as the conclusions on lines 213-218 mention Thermoproteota (part a, 2%). The language here is very important to get right for the reader to grasp the take-home message.

Fig 3a (and b). I'd suggest making the euk, mitoanc., and Asgard colors jump out more in part a relative to the other bacterial groups. Currently Fig 3b is described in the text before Fig. 3a, and it doesn't help that the color coding appears at the very bottom of the figure, under part d (I didn't notice at first). Readers will also be confused by use of "Thermoproteota" in the main text but not in the figure. What does this term apply to, exactly? Domain Archaea? The former Crenarchaeota? Clarity needed.

Line 232. "Our results (Fig. 3b, Extended Data Fig. 2) depict distinct branch length distributions peaking at different values, suggesting different relative timings." As far as I can tell, Fig 2b is simply a schematic representing trends from a microbial mat study published by Gutierrez-Preciato 2018. Did you mean to say Fig. 3a here?

Fig 3. The term Arcancestor is used here in two important places. What does the term mean, exactly? It's not used in the main text. Blue refers to Asgard (part a), Archaea (part b), and Arcancestor (parts c and d) all in the same figure. Non-specialists will find it difficult to connect all the dots, in part because the color coding is a tiny afterthought at the very bottom of the figure.

Fig 3c. "Proportion of transferred GENES from the donor

Fig 3c. Nucleocytoviricota. Chromatin (top line). Why does the line not have a circle? Why does it extend beyond 5 on the horizontal axis?

(Remarks on code availability)

Referee #4

(Remarks to the Author)
In this article, Bernabeu et al. reconstruct the proteome of the last eucaryotic common ancestor (LECA) based on publicly available eucaryotic genomes, functionally annotate it, and then investigate the origins of the LECA proteome using phylogenetics. In particular, they use a methodological approach that points at different potential contributors aside from the Proteobacterial ancestor of the mitochondrion and the Asgard ancestor. Their phylogenetic analyses point at six major contributing lineages: Alpha and Gamma- Proteobacteria, Myxococcota, Planctomycetota, Asgard and Nucleocytoviricota (a lineage of giant viruses). They reconstruct the core genome of potential contributors from the different lineages and establish their "metabolic profile". The authors also revisit from their data the list of the eucaryotic specific proteins (ESP). Overall, this study finds that there is the same level of evidence for other bacterial and viral contributions than the ones generally acknowledged from the Alphaproteobacterial mitochondrion ancestor and the Asgard.
This study builds upon a previous study published in 2016 in Nature by Pittis and Gabaldón where a late acquisition of mitochondrion was postulated, and where the contribution of several other prokaryotic lineages was pointed. Some of the methodology from this previous work was reemployed here ("stem length analysis"). The novelty here resides on a systematic investigation of the origins of different contributions to the LECA proteome, which is made possible by the availability and efficient analysis of datasets covering much more of the biodiversity than the ones employed in similar studies before. It also comes from the assessment of several acquisition waves by LECA, from different donor lineages.
In general, I found the article very well written, and the dataset gathered seems very solid considered available genomic data for both procaryotes and eucaryotes. I found the conclusions drawn well balanced and supported by the data. The topic is of exceptional interest to the readership of Nature and in a context of a highly debated process of eucaryogenesis. However, I had some methodological concerns and questions that I would like to discuss. Moreover, I believe that the manuscript would benefit from clarifying further in the text what truly constitutes novel findings compared to those from the Pittis and Gabaldón paper from 2016.

Major comments:

- My main concern comes from the use of the stem length analysis. To me it is unclear how this length can really relate to the timing of acquisition. Once acquired, a gene can endure different selective pressures that could temporarily affect its evolutionary rate before it is fixed, and I don't see why this force should be constant and homogeneous among different gene families, as it would depend on the function of the gene acquired and the genetic background of the receiving ancestral organism. Could the authors elaborate on this, and explain why they think selective pressures would not affect the stem lengths or would be consistent across the different waves of acquisition? How could this impact the relative timing of the waves measured? Have the authors tested for instance if the stem length varies between different functional categories? Could this be disentangled from having more or less ancient donors from a same lineage?

- If one wants to infer that a protein was ancestrally found, one needs to show that the protein phylogeny follows the species phylogeny, indicative of vertical inheritance. Regarding the phylogenetic analyses of the origins of the LECA proteome, it seems that there is no real "sanity check" of the eucaryotic phylogeny in the eucaryotic part of the tree. Could the authors elaborate on the reasons why they think that a protein was vertically transmitted from LECA to contemporary organisms?

- I do not fully understand the rationale of looking at sisterhood of LECA-inferred proteins in phylogenetic trees to find donors, and not try to find also "LECA-inferred" proteins within a monophyletic clade of bacteria (as opposed to sister to), as a proof for bacterium-to-eucaryotes transfer. Usually, "received" genes descending from an ancestral transfer form a clade nest within the (broader) donating lineage. How often was this found? Does the proposed method also consider those cases? How can this work in a context where the root of the gene trees is unknown? Otherwise, do the authors consider that the transfers are so ancient that the respective last common ancestors of contemporary prokaryotic lineages are all younger than the ancestral donating procaryote?

- What was the rationale behind the artificial rooting of the gene trees (e.g. line 680-681)? This was already discussed in Supplementary Discussion. Could the authors explain briefly in the main text how the root picked could influence the assignment of the LECA proteome origins, or how the controls made help ensuring the methodology is robust?

- Regarding the contribution of viruses (but this also apply to procaryotic contributions), how to make sure of the directionality of the transfers? Or what points toward a virus-to-eucaryote rather than the converse? This could be clarified/discussed in the text.

- This work points at several waves of transfers to the ancestor of LECA, yet no specific case is directly shown using phylogenetic trees. I think it would be important to convince of the credibility of the methodology, to display and discuss several cases of modules transferred, and how it reflects in phylogenetic trees. It could be from some of the cases further discussed in the Supplementary Discussion?

Minor comments:

- Some of the interpretations would require revising the text to make it more justified and clearer. For instance on lines 215-216, it is unclear why the authors consider that Gammaproteobacteria signals may actually be Alphaproteobacterial. And on lines 214-215, why Thermoproteota signals are interpreted as Asgards'.

- I think it would be great for the non-specialist readership that is not aware of the recent literature to instill more clearly what constitutes truly a novelty here when compared to previous work, in particular what is a new hypothesis or constitute new results, and what previous hypotheses are challenged by this work. To me, this is not enough emphasized as is.

- It is said on line 607-608 that one genome per genus from the GTDB was used and the ones with maximal completeness/minimal contamination selected. Could the authors please indicate the range of genome quality for the MAGs used in the end? Was a threshold of genome quality applied?

- For clarity, could the authors display on Sup Fig 1 the number of genomes available for the different eucaryotic lineages?

- On a same note, could a Venn diagram representing the different eTOLDB datasets added to the sup data? This would help to apprehend the overlap of the A, B, C datasets.

- Lines 738-739, maybe cite the supplementary tables?

- Sentence on lines 389-842: please rephrase to make it clearer.

- Figure 3D, please increase the size of the legend for taxonomical groups at the bottom.

- Ext data fig 4: would have been great to indicate more clearly the donating lineage to LECA.

(Remarks on code availability)

Referee #5

(Remarks to the Author)
A SUMMARY: The manuscript reports a reconstruction of the last eukaryotic common ancestor's proteome, and traced back the most likely phylogenetic origin of each protein family. They show evidence for several waves of horizontal gene transfer from diverse bacterial and viral donors some of which apparently prior to the mitochondrial endosymbiosis.

B ORIGINALITY & SIGNIFICANCE: This manuscript makes a considerable contribution to understanding the evolutionary origin of eukaryotes. Using the latest genomics data, it provides an updated perspective on eukaryogenesis, reinforcing existing models of serial symbiotic interactions with a diverse array of bacterial, archaeal, and viral taxa. It also offers more precise insights into the relative contributions of prokaryotic donors and their taxonomic affiliations than previous studies.

C-F METHODOLOGY, STATISTICS, UNCERTAINTIES &:The methodology appears robust, and the conclusions seem well-founded, often discussing alternative interpretations.

G REFERENCES: The manuscript relies heavily on self-citations. More credit should be given to previous work by others (see below).

H CLARITY & CONTEXT: Abstract and introduction are clearly presented. In certain places, the results and conclusions are not sufficiently clear (see below).

DETAILED COMMENTS AND SUGGESTIONS:
Line 19-20 Throughout this section and the entire manuscript, prioritize citing early foundational papers on the topics instead of relying heavily on self-citations.

20 Numerous papers highlight the involvement of other bacterial partners and viruses in eukaryogenesis. Clarify that the debate is not about their involvement but about the specific type of cellular organisms involved.

48-51 Rephrase the sentence to improve clarity.

115 To improve readability, the figure legends should spell out abbreviations.

120 The Suppl Info file should discuss the sometimes considerable difference between the frequencies in LECA and FLUPs (e.g. signal transduction).

159 Instead of a textual explanation, depict graphically the size of the Innovation rectangle. Also, indicate and graphically represent the affiliation of the remaining bacteria-like (23%), archaea-like (4%) and virus-like (4%) proteins (e.g., as unresolved).
Indicte the actual nr. of proteins within the rectangles of 'Acquisition', 'Innovation', 'Bacteria', Archaea', and 'Viruses'.

162 The description of fig.2b should be clarified. Explain the y axis and the black dots.

169 Legend 2c would benefit from a more detailed and clearer explanation.
The term 'verticality test' needs to be defined.

175-195 This paragraph, along with the unclear legend of Figure 2b, is difficult to follow.

197-220 Again, this paragraph, along with the unclear legend of Figure 2b, is difficult to follow. Also consider exploring alternative explanations for the findings.

202 Give examples of the closest sister groups.

205 This conclusion needs more explanation.

223-287 This text does not fit under the header 'metabolism'. One could introduce a separate section with its own header.

232 Fig. 3a (not 3b).

237 The 'longer lengths in Thermoproteota' is not obvious from Fig.3a.

252 Legend lacks essential details and lacks clarity ('of the mode'?; 'mitoanc';)
Specify the dataset to which the shwon distribution ordering corresponds, and the datasets for which it is different.
Otherwise, the figure is misleading.
The color scheme of taxa is too small, barely readable.
Thermoproteota, mentioned in the text, are not referred to in the figure.
Explain abbreviations in d:('Aut', 'Het').

278 Cite literature or mention earlier findings indicating that functions acquired from the one or other taxon are enriched in core or metabolic functions.

351-389 The discussion largely repeats ideas already discussed in the Results section.

365 The manuscript reports evidence that eukaryogenesis involved more partnes than Asgard ancestor and an alphaprotobacteria, a view that has been brough forward earlier and by other researchers. Therefore, for clarity and fairness, it should be stated which of the current models of eukaryogenesis the presented data fit (instead of stating which they do NOT fit).

376-391 This paragraph would benefit from more conciseness, and elimination of ideas mentioned in the results section.

393-398 Repeats ideas discussed in various places earlier in the manuscript.

(Remarks on code availability)

Version 1:

Reviewer comments:

Referee #1

(Remarks to the Author)
The authors made a good effort to address and clarify the concerns and comments raised by the reviewers, and as a result the robustness of the analyses/results and clarity of the manuscript has improved significantly. Still, the following comments are insufficiently addressed to warrant publication:

1. Metabolisms of prokaryotic donors
- The authors maintain that the metabolism of the selected taxa that contain the donated genes is reminiscent of the ancient donor. This remains a weak point in the manuscript (despite the context provided in the response letter). The limitations of the approach employed by the authors should be explicitly mentioned in the text, indicating that more appropriate methods exist to infer ancient metabolic properties (ancestral state reconstruction).
- To illustrate the point above: Assuming most genomes encode at least 50% of inferred donated genes, wouldn't this analysis bias the inferred metabolisms to Lokiarchaeia (most genomes/GTDB genera) for the Arcancestor and Rhizobiales for the Alphaproteobacteria? Please clarify.
- Furthermore, it is unclear which genomes were used for the inference of the metabolism of prok donors ("donor-like genomes" not found in Zenodo data, numbers also not mentioned, only 162 for the arcancestor in the supplementary discussion). Please provide this data.
- Stating "metabolic potentials of the major donors" in the abstract is a too strong statement in light of the limitation of the

approach used by the authors (above). Please tone down or remove.

2. Speculation about models for eukaryogenesis
- The authors should not confuse 'genetic contributions' with 'stable interactions'. This should be adapted/toned down in the discussion section, in particular the added section in lines 381-388. Genetic contributions? Yes certainly! Stable interactions? Symbiotic interactions could explain this, but alternative scenarios could also account for the observed patterns. It is inappropriate to discredit eukaryogenesis models that only include 2 partners (archaeal host + bacterial endosymbiont) based on the provided evidence.
- Regarding alleged Planctomycetota interaction: the explicit mention of phagocytosis in regard of eukaryogenesis models should be toned down given the limited distribution within planctomycetes (1 genus) and lack of knowledge about which genes are involved in this process. Why not simply mention that Planctomycetota and Myxococcota as potential symbionts instead, without inferring endosymbiosis for which this analysis does not provide any evidence?

(Remarks on code availability)

Referee #2

(Remarks to the Author)
I thank the authors for providing the code and extra supplementary materials through the updated Zenodo link, and for their responses to my concerns. However, several of my major concerns remain, and while I appreciate the authors' efforts to address them, I still feel that these do not go far enough to produce results that are sufficiently robust to support the claims made.

Major points

1)Data availability and referencing

I believe something's gone wrong with the supplementary material file names, information or both. I wanted to look up TATA element modulatory factor (shown as a eukaryotic innovation, K20286 which is correct), and found the annotation listed as:

mLECA_OGs/annotation/TOLDBA_LECA_KO.tsv:OG0009484|LECA_1 K20286 14 14.0 1.0 1.0
mLECA_OGs/annotation/TOLDBA_LECA_KO.tsv:OG0019899|LECA_1 K20286 7 7.0 1.0 1.0
mLECA_OGs/annotation/TOLDBB_LECA_KO.tsv:OG0005880|LECA_1 K20286 25 25.0 1.0 1.0
mLECA_OGs/annotation/TOLDBC_LECA_KO.tsv:OG0007360|LECA_1 K20286 18 18.0 1.0 1.0

But then for corresponding alignment files, using

ls mLECA_OGs/alignments/TOLDB*/*/OG0009484*

I find only mLECA_OGs/alignments/TOLDBC/Folder_6/OG0009484_1.final.metalign , and for

ls mLECA_OGs/alignments/TOLDB*/*/OG0019899*
, nothing. When I check mLECA_OGs/alignments/TOLDBC/Folder_6/OG0009484_1.final.metalign, that seems to contain sequences of bacterial carboxylases. For LECA_OGs instead of mLECA_OGs, it's similar. I chose another example at random, VPS8 (K20178), and in the alignment files for this found what seem to be bacterial Condensation domain proteins (OG0021610) and Argininosuccinate synthase (OG0002568), respectively. So this seems to be generally a more widespread issue with the supplementary materials; I hope it will turn out to be a simple naming problem with an easy fix, but the authors should check to ensure that it hasn't affected parts of their overall conclusions. (The Readme file also says that the annotation files contain information on the donor lineage for the mLECA_OGs, I didn't see that.)

The authors should more often directly reference supplementary tables and supplementary files that provide data underlying each of their claims - including those claims for which main or supplementary figures only are currently referenced. This should be done consistently throughout the main and supplementary texts.

The authors mention testing different parameters for phylogenetic reconstruction, and finding little difference between these results and their previous results, but the raw data underlying these finds don't seem to be available in the zenodo repository.

2)Concerns about the reconstructed LECA proteome

The authors' draft manuscript, as currently presented, would have two major groups of findings: a) those relating to the donors of different groups of eukaryotic genes; and b) a reconstruction of the LECA proteome, including an updated set of Eukaryotic Signature Proteins.

To a concern about annotation of the LECA proteome that pertains to the quality of b), the authors respond that "We [...] hope the reviewer understands that careful manual curation of the potential ancestral functions of hundreds of protein families considered in this study is beyond the scope of this type of analysis as it requires time and expertise beyond the

reach of a single research group"

I completely understand and sympathise with this; indeed, my original review included acknowledgement of this ("if a LECA proteome is being presented, then the functional work needs more specialized curation/interpretation by collaborators familiar with the specific pathways/cellular functions under discussion (this is too much work to realistically expect the small number of authors here to do well)"). I fully acknowledge the amount of work that has clearly gone into this section - on top of all of the other work in the paper! Nor can any LECA proteome reconstruction be perfect. However, any work claiming to be an updated LECA proteome published in Nature will be used as a reference by Nature's broad readership (especially as it is absolutely not trivial for the readership to double-check the results from this manuscript, given the amount of data to wade through), and so it must be reasonably reliable and robust. At this time, EggNOG's approach, while still the best automated tool available, fails for the many gene families in which correct orthology assignment is difficult because domain shuffling has taken place, or where lower than average levels of sequence homology are present. These include gene families with major implications for the functional potential of the LECA, such as those involved in membrane deformation, adhesion or signaling. Until better tools are available, manual curation that employs expert knowledge of the underlying biology, with careful presentation of the results in a manner that is informative to both specialist and non-specialist audiences, is therefore still essential (which as the authors agree is indeed beyond the reach of a single research group).

This also extends to discussion of the topic: currently, this section in the supplementary materials lacks almost any citations of or discussion of previous work on the origins and evolution of these pathways. This is inadequate scholarship, and doesn't allow the readers to compare the authors' results with what might be expected from earlier work. The vague wording also makes it difficult to double-check the results from the authors' own analyses, or even to understand what is meant in some cases. Supplementary Tables 4 and 5 are referenced, but these don't mention gene names, so that when the authors discuss (for instance) "We inferred a LECA that also had a basic cell adhesion mechanism" or "Our LECA model contains most of the genes that are predicted as shared between Human and the algae C. reinhardtii and were obtained through a combination of acquisitions and innovations [no citations]" or "Our LECA inference depicts almost complete modern DNA and RNA processing systems (e.g., polymerases, transcription factors, ribosome, etc.) with some specific RNA polymerases missing", with no further detail given in the supplementary tables referenced, their use to the reader is very limited and must be largely taken on faith. The 'signaling and adhesion' section mentions adhesion proteins and quorum sensing proteins - the latter of which is really interesting if true - but I can't check these results as they aren't mentioned anywhere in the supplementary tables, and no pointers to this evidence are given in the text (no supplementary files are referenced, no gene names or orthogroup numbers are given).

As a related point, Supplementary Table 9 mentions annotations of new putative Eukaryotic Signature Proteins, which the authors define themselves in their table title as "Innovations are those KOs annotated in trees of any database and for which *we do not find any non-eukaryotic homolg* [sic]" This title is immediately contradicted by the table itself, which lists the KO MRCAs for just over 6% of these as 'LUCA', as they are found in Bacteria as well as eukaryotes (these still include photosystem genes, as well as kynurenine 3-monooxygenase, aldolase and other genes that are notoriously not eukaryotic innovations). Clearly there is an issue in the overall annotation process, either with the OG analysis underlying these data, or with the KO MRCA. Since this affects at least 20% of the annotated pathways even after reannotation, it therefore calls into question the paper's conclusions with regard to the ancestral proteomes and their functions overall (of the LECA but also of the prokaryotic donors).
This goes back to my point in my initial review that bacterial proteins were among the supposed ESPs. The authors countered that this was down to "current biases in annotations, which are mostly based on a few model organisms" - but if the genes under discussion are supposed not to have any non-eukaryotic homologs (by the authors' own definition in the table legend and given their use of the term 'innovation'), then it shouldn't have been possible for these to be misannotated as prokaryotic proteins. It's possible that the table simply mistakenly included non-eukaryotic innovations and that every line with LUCA as the KO MRCA should have been removed; but then the number of ESPs should be updated in the text. I also don't believe that these are the only genes affected: at minimum the ribosomal proteins listed aren't all eukaryotic innovations (RPL28 is in at least Heimdallarchaeota, RPL36 and RPS7 are found in bacteria, RPL29 is an ortholog of bacterial RPL35; hedgehog family proteins arose from a domain fusion along the animal stem lineage, but if any hedgehog domain-containing proteins are being counted in this category, then it makes less sense to classify VPS proteins with domains found in Asgard archaea as eukaryotic innovations...).

Supplementary figure stpf10, the probability of various photosynthetic pathways having been present in the mitoancestor are shown as being higher than those for most amino acid degradation pathways, and on the order of that for F-type ATPase. If this is true, then it's a very interesting finding that should be discussed in more detail; but it's a lot more likely that these are pathway components that carry out non-photosynthetic roles, and are present in this capacity.

The claims presenting these findings as an updated LECA proteome and a new set of ESPs should therefore be removed from the abstract, main text, Figure 1 and supplementary discussion. The supplementary tables can still be included to aid in understanding and interpreting the authors' findings related to the donor lineages (a) in my description above). The authors are then free to use their reconstructions as the basis for subsequent high-impact work through the initiatives that they mention.

3)Phylogenetic methods

Supplementary methods, 'Parameters for tree reconstruction': if I'm understanding the authors right, they used the command shown in the box (iqtree2 -s {exp_LECA_og}.metalign.trim -T 8 --mset DCmut,JTTDCMut,LG,WAG,VT,LG+C10,LG+C20,LG+C30,LG+C60,LG4X,LG4M -m MFP -B 1000 --prefix

iqtree/{exp_LECA_og}) to assess the effects of including mixture models, but then chose to go ahead with trees computed without the C-series mixture models in the interests of time. It would be helpful for the reader to include the final command that was used for these trees. Was it iqtree2 -s {exp_LECA_og}.metalign.trim -T 8 --mset DCmut,JTTDCMut,LG,WAG,VT, LG4X,LG4M -m MFP -B 1000 --prefix iqtree/{exp_LECA_og})? The line "we decided to keep working with the current version of the trees inferred with the corrected command shared in the reviewed version of the manuscript" makes me unsure whether this was the case, or whether they used the trees originally computed for the non-revised manuscript.

More seriously, "The use of this option when using the stringent parameters used to select the donors (ufboot-bnni >= 75%, 7 supergroups, proportion of the donor in the sister >= 95% and taxonomic bootstrap >= 95%) caused a drop in the number of trees assigned to each donor. This drop was irregular between the sister groups ranging from 30% in Myxococcota and Nucleocytoviricota to 70% in Chloroflexota and Gammaproteobacteria. Despite this, and accounting for the overall reduction in the number of trees that pass the filters, the selected taxonomic groups remained stable.": the selected taxonomic groups remained stable, but the number of trees that pass the filters overall do not, and nor do the proportion of sister groups chosen. This is not something that the authors should simply be ignoring. This will affect the major conclusions (e.g. those shown in Fig. 2, Fig. 4; and since it affects the number of trees that pass filters overall, it is likely to also affect branch length distributions and therefore Fig. 3; Fig. 1 may also be affected, but for this, see my comments below). The authors should therefore use -bnni for all of the trees that are used to support their final conclusions. This is more computationally intensive, but appropriate given the need for robustly supported conclusions.

In their response to my comment on C-series mixture models, the authors state that "we did not observe differences in the results" between a set of trees produced in their initial analysis versus a set of trees computed with the option of these models. This is true only for the trees that finished running. This is an important distinction: I would expect simpler, less parameter-rich models to perform as well as mixture models for many proteins, but to be more prone to artifactual results, including artifically high support for incorrect topologies, in some cases. Those cases include particularly fast-evolving proteins, or datasets with some fast-evolving taxa (for instance, viral sequences), and I would expect the C-series models to run for longer in just such cases. This affects 4% of the phylogenies that the authors double-checked; how many of these included viral sequences? Primarily presenting results generated using a methodology that's less robust to artifacts over this time scale is in any case not very robust, but at minimum, the authors should mention whether the proportion of the dataset that could not be assessed in this way had any overrepresentation in terms of Nucleocytoviricota presence (Nucleocytoviricota are notably not mentioned in Supplementary Tables 11 and 12), or function.

Minor points

Lines 29-31, "our results suggest that ancient eukaryotes originated within complex microbial ecosystems through a succession of symbiotic interactions that left a footprint of horizontally transferred genes" - the authors have toned down references to symbiosis in their title and abstract, appropriately given the different possible uses of the term 'symbiosis' (as they have pointed out in their response) and the difficulty of interpreting the nature of the relationships between gene donors and recipients in ancestors of eukaryotes. This sentence should also be changed to be consistent with this: "our results suggest that ancient eukaryotes originated within complex microbial ecosystems through a succession of interactions that left a footprint of horizontally transferred genes". Speculation on the nature of the interactions can then be had in the discussion.

Lines 40-41, "a host with an Asgard archaeal component", "a host with an Asgard archaeal affiliation" or "a host with an Asgard archaeal ancestry" would be more accurate here

Line 94, "Our reconstructed LECA proteomes comprised an average of 10,255 or 5,995 mLECA-OGs" - the appropriate supplementary materials supporting these figures should be cited here

Line 351-352, "being the detected specific contributions from Alphaproteobacteria (8%), Planctomycetota (8%), and Asgard archaea (4%)" should read "with the detected specific contributions being from Alphaproteobacteria (8%), Planctomycetota (8%), and Asgard archaea (4%)"

Line 361-362, "The different mechanisms evolved mainly during eukaryogenesis" - we can't be sure of this, as a significant amount of time elapsed between eukaryogenesis (whatever criteria for this are used) and the emergence of the LECA. Furthermore, the authors' own conclusions here and in previous work suggest that eukaryogenesis may have been a protracted process that may have been largely complete by the time of the mitochondrial symbiosis. "The different mechanisms evolved mainly in the eukaryotic stem lineage" might be closer to what the authors intended.

Supplementary figure stpf10, a couple of typos: Histidine degadation (should be degradation); Hydroxypropionate-hydroxybutirate cycle (should be Hydroxypropionate-hydroxybutyrate)

(Remarks on code availability)

Referee #3

(Remarks to the Author)
The authors have provided thoughtful, well-reasoned responses to the comments of five reviewers and done a very thorough revision of their manuscript. The improvements are, in my opinion, substantial. There is now more clarity around various key

elements of their results and interpretation, including the stem length analysis; this will provide clearer substrate for future discussions and, I suspect, debate. The tweaks to the viral contributions to LECA are well-received (namely addressing the question of directionality); the data say what they say, and I think the authors now present them in a way that is less open to over interpretation.

Having looked at the full set of comments and revised manuscript, I am left wondering about the revised title, and the broader take-home messages from the revised data / results / interpretation. The authors have removed 'symbiotic', which leaves:

"Diverse ancestries reveal complex interactions during eukaryogenesis"

I think this is not sharp enough to be useful.
Diverse ancestries of what? (inferred LECA genes?)
What type of interactions? (syntrophic? Or any and all types of interactions as long as they are 'tight'?)

I would encourage the authors to think of a more explicit description of their results.

The authors feel that I (and perhaps R2) were overly focused on endosymbiosis, which is fair enough. In their rebuttal, they talk about their intended (and more generic) usage of symbiosis, i.e., "tight co-existence". So what does that mean, exactly? Readers will want to know.

And regardless of the intended meaning of 'symbiosis', when it comes to a multiple "acquisition waves" model, the authors are still invoking multiple, sequential 'tight co-existences', between different cells at different time points -- each making their mark in sequence on a proto-eukaryotic cell somewhere between FECA and LECA.

So removing 'symbiosis' from the title does nothing to improve clarity, since the intended meaning is (sort of) still retained in the abstract ("succession of symbiotic interactions...") and elsewhere.

I'd encourage the authors to give the title a rethink. If metabolic interactions is what they feel their results speak to, then may 'syntrophic interactions' is the answer.

(Remarks on code availability)

Referee #4

(Remarks to the Author)
I would like to thank the authors for the thorough rebuttal letter, which provided very useful information based on my own comments and those of my colleagues. I am very satisfied with answers to these comments and with the comprehensive experiments that were added to address them. This is reflected by extensive text revisions and additional supplementary texts and figures, and I judge that the manuscript was well improved in the process, gaining depth and clarity. Hence, I have no further comments, and I congratulate the authors on their hard, interesting work that - I think - opens many new perspectives for future studies.

Minor comment/typos
- In the figure Ext. Data Fig. 2 (Line 837) => “Alphaproteobacteria” is misspelled.
- Of note however, the Github page link was not working when I attempted to test it:
https://github.com/Gabaldonlab/LECAdonors
- The Zenodo page was available, but with restrictions on its content, which is not practical for review of the file contents.

(Remarks on code availability)

Referee #5

(Remarks to the Author)
This second version of the manuscript has clearly improved by putting the work better in the context of ongoing work by others in the field, and providing a more critical assessments of results.

Summary of key results
The manuscript reports a reconstruction of the last eukaryotic common ancestor's proteome, and traced back the most likely phylogenetic origin of each protein family. They show evidence for several waves of horizontal gene transfer from diverse bacterial and viral donors some of which apparently prior to the mitochondrial endosymbiosis.

Originality & significance
This manuscript makes a considerable contribution to understanding the evolutionary origin of eukaryotes. Using the latest genomics data, it provides an updated perspective on eukaryogenesis, reinforcing existing models of serial symbiotic interactions with a diverse array of bacterial, archaeal, and viral taxa. It also offers more precise insights into the relative contributions of prokaryotic donors and their taxonomic affiliations than previous studies.

The methodology appears robust, and the conclusions well-founded.

References
However, more credit needs to be given to previous work. The concluding statements of the manuscript (L373-388) give the impression that this is the first study to suggest a role for viruses, -and specifically Nucleocytoviricota—, in the transfer of non-eukaryotic genes to LECA. This claim appears inaccurate. Relevant prior studies by other researchers should be cited, and the findings of earlier work should be thoroughly discussed, particularly in terms of the extent of gene transfer proposed and the methodological robustness of the conclusions.

Clarity
Several places in the manuscript lack precision, which makes it difficult to read. E.g.,
- L 79 'alignment profile searches'. The type of the profile and the searches should be briefly specified.
- L238-240, to be rephrased.
- L363: Fig. 1c seems not to depict the assertion preceding the reference to this figure.

Several grammatical errors were spotted, e.g.,
- L128 'extant genome size' -> proteome size of extant FLUs
- L149 It is indicated that Fig. 2 shows topologies or sistergroups, but it doesn't.
- L160 'impact of ..thresholds "ON" not "in". Does 'trees' mean 'OGs'?
- L207 'trees... were of origin..' rephrase.
- L254 'with' -> 'from'
- L278-280 check
- L284-285 check
- L351 check

(Remarks on code availability)

Version 2:

Reviewer comments:

Referee #1

(Remarks to the Author)
The authors have done a great job to accommodate the comments and suggestions that were brought forward by the reviewers. I think that overall the presented work is convincing, and the discussion about the results is in balance. Eukaryogenesis remains to be a vibrant topic, and I am convinced that the work by Bernabeu et al will, besides providing new insights, also spark new scientific debates. As such, I look forward to seeing this manuscript appear in print.

(Remarks on code availability)

Referee #2

(Remarks to the Author)
I would like to thank the authors for addressing my comments. I am glad that using mixture models helped to improve the robustness of the authors' results, as strengthening the authors' case for their results has been my aim. I acknowledge the significant amount of time and effort that this has involved on the authors' part, and commend them for doing it. As the authors point out, I am the only reviewer to bring up some of these points; certainly other reviewers noticed plenty of points that I missed, that is part of the point of having multiple peer reviewers. My aim with my comments is not to attack the authors, who have done a tremendous amount of work, but to ensure that this work is presented completely and robustly, as it deserves.

* What kind of decontamination, if any, was done on the eukaryotic datasets that were retained for the three eTOLDBs? It looks to me as though the finding of photosystem genes in the LECA (!) in Table ST4 is being driven by contamination from land plants in the ARCVU, LON04 and HET04 datasets (the first two supposedly amoebozoan, the last heterolobosean, but yielding sequences >= 99% similar to plant proteins).

I would like the authors to take the time to look into this more deeply rather than simply discarding the specific orthogroups in question, and to explain briefly how they then ensured that the eTOLDB databases then no longer contaminated, in order to ensure that any other contamination in these and/or other datasets isn't affecting wider conclusions regarding LECA donors. I say this because there are known to be issues with cross-contamination or abundant prokaryotic contamination in some datasets from poorly studied microbial eukaryotes.

* I'm finding it difficult to find the source data underlying the donor KOs and modules given in the donors_reconstruction folder (i.e., which sequences give rise to which KO numbers); it doesn't seem to be as simple as cross-referencing

donors_reconstruction/donor_genomes.tsv with the donor KOs information found in mLECA_OGs/annotations/ (at least, not with regard to the mitoancestor)

* I very much appreciate that the authors have added a degree of 'sanity checking' to the data and more detailed discussion regarding the LECA proteome, including more citations. I appreciate the authors noting in their response that the new filters seem to have modified some of the putative LECA functions that they had previously mentioned. I would like to reiterate that my concern hinged on the fact that there would most likely have been other such examples that I was too unfamiliar with to be able to comment on.

A major concern of mine in this section was precisely because it seemed to me that a small but significant proportion of misidentified LECA proteins could point to issues affecting conclusions with regard to the ancestry of the LECA proteome as well. The authors state that "To minimize annotation artefacts we used a taxonomy-informed approach, assessing the taxonomical breadth of proteins annotated as each KO in KEGG and removing KOs that were domain-specific to Archaea or Bacteria, or that were too restricted to a specific eukaryotic group (e.g. mammals) (see Methods), therefore restricting the annotation to KOs that were either unique to eukaryotes but broadly covered within them, or KOs that are widespread among both eukaryotes and prokaryotes", so I would just like to know whether removing these proteins affects the set of putative LECA proteome donor lineages.

If these aren't significantly affected, then since the abstract and major messages of the paper highlight LECA proteome ancestry rather than its specific composition, I am going to simply request the following change:

The authors point in the supplementary discussion, "We thus stress that the purpose of this reconstruction is to approximate gene families and functional categories likely present in the ancestral LECA proteome, rather than to provide a carefully curated and detailed picture of LECA's traits" is an important qualifier, and I would like to see it added to the section on the LECA proteome in the main text. I do not feel that this will invalidate the authors' hard work on this section, or the main messages of the paper.

Minor comments and suggestions

- Regarding another reviewer's comment, the Zenodo link that the authors provided in their response didn't allow me access, but the link provided in the main article file did allow me to download all of the files together (though not to download each file individually).

- Discussion of the LECA proteome will necessarily need to be limited (despite the amount of work that the authors clearly put into expanding the discussion!), but I did find it striking that mitochondria weren't mentioned in the LECA proteome discussion section at all. I don't have strong feelings regarding whether these are discussed specifically or not, but I would like to ensure that mitochondrial proteins weren't accidentally discarded as the result of one of the filtering steps, and it would probably be helpful to the reader for the authors to at least mention whether discussion of them was omitted in the interest of space or for another reason.

- Line 116, "suggesting that LECA was most likely aerobe" should read, "suggesting that LECA was most likely an aerobe"

- The Table S6 legend states that "We further restricted to those KOs that are pan-eukaryotic according to KEGG", but some entries, despite having a KO MRCA in eukaryotes, mention only animal and fungi KEGG kingdoms, so I'd suggest clarifying in the table legend that the eukaryote MRCA was used as a criterion (presumably).

- For discussion of gene transfers mediated by members of the Nucleocytoviridicota, I'd suggest also citing Karki et al. 2025 https://doi.org/10.1007/s00239-025-10246-8 .

(Remarks on code availability)

Referee #3

(Remarks to the Author)
I thank the authors for their thorough second revision and response to reviews. I am satisfied and have no further comments on the manuscript.

(Remarks on code availability)

Referee #4

(Remarks to the Author)
I would like to thank again the authors for the extensive revisions and discussions provided across the rounds of revisions, the manuscript is much improved and present clearly the data and results, while offering a balanced discussion on the limitations of the methodology. I also agree with the new version of the title.
This work constitutes a major contribution to the ongoing debate on eukaryogenesis.

I have no further comments, except for a typo on line 351: « Desulfobacterota » is the correct name for the phylum.

(Remarks on code availability)

Version 3:

Reviewer comments:

Referee #2

(Remarks to the Author)
I would like to thank the authors for the extra steps taken to decontaminate their data. I acknowledge that this is a great deal of work, and share the authors' frustration that at this time, biological, methodological and taxon-sampling limitations mean that data from most eukaryotic groups must be treated with caution.

However, the decontamination analyses have been performed as a post hoc step, rather than being incorporated into the results shown in the manuscript or the supplementary files provided: the numbers of mLECA-OGs reported remain identical in this revised version of the main manuscript, as do the contents of supplementary tables 4, 5 and 7, supplementary folders with mLECA-OGs, inferred_metabolisms, and so on. The authors do provide lists of putative contaminated KOs and OGs in the zenodo archive, but the task of subtracting these from the results presented in the rest of the manuscript is left as an exercise to the reader.

I appreciate that the authors report that only a small proportion of KOs and OGs are affected, and understand their position that the overall conclusions are therefore not changed. However, showing analyses known to be based on flawed data is inappropriate, and it is quite unreasonable to expect the casual reader to cross-reference each of the reported results with the supplementary materials to infer the true result, free of contamination (especially when the main text gives no indication that decontamination was done post hoc - on the contrary, "To alleviate potential artefacts arising from phylogenetic reconstruction, including the effects of unsampled lineages, contamination, and recent HGT, we used state-of-the-art methodologies and compiled curated datasets" would lead anyone reading only the main text to believe that the data were throughly decontaminated from the start).

"However, we also found bona-fide LECA proteins such as the mitochondrial F-type H+-transporting ATPase subunit beta" - actually looking at the data makes it obvious why this was flagged as affected by contamination: OG0001062_3 isn't the mitochondrial F-type H+-transporting ATPase subunit beta, but the chloroplast one, with plant contaminants from amoebozoan and heterolobosean datasets. So, "reinforcing the abovementioned idea that eukaryote-to-eukaryote contamination detection is prone to false positives" - not really, if anything in this case it's underestimated the degree of contamination. I picked another tree containing some of the contaminated heterolobosean data at random, OG0010678, and found it was yet another gene of plastid origin with plant contaminants; this wasn't listed among the OGs affected by contamination. I am not performing a full analysis of the dataset, so if I'm (again) able to find this so easily through a random check, clearly there are more such cases out there, and the underlying issues have not been fully resolved. The failure to realise this by simply looking at the tree, instead choosing to go with an explanation in line with preconceptions, is to me emblematic of the problems with the paper.

I therefore recommend that the authors carry out a comprehensive reanalysis of the dataset, removing as much contamination as possible and following up on trees with possible indicators of contamination (e.g. very long branches, topologies suggestive of plastid origins). Without this, it will be difficult to be sure that the data underlying the paper's major conclusions on LECA protein origins are solid.

Minor comments

Line 114, I'd suggest using alternative phrasing to 'primitive'

Figure 2d, I'd suggest using a darker colour for 'Virus-mediated' as the current dark yellow is hard to read

(Remarks on code availability)

Version 4:

Reviewer comments:

Referee #1

(Remarks to the Author)
The authors have yet again have done a substantial analyses to alleviate concerns that were raised by reviewer 2 regarding potential effects of contamination in the data on the overall conclusions of the study. Even though I would like to underscore

that in my view it was unnecessary to do yet another re-analyses of the data (the authors had convincingly motivated that the impact of contaminating sequences would not violate their conclusions), I am happy to see that the latest analyses show that 1) the main conclusion that Myxococota, Planctomyctetota and Viruses have been genetic contributors to eukaryotic gene content remains robustly supported, and 2) that 'secondary signals' pointing to Gammaproteobacteria and Thermoproteotota (likely derived from Alphaproteobacterial and Asgard archaeal signals) were no longer significantly supported. I hereby conclude that any remaining concerns of Reviewer 1 have been convincingly addressed, and hope to see the revised manuscript published without further delay.

Minor comment:
Line 303: Change 'Heimdallarchaeota' into 'Heimdallarchaeia' (as is used in the rest of the manuscript).

(Remarks on code availability)

Open Access This Peer Review File is licensed under a Creative Commons Attribution 4.0 International License, which permits use, sharing, adaptation, distribution and reproduction in any medium or format, as long as you give appropriate credit to the original author(s) and the source, provide a link to the Creative Commons license, and indicate if changes were made.
In cases where reviewers are anonymous, credit should be given to 'Anonymous Referee' and the source.
The images or other third party material in this Peer Review File are included in the article's Creative Commons license, unless indicated otherwise in a credit line to the material. If material is not included in the article's Creative Commons license and your intended use is not permitted by statutory regulation or exceeds the permitted use, you will need to obtain permission directly from the copyright holder.
To view a copy of this license, visit https://creativecommons.org/licenses/by/4.0/

Response to Reviewers comments (our responses indicated in red color).

Referee #1 (Remarks to the Author)

The origin of eukaryotes represents an important knowledge gap in the evolution of life on Earth. Current data supports scenarios in which the eukaryotic cell evolved via an endosymbiosis between an archaeal host cell and a bacterial (mitochondrial) endosymbiont. Yet, many genes present in the Last Eukaryotic Common Ancestor (LECA) cannot be traced back to either of these two lineages, indicating that additional donors that contributed to the gene content of LECA. The manuscript by Bernabeu et al aims to trace the origin of these genes, reporting that the genome content of LECA and its ancestors evolved "through a succession of symbiotic interactions", a conclusion that is similar to previous work by Pittis and Gabaldon, and others, which proposed a that the LECA proteome had a chimaeric phylogenetic origin prior to the acquisition of the mitochondrial endosymbiont. The main novelty in Bernabeu et al involves the claim that a significant proportion of LECA gene content is seemingly of viral origin, as it seems to contrast with observations made recently by Irwin and co-workers (Irwin et al, Nature Microbiol, 2022), who reported dominant eukaryote-to-virus transfer, but relatively limited influx of viral genes during eukaryotic evolution. In addition, Bernabeu et al report specific bacterial contributions from Planctomycetes and Chloroflexi to the LECA gene content, something that was not observed in previous studies

If the above claims made by Bernabeu et al are correct, this would be relevant for scenario's eukaryogenesis. Yet, given that these findings seemingly contrast with earlier reports, the authors need to show that their results do not stem from methodological issues (see below). Furthermore, some parts of the manuscript are not particularly strong, and therefore misleading, and should be omitted, such as the inference metabolism of prokaryotic donors, and the discussion about biofilms and its relevance for eukaryogenesis.

Response: *We thank the reviewer for the generally positive comments. As you will see, we have now made additional efforts to show that the new findings stem from the increased amount of data considered rather than from methodological differences. In addition, we better explain that our results do not contradict those of Irwin and co-workers, but are rather complementary (see section "Virus as mediators of gene acquisitions during eukaryogenesis" in the main text). Finally, we provide a better rationale on the novelty and usefulness of the contributions presented here. We agree that the discussion on the biofilms and donor metabolism remain somewhat speculative, given the difficult question at hand and the very ancient times involved. We now better stress the inherent uncertainty and explain why these results can nevertheless provide interesting hints for discussion and future research.*

Main comments

Contrast with earlier studies

A main claim made in the paper involves that 10% of LECA genes are of viral origin, of which 6% stemming from Nucleocytoviricota. This finding seems to contrast with a recent study that specifically investigated eukaryote-virus HGT (Irwin et al, 2022), where relatively few transfers were observed from virus to eukaryotes (only several hundreds, but not restricted to genes ancestral to all eukaryotes).

Response: *We have now carefully re-assessed the involvement of viruses, in particular to not consider cases where the directionality of the transfer (euk-to-virus or virus-to-euk) cannot be confidently determined. This has resulted in the inclusion of an "unknown" category to define the uncertainty in the ancestry of mLECA-OGs that had only viral sisters or a few prokaryotic sequences representing low taxonomic diversity, in which case the directionality of the transfer cannot be confidently determined. This has resulted in a lower number of viral transfers, but a higher certainty on the directionality and the relative time estimate of the transfer. The amount of transfers potentially mediated by viruses among LECA acquisitions is still remarkable and still dominated by Nucleocytoviricota. We also explored the potential origin of transfers mediated by viruses by examining the clades that are sister to the LECA+virus clade, and we have identified the presence of the major prokaryotic donors, which reinforces the idea of viruses mediating such transfers. We now devote a specific section to the potential role of viruses mediating some of the observed transfers see "Viruses as mediators of gene acquisitions during eukaryogenesis" in the main text and "Nucleocytoviricota-mediated transfers to LECA" section in the supplementary discussion.*

We have carefully re-read the work of Irwin et al. and we are convinced that our results do not contrast with theirs. In fact, we think the two studies have different aims and are complementary. We have a specific interest in looking for evolutionary ancestries of LECA gene families, whereas Irwin et al. look at transfers occurring anywhere in the eukaryotic phylogeny. This means that i) only a very minor fraction of their identified transfers may correspond to a family that could be traced to be present in LECA, so our results are not directly comparable to theirs; ii) in their setting, as they trace any virus-euk transfer (either recent or ancient) regardless of the taxonomic distribution in eukaryotes or viruses and the directionality, it is easier to identify euk-to-virus transfers, as many extant eukaryotes are not prone to transfers (e.g. multicellular), whereas with our filters most such cases would be discarded (being viral sequences embedded within the eukaryotic phylogeny). Therefore our results and theirs are not directly comparable and one cannot be said to contrast with the other, as they look at different sets of virus-euk exchanges. Finally, although they identify a majority of euk-to-virus transfers, they do identify 849 virus-to-euk transfers after filtering versus 2,713 eukaryotes-to-virus, thus virus-to-euk represent a sizable (one fifth) of the identified HGT events, at any node of the eukaryotic phylogeny.

Finally, as we specify in the text, we do not exclude the possibility that the proteins transferred from virus to LECA were present before in other eukaryotes (relatives of LECA, also descendants of FECA that may have gone extinct), this is particularly plausible where we only identify a viral sister group without any prokaryotic outgroup. In fact we think our results do support a scenario where viruses were mediating transfers to LECA from prokaryotes and other proto-eukaryotes, and likely the other way around (which we cannot trace). We have now better clarified this by modifying the text and where we cite the paper of Irwin and colleagues.

Another remarkable finding represents that Planctomycetes and Chloroflexi seemingly have donated relatively large numbers of genes to LECA, considering the relatively low number of inferred acquisitions from Planctomycetota and Chloroflexi in Pittis and Gabaldon (2016) and Vosseberg et al. (2021). The question arises whether this could be a result of taxon

sampling (or renaming!), or due to the use of other methods? Given that the authors use a different approach to identify/classify LECA genes of non-eukaryotic origin, it should be ruled out that the obtained findings are the result of methodological issues/choices or definitions (e.g. taxon reclassification).

Response: *We now discuss our results in the context of previous studies and include new analyses in the supplementary discussion section entitled "Comparison with previous studies". We understand the concerns of the reviewer, and we assessed three possible sources of differences due to i) database composition, ii) taxonomic reclassification, and iii) methodological choices.*

i) For the database composition, in the 8 years that have passed since the analysis of Pittis and Gabaldón (2016) and this work, the taxonomic coverage of both eukaryotic and prokaryotic groups has tremendously increased, which was one of the motivations for this study. For instance, just in the last five years Planctomycetota has gone from 193 genomes in the release 80 of GTDB in 2019 to 2,450 in the 214 release, and Chloroflexota increased from 356 to 3064 in the same period. In the case of eukaryotic representation, recent advances in the sequencing of protist genomes have allowed us to increase the diversity of Amorphea by additionally including Apusomonadida and Breviatea representatives, as well as the diversity of TRASH (of which there is only Stramenopiles and Alveolata in Pittis and Gabaldón (2016), and we include Telonemia, Rhizaria and Haptophyta), as well as new lineages of Archaeplastida (Cryptophyceae, Glaucophyta,...) and Metamonada, and the addition of entire new supergroups not included in the Pittis and Gabaldón (2016) dataset, like PHM (Provora, Hemimastigophora included in our set), CRuMs and Ancyromonads. We conclude that differences in database composition are major and are the most likely source of differences, where our analysis - based on the most updated and comprehensive dataset - should be considered more accurate than that of Pittis and Gabaldón (2016).

ii) *Regarding taxonomic re-classification, we assessed the 692 taxonomic levels employed in Pittis and Gabaldón (2016) and assessed the affiliation of their genus at phylum level in the taxonomic framework employed by Pittis and Gabaldón (2016) (based in NCBI taxonomy) and the taxonomic framework of GTDB. Most of the phyla have a 1:1 correspondence, with only some exceptions (GTDB phylum "Thermoproteota" encompassing TACK, or Nitrospirae being split into different phyla within GTDB framework) and only a small minority of genomes changing phyla. Within phylum Proteobacteria, most alphaproteobacterial sequences remain Alphaproteobacteria within GTDB, Beta- and Gammaproteobacteria are joined into class Gammaproteobacteria, Zetaproteobacteria remains the same and the delta/epsilon subdivisions are split into different classes and phyla according to the GTDB species phylogeny. For instance, Myxococcota was previously placed within Deltaproteobacteria, while currently is considered a phylum. Thus, we consider that differences in taxonomic criteria are unlikely to drive major differences in the results, as they do not dramatically affect the discussed groups. Again, as taxonomic modifications are adopted to better reflect evolutionary relationships, our analysis should be considered more accurate as compared to previous ones.*

iii) As for methodological differences, the change that can have a major impact is the definition of LECA families, for which we use a criterion that is more stringent than the one used in Pittis and Gabaldón (2016) or Vosseberg et al. (2021). The topology of the

Eukaryotic Tree of Life has been modified in recent years in light of new data, with changes in the position of, among others, Ancyromonadida and Malawimonadida, which results in differences in the LECA criteria applied in Pittis 2016 and in our paper. For example, the Pittis and Gabaldón 2016 study would have considered a gene family present in Opisthokonta, Ancyromonadida and Malawimonada to be present in LECA, whereas with our LECA criterion they all belong to Stem 2 (Opimoda+) and would therefore be insufficient to be considered as present in LECA. We additionally set a minimum of 3 supergroups as threshold, whereas in Pittis 2016 2 supergroups were sufficient. Therefore, some differences in terms of LECA repertoire and taxonomic assignment of prokaryotic origins are to be expected. We note that Planctomycetota and Chloroflexi groups were also detected in Pittis and Gabaldón, and Vosseberg et al, albeit in lower proportions, but also those and other related studies had many more "unassigned" or broadly assigned origins (e.g. assigned to the ancestor of all bacteria).

Other methodological differences affect homology searches, phylogenetic tree reconstruction, and inference of potential donors. The referee is right that it would be good to know whether the differences stem from database composition or methodological variations. To assess this we have now run our current methodology on the tree dataset of Pittis and Gabaldón (2016). The use of the majoritary clade in the sister group here, as compared to the MRCA in Pittis and Gabaldón or Vosseberg, for the inference of the taxonomic affiliation of the donor resulted in a higher specificity regarding the donor. Whereas Pittis and Gabaldón (2016) had a higher proportion of contributions from domains as donors (e.g., Archaea), our current approach provides higher resolution, resulting in a higher proportion of specific clades within that general one (some trees would be annotated as Crenarchaeota and Thaumarchaeota rather than Archaea). We now included this new analysis in the supplementary discussion section entitled "Comparison with previous studies" and show the differences in the Supplementary Figure 7.

In summary, differences between the studies are mainly due to a higher database coverage and a higher taxonomic resolution in our study, and thus the current results should be considered a better proxy for the reconstruction of the LECA-proteome and its origins.

Metabolisms of prokaryotic donors

The authors also inferred metabolisms of potential prokaryotic donors, which represents a weaker part of the paper. First, the metabolism is inferred based on pan-proteomes defined at (super)phylum or lower taxonomic level. E.g. Recent data support that eukaryotes branched from Heimdallarchaeia (Eme et al, 2023), and mitochondria from a sistergroup of all Alphaproteobacteria (Munoz-Gomez et al, 2022). As such, it seems inappropriate to use phylum-level pan-proteomes to infer metabolic properties of the alleged donor lineages. Alos, it is unlikely that present day proteomes are representative for the donor lineages that donated genes some 2 billion years ago. This becomes clear when looking at Figure 3d, where e.g. methane metabolism is inferred for the Asgard ancestor, which is not in line with most recent data, which do not support a methanogenic host (note that hypothesis in the citations mentioned in line 311 are not supported by any data).

Response: *We agree with the reviewer that the metabolic inferences of the potential donors are more indirect and therefore more speculative than other results of our study. Nevertheless, we consider they are a necessary component to provide a framework in which*

to discuss our results in the context of existing models for eukaryogenesis (which are in general fully speculative). Much has been discussed on the potential contributions from Archaea or Alphaproteobacteria to the early eukaryotes, and this has been done since the earlier identification of donors, even when the data on their potential metabolism was very scarce. This is the first time several non-alphaproteobacterial / non-Asgard donors are firmly proposed in the light of phylogenomic data and it is therefore important to explore the implications to the eukaryogenesis models.

Regarding the choice of a phylum-level reconstruction, we consider it to be an appropriate compromise, considering the resolution of taxonomic affiliation at the overall gene tree distribution level. The current specific affiliations are proposed based on concatenated gene alignments, and it is expected that the signal detected from single gene trees - as assessed here - is more dispersed. As discussed in our manuscript, the exact affiliation within Alphaproteobacteria - or as a sister-group to it - of the mitochondrial ancestor is unclear. Our results from the reconstructions of the origin of the LECA proteome, when considering only the Asgard and Alphaproteobacterial origins, show that there is not univocal signal towards Heimdallarchaea and Alphaproteobacteria within the gene families inferred to come from the nuclear ancestor and the mitochondrial ancestor, but rather some of these gene families have sisters in other lineages within Asgard archaea or Gammaproteobacteria. This can be due to i) insufficient phylogenetic signal, ii) insufficient sampling within these clades (especially poignant in Heimdallarchaeota) or iii) gene loss and transfer dynamics undergone in these lineages after their separation from the eukaryotic ancestors. Our metabolic reconstruction reflects this level of resolution, which we think is better captured at a phylum level. For comparison, and to accomodate the reviewer's request, we now include reconstructions at the Heimdallarchaea and Alphaproteobacterial levels, which provide overall equivalent results to those made at the Asgardarchea or Alphaproteobacteria+Gammaproteobacteria levels (see current Figure 4).

We agree that it is unlikely that proteomes of extant prokaryotes can be per se *representative of an ancestor deep in evolutionary time, this is why we limit our search to capture common metabolic features that co-occur in genomes of a given phylum with the set of genes inherited from that phylum in LECA. In a way, our reconstruction represents the core metabolism of organisms of that phylum that share the genes we inferred they donated to LECA* and were potentially part of the ancestral donor species. *Although still a rough approximation, we consider that this approach is useful to generate hypotheses of what could have been the metabolic features of the proposed donors. We now stress this better in the manuscript.*

As for the methane metabolism of Asgard archaea, we agree that the statement that our data "strongly supports" a methanogenic origin was incorrect, and we have rewritten it. We now discourage this inference based on the incompleteness of the pathways.

In summary, we added reconstructions based at the class level or restricted to Heimdallarchaeal and Alphaproteobacterial genomes to provide a sense of the potential parameter-dependency of our inferred metabolism. Furthermore, we have rewritten several sentences to stress that we are not performing an ancestral state reconstruction, but rather we are inferring potential metabolic features based on common co-occurrences in extant

organisms. We stress the uncertainties associated with these inferences and focus on the hypothesis-generation nature of this approach.

Definition of transfer waves

The authors make the assumption that transfers traced back to the same taxonomic group were transferred together. What is this assumption based on? Furthermore, 15 genes seem a rather low number to regard transfers as a wave. How many transfers are in the "waves" for the 8 groups considered? The method used is only available as preprint (Bernabeu et al, bioRxiv 2024.09.30.615760) and seemingly obscures the distribution of stem lengths across the individual gene trees. The authors need to also show these.

Response: *As for the first question, we indeed observe that genes with different taxonomic origins tend to have a similar distribution of stem lengths, called waves, which we interpret as having been transferred in a similar window of time. The underlying assumption is that the mode of the distribution of stem lengths of an evolutionary event behaves as an estimator of the relative time for the event. This is what is demonstrated in (Bernabeu et al, bioRxiv 2024.09.30.615760, which is currently under review), as the mode of the posterior distribution of the stem lengths correlates with the absolute time estimates given by molecular clock techniques. It is important to emphasise that the power of this method relies on the use of distributions, not single values. As for the novelty of the method, we want to note that the mentioned preprint describes a mathematical implementation of a method that had been described before (Pittis and Gabaldón 2016), already used in other publications, and whose adequacy has been already discussed (see, for instance PMID:34097914).*

With regard to 15 being a rather low number, we want to stress a potential confusion: this value is related to the thresholds we set for the selection of donors that are worth exploring, and is not applied for the waves discussed here or any other analysis. The difficulty of working with gene trees and very ancient events results in low sets of genes whose ancestry can be inferred with confidence. That is the reason why we used a very stringent set of thresholds to consider relevant donors beyond Alphaproteobacteria and Asgardarchaeota. We assumed that donors with at least 15 genes transferred to LECA using highly-stringent criteria (a 75% minimum ultrafast bootstrap, 95% minimum taxonomic bootstrap thresholds and a LECA definition with 7 eukaryotic supergroups) is a considerable threshold to assume that the involved clades may have been frequent donors of LECA. This, as expected, reduced considerably the number of trees pointing to the origin of each donor, as it does with the size of the total proteome. We set the threshold in 15 as the next putative donor had a much lower number of genes assigned (7).

However, to calculate the waves we used all genes pointing to that taxonomic affiliation, even with less stringent thresholds. This is because, as rightly noted by the reviewer and shown in our preprint, a lower number of trees hinders the inference of the relative acquisition timing. On average, the number of trees included in each wave ranges from 160 to 260, depending on the donor and the dataset (eTOLDB + LECA criterion). We think the reviewer might have been confused by our wording, and we have rewritten this part in the main text and the methods to clarify this concern. Additionally, we have updated the supplementary material by adding a figure with the raw and normalised distributions accounting for the number of all the trees pointing to a donor and those kept for the acquisition wave (Supplementary Figure 8).

Handling gene duplications

It is unclear how the authors dealt with gene duplications in their analysis. Numerous gene duplications occurred during eukaryogenesis. If not in same OrthoFinder OG, would these duplications be in separate trees with largely the same non-eukaryotic sequences, potentially inflating the number of acquisitions?

***Response**: The reviewer makes a very good point, which prompted us to assess the impact of the scenario mentioned by the reviewer: that of LECA paralogs ending in different Orthofinder OGs. To do so, we introduced an extra step in our pipeline with the goal of identifying and fusing such paralogous OGs together to then analyze the impact on the results. These analyses show that the impact of ancestral duplications before LECA, initially split by our OrthoFinder step, did not change the qualitative results and main conclusions of the paper. As correcting OrthoFinder inference is not the goal of this paper and the variation on the results is negligible, we decided to keep the results without fusing the potentially paralogous OGs. However, we find this issue interesting and we have included a paragraph in the supplementary discussion (see "Pre-LECA duplications not inferred by the orthology calling").*

If in the same OrthoFinder OG, the "LECA" node in the tree would actually be a duplication node, influencing the interpretation of branch lengths.

***Response**: Yes, ancestral duplications influence the interpretation of the branch lengths. As previously reported by Vosseberg et al. (2021) (10.1038/s41559-020-01320-z), when there is a duplication that results in many stem lengths (one per LECA after each duplication event), the shortest stem length is the closest to the stem lengths values from clades without duplications. Although our initial algorithm did not detect these ancestral duplications, we now implemented this feature to take this into account, we thank the reviewer for pointing this out (explained now in the Methods section "Inferring the acquisition waves").*

Minor comments

General: "prokaryotic" used for prokaryotic and viral genomes/sequences; change to "non-eukaryotic" or "prokaryotic and viral".

***Response:** We agree. The manuscript has been modified accordingly.*

Line 40-41. Please note that the taxonomic (re-)classification of Asgard archaea is currently pending, as another proposal for the nomenclature has been published (Tamarit et al, Syst. & Applied Microbiology, 2024). I suggest you cite both these papers, or neither of the two.

***Response:** We agree with the reviewer and we have decided not to cite either of the papers. We used GTDB taxonomy throughout the paper and feel it is not necessary to justify or cite the source of the taxonomic nomenclature.*

Line 60. "significant": because this is not based on a statistical test, use a different adjective.

***Response:** In the revised manuscript "significant" is changed to "major".*

Line 98. This sentence is unclear; why mention KOs here where previous sentence mentions mLECA-OGs? What is the connection?

***Response**: We have modified this sentence to clarify the meaning, sorry for the lack of clarity.*

Line 146-148. How does the number of genes without prokaryotic or viral homologs compare with previous estimates?

***Response**: Vosseberg et al (2021) estimate 2260 out of the total 10233 gene families to be innovations (22%), which is much less than our estimates 38% of innovations (3971 out of 10376 in TODLBA). This can be attributed to our filtering out homologs that have only domains in common between eukaryotes and non-eukaryotic sequences. On the other hand, Hartman and Fedorov (2002) estimated 347 gene families as Eukaryotic Signature Proteins (ESPs). We attribute the increase to an extended sampling of the eukaryotic Tree of Life that allows us to detect proteins of ancient origin that have been overlooked due to their absence in model organisms (so-called jotnarlogs). We now mention the similarity to previous studies in the revised manuscript.*

Line 214-215: “loss or incomplete sampling”; could also be due to phylogenetic artefacts (e.g., LBA).

***Response**: Phylogenetic artefacts due to lack of sufficient phylogenetic signal are always a possibility and we decided to include this possibility in the text. However, our verticality tests suggest that loss of Asgards homologs can explain the levels of Thermoproteota signals observed.*

Line 246: “depending on the dataset”, explain or refer to the corresponding Supplementary Figure.

***Response**: We added a citation to Extended Data Figure 3.*

Line 246-249. “Notably, all identified donor bacterial taxa are common components of microbial mats, and the inferred order of acquisition of Asgard archaea, Planctomycetota and Proteobacteria is reminiscent of the relative depth in which each of these taxa is found in this type of environments”. This statement is rather ad hoc and cannot be generalized: the composition of a single present-day biofilm is not representative of all microbial mats. Furthermore, what evidence exists for the eukaryotic cell having evolved in a biofilm? Why would organisms present in the same biofilm exchange genes

***Response**: We agree with the reviewer that this is speculative. We nevertheless consider it is an interesting hypothesis grounded in some key observations: i) biofilms and microbial mats are known hotspots for HGT and emergence of new genotypes (e.g. PMID: 29914658 and an extensive literature on exchange of antimicrobial genes in biofilms), ii) our reconstructed LECA proteome composition trends agree with the diversity of this sampled microbial mat, and the inferred metabolic capacities of the contributors to the LECA genome could have performed equivalent ecological roles in similar environments and their layers. Thus, although speculative we consider that this hypothesis can be proposed. Given the*

difficulty of the problem at hand and the scarcity of data, many of the hypotheses in our field are rather speculative. The metabolic (and also taxonomic) structure of these microbial mats tends to be preserved, therefore we used the cited microbial mat composition, as it is one of the best characterised so far. We have now rewritten this part to explicitly indicate that this is based on a single mat analysis.

Line 304-306: one example of phagocytosis-like cell engulfment does not make it likely that the inferred Planctomycetota donor was phagocytotic.

Response: *This is similar to above. We are aware that we cannot infer the presence or absence of phagocytosis in the Planctomycetota ancestor. We are merely pointing out that there have been descriptions of members of this phylum to have phacocytosis-like abilities (albeit with alternative mechanisms to eukaryotic phagocytosis, making them more elusive to detection from gene annotation or homology searches) and are pointing out a possibility. Hypothesis generation (that requires further testing) is an important aspect of scientific research and a common part of the discussion of any scientific results. Again, we now stress the hypothetical nature of the implications of such observations.*

Line 311: methanogenesis not particularly strong in figure 3d, and not consistent with Asgard metabolism. Why is WL not inferred in Asgard archaea?

Response: *Clarified above (see "metabolisms of prokaryotic donors" in the response) and in the Supplementary discussion (Assessing our results in the context of different models for the origin of eukaryotes).*

Line 592-594: eTOLDBA contains the most complete proteomes but how were the other sets selected?

Response*: Taxonomic sampling was prioritized above all else, so if a limited number of proteomes existed for a given taxonomic group they were used for all three datasets (hence the overlap between the datasets). When enough proteomes were available for a given taxonomic group then variability was prioritized. We sorted all proteomes by completeness after cleaning and then assigned them in turn to eTOLDBA, eTOLDBB and eTOLDBC. Finally the three model species, Homo sapiens, Saccharomyces cerevisiae and Arabidopsis thaliana were added to all datasets. We added this to the database section in methods.*

Line 613: "(Supplementary figure cutoffs)"?

Response: *We apologize, this is now removed.*

Line 625-627: "Little difference was found (...)"; mention this difference.

Response*: We added this Information.*

Line 657-658: does this mean that only one non-eukaryotic sequence was added in cases of 499 eukaryotic sequences?

Response*: No, as detailed in the methods, when more than 400 eukaryotic sequences were present then 100 sequences were added if present. "When more than 400 eukaryotic sequences were present, up to 100 non-eukaryotic sequences were added." We removed the confusing part of the sentence and provided the details in the methods section.*

Line 669-672: two different sets of substitution models mentioned for the same analysis.

Response*: The reviewer is correct, we apologise for the inconsistency. The correct set of models is -mset DCmut, JTTDCMut, VT, WAG, LG --madd LG4X,LG4M. This has now been corrected and explained in the manuscript.*

Line 724: "Using the definitions table"; which table?

Response: *We are referring to the table that defines each COG. We specify this now in the text and we have added the link to the COG definitions as per the latest release.*

Line 828-829: "set of representative 35 Asgard archaea and 35 Alphaproteobacterial proteomes"; how were these selected? This paragraph describes the analysis presented in figure 2c, but this explanation is also not sufficiently clear.

Response: *We selected, out of the GTDB species representatives, representative sets of Asgardarchaeota and Alphaproteobacteria as follows:*

- *In the case of Asgardarchaeota, we selected three representative proteomes per order attending to maximum CheckM completeness and, in case of ties, minimal CheckM contamination, which resulted in a set of 35 proteomes.*
- *In the case of Alphaproteobacteria, as there were more orders available, we selected a representative proteome per order (maximum CheckM completeness and, in case of ties, minimal CheckM contamination) and then randomly selected 35 orders out of this set so that the number could be comparable to Asgardarchaeota*

This information has now been included in the methods section.

Figures

Figure 2

A: Bars are not proportional to the numbers they represent: not only innovations but also the viruses bar is considerably shorter than archaea (both 10%). Remove innovations from the figure, so the figure is no longer misleading.
B: Three different values are varied for the stringency measure but it is unclear how this relates to all these different categories on the x axis and what determines their order.
C: Unclear what this panel shows (based on figure legend and main text)

Response*: We have taken note of the reviewer's comments and modified figure 2. With respect to A, we have added an x-axis so that the length of the bars is proportional to the percentage of the LECA proteome they represent, as well as a pie chart to display the relative proportion of direct acquisitions, virus-mediated acquisitions, innovations and unknowns. We hope that this way it is more informative. As for B and C, we have moved these plots to the Supplementary Material (Supplementary Figure 5), as we believed they were too methodologic for a main figure and were related to a specific testing of the*

methodology that is better suited as supplementary information. We have now included a scheme about how we check the verticality in Supplementary figure 5 and an extensive explanation in the Supplementary discussion (Rationale of the verticality test).

Figure 3
A: Thermoproteota are not shown.
B: “Archaea” is mentioned where presumably “Asgard archaea” is meant?
C: Rename Mitoancestor and Arcancestor to resp. Alphaproteobacteria and Asgard archaea to be consistent with other figures and the main text and to remain close to the actual results instead of the interpretation; limits x axis not sufficient to show the value of chromatin genes from Nucleocytoviricota.

Response*: In (A), Thermoproteota are not displayed since this archaeal phyla is not one of the considered donors, but rather is considered a secondary signal (for the reasons outlined in the text). For (B), the proportion of relative abundances is taken from Gutiérrez-Preciado et al. (2018), and “Archaea” represents the abundance of Archaea as a domain, not specifically of Asgard archaea. As for (C), which is now Fig 2e, the terms “mitoancestor” and “arcancestor” are not used in this figure as this figure is focused on the different phylogenetic affiliations, not yet grouped into specific donors. However we added a mark to the clades corresponding to the mitoancestor and the arcancestor in this figure. As for the point on the X axis, we have taken note and corrected the plot scale to display the full value.*

Referee #1 (Remarks on code availability)
N.A.

Referee #2 (Remarks to the Author)
The nature of the last eukaryotic common ancestor (LECA) and the set of events that gave rise to it (eukaryogenesis) are topics of fundamental biological importance. The past decades have seen vigorous debate over these topics, centring on competing models for eukaryogenesis; and in a growing appreciation for the widespread nature of microbial symbioses has led to symbiogenetic eukaryogenesis models rising to prominence. These models invoke microbial partners other than the alphaproteobacteria-related endosymbiont that gave rise to mitochondria or the Asgard archaeon-related host. Until now, the lack of clear signal for a prokaryotic group other than alphaproteobacteria in the ancestries of eukaryotic proteins has presented a major weakness for these models.

Here, Bernabeu, Gabaldón and colleagues seek to provide an updated reconstruction of the proteome of the LECA, incorporating a wealth of recent datasets that provide broader taxon sampling than was previously possible. They infer the ancestral proteome of the LECA from three different datasets, incorporating two different degrees of stringency. By reconstructing the phylogenies of these proteins, they pinpoint their likely ancestries, and use the branch lengths to draw inferences about the patterns of frequency of their acquisitions. Finally, they reconstruct the metabolism of potential contributors to the eukaryotic lineage.

The work is **timely and of broad interest,** and the manuscript well written; the authors have **gone to a lot of trouble** to ensure correct ortholog identification and identify patterns of ancestry in their data. However, I have **concerns about the methods** that should be

addressed in order to fully evaluate the manuscript's claims, and which may affect its conclusions. The paper also presents a great deal of data derived from **functional annotations using automated tools**, without taking into account that the databases that these tools rely on are heavily biased toward non-model organisms, and accordingly without carrying out the degree of **careful interpretation and manual curation t**hat this approach requires. As a result, conclusions regarding the LECA proteome and new Eukaryotic Signature Proteins need significant revision and clarification.

Response*: We thank the reviewer for their kind comments and appreciation of our work. We have addressed all raised concerns in the revised manuscript. We have made an effort to improve automated annotations and remove domain-specific annotations, but hope the reviewer understands that careful manual curation of the potential ancestral functions of hundreds of protein families considered in this study is beyond the scope of this type of analysis as it requires time and expertise beyond the reach of a single research group. We hope that our reconstruction will serve as the bases for additional community-based annotations, and we will promote this through our involvement in initiatives such as the Quest for Orthologs consortium (PMID: 39404012) and LECA research communities (PMID: 39585925) .*

Major points

Phylogenetic methods

* I'm a little confused by the following passage (starting line 688): "Finally, a maximum likelihood (ML) tree was reconstructed using IQ-TREE v2.3.4 and parameters -wbtl -B 1000 -m TEST -mset WAG,LG,JTT --madd LG4X,LG4M --mrate ALL. We used ModelFinder to select the fittest substitution model among a subset of the available models in IQ-TREE. We selected the single matrix substitution models DCmut, JTTDCMut, VT, WAG and LG and the mixture models, LG4X and LG4M". In the second line, three models are mentioned that are not included in the command shown in the first line.

Response*: Thanks for noticing, this is indeed a typo. In our code, we have used the longer set of models that the reviewer writes. The final command used to run the trees has been:* `iqtree2 -s {exp_LECA_og}.metalign.trim --prefix iqtree/{exp_LECA_og} -wbtl -B 1000 -T 8 -m TEST --mset DCmut,JTTDCMut,LG,WAG,VT --madd LG4X,LG4M --mrate ALL`*. We have corrected this in the manuscript.*

* Appropriately, the authors do not simply use ModelFinder with default parameters, but include LG4M and LG4X among the models evaluated. However, it seems clear that they do not include any of the C-series (C20, C40 etc.) profile mixture models, even though these incorporate mixtures of at least 20 amino acid site frequency profiles that are better able to model site heterogeneity (see Baños et al. 2024 https://doi.org/10.1093/sysbio/syad063). This is particularly important given the nature of these datasets, covering large time divergences and possibly functional divergence between lineages.

Response*: As the reviewer points out, we did not include the C-series (CXX) models in the model selection. This choice was initially made to save computation power. CXX models provide a better categorisation of the alignment and generally fit better heterogeneous datasets as the ones we use in this study. To assess whether using this model would affect our results, we ran the TOLDBA trees using an updated command, including some CXX*

models in the selection and using -m MFP (equivalent to -m TESTNEW). We completed 96% of the dataset, having problems reconstructing the largest trees in a reasonable time frame. We have found no qualitative changes compared to the previous results, and we performed the downstream analyses using the already computed trees. We included a section in the Supplementary methods explaining the effect of using CXX models and the -m MFP option (see "parameters for tree reconstruction" in the Supplementary and below).

* If they did use the command shown in line 688, they sought to include all rate heterogeneity parameters in the model evaluation by using the --mrate all option. However, the standard model selection, which they used (-m TEST) does not evaluate FreeRate models (e.g. R3-R10. LG4M and LG4X are also FreeRate models, but these were added explicitly, although note that these are not tested together with other parameters such as +I or +F unless listed in the -mset option rather than with -madd). -m TESTNEW would be the appropriate option to allow these to be evaluated as well. See http://www.iqtree.org/doc/Command-Reference for more details. I don't have access to the authors' own raw IQ-TREE output files, but by running some commands myself, I find that the best model recovered when I use -m TESTNEW in most cases includes one of the FreeRate parameters, which is not the case when I only use -m TEST.

***Response**: Indeed, our intention was to include all the rate heterogeneity models (gamma and free rate). However, we were unaware of the change of the -m TEST command since a specific version. The way to run ModelFinder with all the heterogeneity parameters, as the reviewer points out, is currently either -m TESTNEW or -m MFP, according to the manual (http://www.iqtree.org/doc/Substitution-Models). In our run, the free rate model is selected more often than the gamma model. However, when we added this option when also adding the CXX models we did not observe differences in the results (see Supplementary methods section "Parameters for tree reconstruction"). Therefore, we decided to keep working with the current version of the trees inferred with the corrected command shared in the reviewed version of the manuscript.*

* The authors evaluated support for the sister relationship between eukaryotes and their closest sister group using ultrafast bootstrap support values. As far as I can tell, however, they did not optimize UFBoot trees by nearest neighbor interchange (-bnni option), which reduces the risk of overestimating branch supports due to severe model violations (which is likely to occur in at least a subset of trees here due to compositional heterogeneity, heterotachy or both), even when mixture models are used as recommended above, so this option should be used

***Response**: Thanks for the suggestion, to test the effect of the use of the -bnni option we have re-run our tree reconstruction pipeline for the TOLDBA trees using the selected models and the bnni correction, and calculated the non-eukaryotic clades that have an important contribution to LECA using the criteria defined by the stress test (ufboot-bnni >= 75%, 7 supergroups, proportion of the donor in the sister >= 95% and taxonomic bootstrap >= 95%). These results have now been included in the stress test section in the Supplementary material (Stress test for selecting the donor's). The selected list of inferred donors has not been affected, and as such we carried on with the original bootstrap values.*

* Importantly, to evaluate potential donors to the LECA lineage, the authors used an Ultrafast bootstrap support value cut-off of greater than or equal to 75% in their stress tests. According to the developers of the Ultrafast bootstrap method, this is not appropriate, as support values of 95% correspond to a p-value of 0.05 (see Minh et al. 2013 https://doi.org/10.1093/molbev/mst024, http://www.iqtree.org/doc/Frequently-Asked-Questions). Accordingly, 95% ultrafast bootstrap support should be used as a cut-off value, potentially affecting the major conclusions of the paper.

Response*: Statistical support is a typical and controversial point in phylogenetic studies. Rules of thumb are useful approximations but dangerous if not considered in the context of the problem at hand. Based on the study of Minh et al. (see Figure 1 in that paper) UFBootstrap support equal to 75% or higher still corresponds to a probability of the clade to be true of 0.75 or higher (values are close to the diagonal in the support vs probability plots). We considered that, given the difficulty of reconstructing such ancient splits in single gene trees, this was a reasonable level of confidence that the clade is true. We also want to note that we use the support to assess the most likely evolutionary origin of a given LECA family, which can be robust to changes in the evaluated split. This is why we implemented the so-called taxonomic bootstrap. This measures the confidence of a clade (Alphaproteobacteria, Myxococcota…) being sister to the LECA family and does so by inspecting what fraction of the bootstrap trees supports that assignment. The used threshold for the taxonomic bootstrap was 95%, meaning that the taxonomic assignment remains robust despite some sequences moving out or in of the split (which is what penalizes the standard bootstrap). We also want to insist that our thresholds pass the "stress tests" for the bona-fide donors: the ancestors of mitochondria and archaeal components, and that the use of highly stringent thresholds reduce these true signals to very low levels. We now better discuss this rationale in the methods section: "Stress test" for identifying the main non-eukaryotic contributors".*

I realise that some of the changes outlined above are more computationally and time-intensive than the ones that the authors used, but I don't consider them excessive given their importance to the paper's fundamental conclusions.

Response*: We thank the reviewer of all these technical concerns that are important to assess the robustness of our conclusions. We have addressed all of these factors in a subset of the data (TOLDBA) and included the analysis of their impact as a supplementary section. These analyses clearly show that the discussed trends and the conclusions are robust to those parameter choices.*

Other methods/interpretations

* The authors examined the distribution of branch lengths in single-gene phylogenies as a proxy for the timing of HGT, in order to identify waves of such events from a single donor that might correspond to a tighter/more prolonged association:

"To identify such acquisition waves, we computed the normalised stem lengths as in 3 and plotted the distributions of these branch length values with respect to the potential prokaryotic donor. [...] We inferred the acquisition module using the density function, the genes that are around the peak of the distribution are selected as members of the acquisition module. To obtain these genes, we set the threshold to the first minimum after the first maximum or, in its absence, the first inflexion point of the density curve. Those genes that transferred before the threshold (are in the peak) are included in the module. In

contrast, the ones having longer stem lengths are discarded, assuming that they were transferred posteriorly or the values result from heterotachy or ghost lineages".
This method would seem to inevitably lead to the production of a normal distribution of events over time, which makes using it to try to identify such distributions a circular approach. What do the distributions look like when events with longer stem lineages (described as 'anomalous' in the main text) are not discarded? Is a major peak still apparent in each case?

***Response**: Normally, the full distributions look the same, but with longer tails (see Supplementary Figure 8). The shape of the distribution is still skewed, and can be properly modelled by the gamma distribution, not the normal distribution. This characterisation is what we addressed in our preprint (currently under review, 10.1101/2024.09.30.615760).*

Cutting the values after the first peak allows us to focus the discussion and the inference in the most frequent event we infer that an organism related to the clade the trees point out was involved. Most of the distributions had just one peak, except Gammaproteobacteria and Myxococcota in ToLDBA with five supergroups (Supplementary Figure 8)

* I'd also like to see what the distribution looks like for an example or two of less well represented donor groups, for comparison.

***Response**: We added the raw and normalised distributions of the stem lengths for the studied groups using the TOLDBA 5 supergroups dataset (see Supplementary Figure 8). For the less represented groups, we selected the four less represented groups that had at least 30 trees to obtain a reliable estimation of the density.*

* Fig. 2b depicts the number of trees in which eukaryotes are sister to Asgards and Alphaproteobacteria, respectively. However, given that one of the messages of Fig. 2a is that several other groups are presented to an approximately equal degree in the inferred ancestries of LECA proteins, it would be informative to include these lineages in Fig. 2b as well.

***Response**: We may have not explained correctly the purpose of the stress test. Fig 2b (now moved to Supplementary Figure 5) represents the behaviour of the phylogeny-based assignments to the two broadly accepted LECA contributors (Alphaproteobacteria and Asgard archaea) with respect to different levels of filter stringencies. The rationale of this test is to help us choose a set of criteria that maximizes the proportion of these two proven donors, considered as true positives. These parameters are then used in subsequent analysis to select alternative donors. There is a maximum in the following criteria combination: ultrafast-bootstrap >= 75%, taxonomic bootstrap >= 95% (labelled as high), and at least 7 eukaryotic supergroups to define LECA (labelled as high). These thresholds are used to determine which taxonomic groups beyond Asgard archaea and Alphaproteobacteria contributed a non-negligible number of genes and should therefore be considered as putative contributors. We believe that including other taxa in Fig 2b (currently Supplementary Figure 5) would be confusing to the readers. As this stress test belongs to the setting up of the methodology rather than to the results, and may induce confusion, we have moved the figure to the supplementary.*

* 795, “In the case of more than one eukaryotic group with the maximum proportion in the first sister, we [...] calculated its common ancestor” - how was this done? Did the authors consider the branching order of members of different clades in the sister group (i.e., members of a group branching first are more likely to be the true sister group, with the other group represented having subsequently acquired the gene from the same source)

***Response**: We did not consider the specific branching structure for identifying the donors. Though it is an interesting suggestion, our tips are clusters of genes derived from a pangenome at the order taxonomic level. Hindering the possibility of fully relying on the internal branching of the sister group. Therefore, we used only the proportions in the sister, knowing that the pangenome approach results in lower proportions of the most abundant donors. Still this approach returns a clear most abundant clade. In the case of two or more clades being represented equally in the sister, we calculated the taxonomic most recent common ancestor (MRCA) of these clades. As sisters are inferred at the phylum level, the MRCA can be Eukaryota, Bacteria, Archaea or viruses. We have clarified this in the methods section.*

* It’s difficult to tell what exactly will be included in the supplementary data files since these are not currently available, but these should include the list of datasets originally considered (not just those retained for final analyses), and the complete list of representative prokaryotic datasets used, and these should be referenced in the text.

***Response**: We are sorry, we did not share the proper link to the still drafted zenodo repository. The following link will allow the reviewers and editor to access the data of the project: https://zenodo.org/records/13897946?preview=1&token=eyJhbGciOiJIUzUxMiJ9.eyJpZCI6ImMyYWNiMDY4LWEzNTQtNDFlOC1hMTQ4LTNjNjBjNGFhOWRmNSIsImRhdGEiOnt9LCJyYW5kb20iOiJhZDQ5NjQyOTRkMGViZWU3ZjQ0OGYyMTNlMmEwMjIyYiJ9.RTmHhPfEGirPatRDnaJQxMqvqz9xG-ndHU5qzJwricKpmZfRlpZ5hlQd12lnaf7UmOX_vNTO1pL9aEGwOAVdgQ*

As GitHub does not allow blind sharing via link, we created a folder with the code in the same zenodo repository for the reviewers to access. The code GitHub repository will be open when the paper is published: https://github.com/Gabaldonlab/LECAdonors.

Interpretation of functional annotations

* The paper presents the LECA proteome as one of its major conclusions, but it doesn’t seem to me that Figure 1 conveys the most important or surprising findings of this effort for the reader. Though this is not the authors’ fault, the overall pathway figure can’t show enough detail to be useful, and the larger categories shown in it are so broad (“Lipid metabolism”, “metabolism of cofactors and vitamins”) as to not be informative in the context of a LECA proteome reconstruction either. “Defense mechanisms” in Fig. 1b is a category that would be interesting to comment on in more detail, but the paper doesn’t do this. I’d suggest replacing this figure with one summarizing the key features of the LECA (similar to the general idea of Supplementary Table 9).

***Response**: We are equally frustrated with the difficulty of conveying in a single figure the reconstructed LECA proteome. This is a general problem in all previous LECA or LUCA reconstruction efforts, as far as we are aware. In these works relevant features are shown in*

tables or schematic figures focusing on cellular structures. We wanted to focus on the metabolic completeness of our reconstruction, which we think supports a coherent and rather complete reconstruction. As done in a recent LUCA reconstruction (see figure 2 in 10.1038/s41559-024-02461-1), we plot the metabolic map for the inferred consensus proteome. We agree that these plots are cumbersome and difficult to interpret. We aimed to sum up these metabolic features with Supplementary Tables 4 and 6, where completeness of the modules and their relative origin from the main donors is shown. Supplementary Figure 3 is also a summary of the cellular features of LECA. In addition, we have added detailed representations of each KEGG map indicating the steps present in LECA in the zenodo repository that accompanies the paper.

Regarding the breadth of the functional categories, we have focused on general trends, which we think is what can be observed in these deep evolutionary times. Enrichments and proportion at a narrower functional definition may result in bigger artefacts and a poorer reconstruction. Our aim here was to characterise how the proteome of LECA was functionally distributed, and we found that COG categories were suitable to show these trends.

* More seriously, if a LECA proteome is being presented, then the functional work needs more specialized curation/interpretation by collaborators familiar with the specific pathways/cellular functions under discussion (this is too much work to realistically expect the small number of authors here to do well). If taken seriously, this would be enough material for a stand-alone paper, beyond the messages focusing on donor lineages of this one, though it would need to be taken with a large grain of salt, and the annotation effort would need to take into account issues with the LECA frequently only having had one ortholog of multiple named orthologs in databases. It would also need to take into account issues annotating LECA proteins with only a subset of the domains found in orthologs with functional data, thanks to domain gain/shuffling in LECA descendents.

***Response**: We completely agree with the reviewer regarding the needed collaborative effort in order to assess a more complete functional annotation of LECA and any other ancestor. However, this is beyond the scope of this article. Here, the LECA proteome is constructed in order to explore its potential evolutionary origins and infer gene donors beyond alphaproteobacteria and asgard archaea. Our purpose here was to show the general functional trends that we observed and focus on those more relevant for the eukaryogenesis hypotheses. By using our approach, we detected and annotated the gene families present in LECA. We then relied on up-to-date methodologies such as the KEGG database and anvi-o to check the completeness of the reconstruction. We consider that this level of description is sufficient for our purposes and is similar to that presented in previous publications of LECA.*

We acknowledge that the list of families identified are a good starting point to a more detailed characterization of LECA. As the reviewer points out, careful manual annotation of the hundreds of gene families identified in LECA is a daunting task that needs to be performed through a collective effort and a multidisciplinary team of researchers, including experts on the different pathways and cellular structures. We take the reviewer suggestion on board and will lead a community effort in this direction. We are members of a community with interests in LECA reconstruction (PMID: 39585925) and we consider that that community could be a good starting point.

Another concern of the reviewer is how the orthologs are annotated. Here we used the EggNOG approach, by which the more over-represented and frequent KOs were used to annotate an orthogroup. While not perfect, this is a state-of-the-art approach for the level of automated annotations that we need to resort to before the community-driven manual curation can be undertaken.

Below, I'm choosing some examples found more or less at random that jumped out at me, but I'm sure that someone with more background in some of these proteins could find many more examples than I. For instance:

- Supplementary Table 4, 'Inference of the KEGG modules inferred using the KOs of the consensus proteome of LECA', isn't informative as such, without an extra degree of curation. As it stands, it makes sense that rows highlighted in red are considered by the authors not to have likely been present in the LECA, but what about rows not highlighted in red? Is the reader to infer that the LECA may have had photorespiration? What about heparan sulfate degradation (heparan sulfate being a basement membrane component produced by animals and so unlikely to have been around the LECA much)? It could be argued that the figure just represents pathways that could hypothetically be reconstructed from the proteins present in the LECA, rather than inferences about their actual function, but that seems pointless (and misleading to any readers not familiar with these pathways). So I would strongly suggest removing it as it stands. Supplementary Table 10 summarises pathway components arising in different ancestors with a similar degree of unquestioning model animal-centric annotations, and I feel that it too should be removed as being uninformative.

***Response**: The annotation of an ancestor is always difficult, as we are trying to extract features of LECA from annotations of extant proteins. These systems have evolved during 1.5–2.4 Mya, and the incompleteness and divergence may be high. To avoid misleading interpretations as the ones mentioned by the reviewer, we revised these tables using a manual and automatic approach to remove those annotations that are unlikely to have been present in LECA (such as photosynthesis, CAM plant metabolism, etc). We additionally calculated the taxonomic MRCA of each KEGG module (see the section "Functional annotation of the mLECA-OGs" in the Supplementary Methods and the Supplementary Figure 11), and removed those that were specific to a given lineage within eukaryotes (e.g., animals). We also now better emphasize that the annotations are necessarily based on extant functions of (some) members of the family and that caution should be taken when interpreting the ancestral function in LECA (see Supplementary Discussion, section LECA reconstruction, subsection Overall discussion). Despite the limitations, we consider these tables informative and useful for the community, as they show the general reconstruction of current metabolic pathways using genes that we inferred to have been present in LECA.*

- Meanwhile, Supplementary Table 9 summarises the findings, but in such a general way as not to be particularly useful either, as it's difficult to even know when the functional interpretations are likely to be correct. The only descriptor given for 'transcription factors' is "Almost all the complexes are complete." What on earth does this mean? Caspases, including initiator are also extremely unlikely to be present in the LECA – this is most likely misannotation of caspase-related cysteine proteases (metacaspases, paracaspases, orthocaspases) with different cleavage specificities and functions.

Response: *We are sorry for the ambiguity of some of these descriptors. We have now updated this table taking two actions: i) we calculated a degree of completeness based on the number of KOs present in our reconstruction compared to the full set of general eukaryotic KOs for a given feature, and ii) we calculated the relative contribution of each donor to each feature by calculating the percentage of each donor in the origins of the KOs of a given feature. We further added the missing KOs that are general to eukaryotes in each feature, to show the lack of some parts of the current cellular systems. We updated the Supplementary Table 5 and the Supplementary Discussion section LECA reconstruction including this more curated information.*

- Supplementary Table 8 lists several proteins annotated with the names of bacterial proteins among the Eukaryotic Signature Proteins, including hyfF, sprT, fliT, rub, archaeal flagellar protein flaD, cpeA (phycoerythrin alpha chain, a phycobilisome protein…) and so on. Something has clearly gone wrong here with either the ESP identification or the functional annotation. The list also includes animal-specific proteins (such as NF-kappa-B-repressing factor) that happen to have domains that are more broadly found across eukaryotes, which likely explains the misannotation of these proteins.

Response: *This is the result of current biases in annotations, which are mostly based on a few model organisms. As mentioned before, the annotation of ancestral gene families is not trivial. We agree that manual curation would substantially improve the results, but we have already explained that such a community-based effort is beyond the scope of the present article. To alleviate the annotation bias, as explained above, we have now considered the taxonomic breadth of the different KEGG KOs and removed the annotations (left as unannotated) from those KEGG KOs that do not have representatives within eukaryotes in the KEGG database, or KOs that are taxonomically restricted within eukaryotes (e.g. only in animals and plants) to annotate the mLECA-OGs. While further manual curation of each specific family will obviously improve the annotation, we think that this taxonomic-based curation already removes many of the potential problems. The results are now included in Supplementary Table 9.*

* The abbreviations used in Fig. 3 (and Extended Data Fig. 3) should be fully explained in the figure legend(s).

Response: *Thank you for pointing this out. We have now added the explanation of the abbreviation to the legends.*

* The authors haven't actually shown that the interactions that gave rise to the patterns they observe were symbiotic (though they favour this explanation, and present plausible possible scenarios for it). Repeated exchange of plasmids between members found abundantly over different time periods within the same type of environment would be equally plausible. "Diverse ancestries reveal complex interactions during eukaryogenesis" would be more accurate.

Response: *We have removed the word "symbiotic" from the title. We want to emphasize that by "symbiosis" we do not exclusively mean endosymbiosis, or strict patterns of dependency between the lineages. We here adopt the broader definition of symbiosis (*living

together*) including persistent interactions that can be either mutualistic or parasitic (e.g. as defined in Douglas, Angela (2010), The Symbiotic Habit, New Jersey: Princeton University Press). Repeated exchange of plasmids between lineages, as the reviewer has pointed out, could indeed lead to the pattern seen in the data, but we suggest the high number of transfers and the fact that they were retained reflect persistent interactions that are suggestive of a link beyond mere co-occurrence. We suggest that these gene transfers are footprints of a tight coexistence that can be indicative of metabolic interactions, regardless of the mechanical basis of such interaction. We now specify the basis for this consideration and open the possibility to alternative explanations.*

Minor points

It would be really helpful in the future to number the lines in the supplementary methods/discussion as well as in the main text

***Response**: We now provide numbered lines in the main text and supplementary.*

Figure 2b shows shows the relative contributions of Alphaproteobacteria and Asgardarchaeota through different stringency parameters, i.e. different Ultrafast bootstrap support values for the branc connecting the LECA node with its prokaryotic sister group. These values should be shown on the axis for ease of reading (similarly, Supplementary Figure 6).

***Response**: We have moved these plots from the main text to the Supplementary (Now supplementary Figure 5), as we considered they were more a methodological step rather than a result. We have now indicated the thresholds used in each variable to calculate the Alpha- and Gammaproteobacteria proportions in the x-axis.*

Figure 3b depicts alpha+gammaproteobacteria with a single colour, the same one used to depict only alphaproteobacteria in Figure 3a; it would be helpful to differentiate between these colours (if there is no way to differentiate between the two proteobacterial phyla at different depths; or to clarify in the legend if both alpha- and gammaproteobacteria independently display the same relative abundance curves over depth)

***Response**: We have now used a different palette to indicate the abundances of Archaea and Alphaproteobacteria and Gammaproteobacteria in the figure 3b. As observed in Figure 1 in 10.1038/s41559-018-0683-3, Alphaproteobacteria and Gammaproteobacteria follow similar trends in their abundances through depth.*

Figure 4 shows a violin plot, but no statistical analyses that would allow the authors to say ‘significantly’ here. Visually the Alphaproteobacteria and Nucleocytoviricota look as though they could be significantly different, but without statistical tests this can’t be confirmed.

***Response:** This is a good point. We now have performed a Kruskal-Wallis test to assess whether Pythia difficulty scores were significantly different by donor (p-value < 2.2e-16). Once proven statistically significant, we performed one-tailed Wilcoxon rank tests to check whether any secondary donor had a significantly greater difficulty than either Alphaproteobacteria or Asgardarchaeota (applying Bonferroni correction). We only found that alignments resulting in Alphaproteobacteria affiliations are significantly more difficult*

than for those resulting in other affiliations. This suggests that the trends observed in the main donors should not be artefactual due to alignment difficulty. This new test is included in the revised version of the manuscript.

39, "a genome merger" - I understand where the authors are coming from, but I'm not a fan of this term as EGT isn't fundamentally different from any other kind of repeated LGT from a single donor lineage. I'd replace this with "a symbiotic relationship with extensive gene transfer"

Response: *We agree with the reviewer and have changed to the suggested wording.*

59, "which we purged from low-quality sequences" should read "from which we purged low-quality sequences"

Response: *Thanks, this is now corrected.*

191-193, "We found no significant differences in terms of the amount of phylogenetic signal among sets of trees with different taxonomic assignments" - the referenced Supplementary
678 (and Supplementary Materials), "monophyletic clade": clades are by definition monophyletic, so this should just be 'clade'

Response*: Thanks, all the references to "monophyletic clades" have been corrected to monophyletic groups.*

306, 369 and Supplementary Methods (p.13) have a few instances of a typo, 'Plantomycetota' instead of 'Planctomycetota'

Response*: This typo has been corrected.*

681, "farthest to": should be "farthest from"

Response*: Corrected.*

Supplementary Methods (p.2), "we [...] grouped them into another artificial supergroup that we named TRASH just for labelling purposes in our analysis" - I appreciate this name and hope that it catches on

Response*: Thanks!, we hope so too.*

In the Supplementary Methods (p.2), it seems strange to choose to base the position of the root of eukaryotes in part on Torruella et al. 2024 (bioRxiv) without also mentioning and citing Williamson et al. 2024 https://doi.org/10.1101/2024.09.04.611237. This preprint also supports the split between Stem 1 and Stem 2 that the authors have chosen.

Response*: We have added the Williamson et al. reference, which has meanwhile come out in Nature.*

Referee #2 (Remarks on code availability)
The code was not available (Error: 404)

Response*: The reviewer is right. As previously pointed out, neither the code and data were properly shared in the previous version of the manuscript. We apologize for this mistake that was unintentional, we apparently mixed up the sharing link to the Zenodo repository, and GitHub does not allow sharing still private code repositories via anonymous link. We now have added the correct Zenodo link (https://zenodo.org/records/13897946?preview=1&token=eyJhbGciOiJIUzUxMiJ9.eyJpZCI6ImMyYWNiMDY4LWEzNTQtNDFlOC1hMTQ4LTNjNjBjNGFhOWRmNSIsImRhdGEiOnt9LCJyYW5kb20iOiJhZDQ5NjQyOTRkMGViZWU3ZjQ0OGYyMTNlMmEwMjIyYiJ9.RTmHhPfEGirPatRDnaJQxMqvqz9xG-ndHU5qzJwricKpmZfRlpZ5hlQd12lnaf7UmOX_vNTO1pL9aEGwOAVdgQ) and include a compressed folder containing the code with the same structure as in the GitHub.*

Referee #3 (Remarks to the Author):
This is a **very thorough study** of the gene/protein donors to the last eukaryotic common ancestor (LECA). Using a combination of previous approaches (stem length analysis like in Pittis and Gabaldon 2016 Nature) and newer contributions (e.g., production of a new LECA proteome prediction), the authors carry out a **broad suite of analyses** concluding that eukaryogenesis involved “multiple waves of horizontal gene transfer”, some of which may have predated the evolution of proto-mitochondria from alphaproteobacteria. They also posit that Nucleocytoviricota facilitated eukaryogenesis.

The logic behind the dataset assembly, curation and analysis is **generally sound,** and I commend the authors for their **rigor and attention to detail on many levels.** I like how the paper discusses the fit of their results with various hypotheses for eukaryogenesis and the potential partners / donors involved. At the same time, however, I think the symbiosis emphasis in the title and abstract is oversold. I would say that their paper indicate that the data are complicated, and on balance, the multiple waves model presented is still consistent with several eukaryogenesis scenarios. So one can **question how far this investigation pushes the boundaries of knowledge.**

Response*: We thank the reviewer for the constructive and generally positive comments. We believe the issues around the term “symbiosis” stem from a misconception, as we do not correlate symbioses strictly with endosymbioses. Nevertheless we now have removed the word symbiosis from the title, and framed the use of this term elsewhere in the manuscript.*

As we have pointed out to other reviewers, we suggest that the pattern of gene transfers that we see from the gene trees are footprints of a tight coexistence that can be indicative of metabolic interactions, regardless of the mechanical basis of such interaction. Our aim is not to reformulate a new model for eukaryogenesis, but rather put data forward that can guide the community into more complex scenarios than those that just imply an Asgard archaeon and an alpha-proteobacterial endosymbiont. Our work therefore pushes the boundaries of our knowledge in several ways: First, we provide an updated reconstruction of LECA using the most comprehensive genomic data available. Second, although the presence of genes of different bacterial origins had been previously proposed, we here precisely identified several major donors and make an effort to infer their potential features, and the relative timing of their contributions to LECA, discussing these results in the context of existing

models, and, third we provide compelling evidence of a potential role of viruses as mediators of these gene transfers (which we have expanded on and clarified in this version of the manuscript). This has been previously overlooked, and can help integrate the patterns of transfer donors within a mechanistic framework.

There is also the question of the **robustness of the stem length / branch length** approach taken by Gabaldon and colleagues in the past. -Line 269: “We used these branch length distributions to refine the set of proteins likely acquired from the same donor in a single wave by removing protein families with anomalous branch lengths (see Methods)”. I know that a lot of thought and care has gone into ruling out biases, normalizing the data etc. etc. But I still think that proteins coming from the same donor in a single wave could have very different branch lengths for molecular/cell biological /evolutionary reasons, in which case how can we be sure that proteins with the same branch lengths come from the same wave? I can’t actually tell whether the authors have refined their methodologies in this area in this paper compared to their earlier strong contribution (2016).

Response: *The use of the stem length ratio method for the relative timing of events in eukaryogenesis was introduced in Pittis and Gabaldón 2016 and further tested and developed with a dataset of mammalian gene trees (Bernabeu et al, bioRxiv 2024.09.30.615760). We do not ignore that branch lengths are influenced by diverse factors, but these are unlikely to result in patterns in which branch lengths correlate with the phylogenetic origin of the protein family rather than with their structural properties or their molecular or cellular function (see new test and Supplementary Figure 9).*

Of note, the issue of potential biases of this methodology has been largely explored by us and others. See, for instance section 1 (testing functional biases) and 5 (Control for other biases) of the Supplementary Material of (Pittis and Gabaldón 2016), where it is shown that differences in stem lengths are not resulting from functional differences, changes in subcellular localization, or changes in mutational rates in specific species. Further tests and an analysis of an Ascomycota dataset showing that branch lengths peak at values corresponding to the expected species divergence is shown in a preprint (https://doi.org/10.1101/064873). In our latest preprint, currently under review (Bernabeu et al, bioRxiv 2024.09.30.615760), the method is further improved and tested on a dated tree, and, despite the large variability in factors potentially shaping branch lengths, the results clearly show that distribution peaks, as inferred in the current analysis, accurately correspond to their expected relative position in terms of time of divergence. Hence, since the first publication of the methodology in 2016 this has been refined and further validated. Despite much initial criticism, our approach and previous results have survived rigorous scrutiny by other authors and the overall limitations of the framework have been extensively discussed (PMID: 34097914). In the absence of a clear alternative hypothesis that explains the structure of the observed differences in branch lengths depending on the phylogenetic affiliation of the protein family (and not depending on other factors), the simple assumption that these protein families diverged from their ancestors at different times remains the most plausible explanation. We hope the reviewer considers this a valid working hypothesis, which is not in contradiction with the fact that branch lengths and evolutionary rates are influenced by many other factors. Additionally, we have tested it in our datasets as well, we have now included a test that shows that distributions of branch lengths of different functional classes tend to overlap (see Supplementary Figure 9).

I have a number of comments about the figures, which are summarized under specific comments below. Before that I will address what is for me the biggest sticking point with the results as presented and interpreted in this paper, i.e., the inferred contribution of viruses to eukaryogenesis. Everything that we know about viruses in general and large ddDNA viruses in particular tells us that they do not evolve in remotely the same way as cellular life. Despite the original debate about the significance of Mimivirus, and then Pandoravirus, etc. (e.g., exceptionally large genomes and the presence of a handful of 'cellular genes' (translation factors etc), I can't see any evidence indicating that they have a core genome that could be considered stable enough for an analysis of this sort. There is every reason to assume that the genomes of ddDNA viruses around at the time of eukaryogenesis would have been every bit as dynamic as extant viruses.

A few examples of text to contextualize my point:
-Line 283. "The viral contribution to LECA is enriched in signalling and cell cycle control functions, which are complemented with innovations."
-Line 342: "Functional analysis of viral-transferred genes indicated over-abundance of genes related to signal transduction mechanisms, mainly kinases, and post-translational modification proteins."

These enrichments / over-abundances appear to be robust in the author's analysis, but research also shows that signaling and cell cycle control are functions that giant viruses have acquired from their hosts, where they can play a role in host manipulation. There are many examples of this, including in viruses that today infect eukaryotes but also bacteriophage like the cyanophage that manipulate photosynthesis of their hosts. This is the nature of host-virus interactions today, and I think we can be confident that it was no different in LECA times.

Response*: We agree with the points expressed by the reviewer, and consider we may have not expressed ourselves with sufficient clarity. Our main point is that viruses acted as mediators of gene transfers rather than the original source of genes. For all the reasons pointed out by the reviewer, the most plausible scenario is that the genes that came in the LECA lineage through viruses were in turn acquired by them from other hosts either prokaryotes - as some that we describe here- or other (now extinct) proto-eukaryotic lineages. We now have rewritten several parts of the text to convey this message better. In addition, we have made several changes in the analyses of trees containing viral sequences to reduce the risk of including cases of eukaryote-to-virus transfers.*

In the revised version of the manuscript we have made an additional effort to remove potentially dubious families. Firstly, we assessed that LECA gene families that only have a few viral sequences as a sister-group cannot be properly rooted, and they could indeed represent eukaryotic-to-virus transfers. We therefore classified them as "unknown origin" given this difficulty. When both viral and prokaryotic sequences are present, two scenarios are likely, prokaryotes-to-virus-to-eukaryotes or the other way around. To consider only those cases where the directionality to eukaryotes is more clear, we assessed the taxonomic representation of prokaryotic sequences and, when this was restricted to less than 3 non-eukaryotic orders or fewer than 5 sequences, we considered the origin of this family as unknown. Cases with broader taxonomic representations in prokaryotes are less

likely to originate from eukaryotes and we considered them as viral-mediated gene transfers. We think these additional filters increase our confidence on the directionality of the transfers and on the role of viruses as mediators of gene transfers from bacteria and potentially other proto-eukaryotic lineages potentially existing along the FECA-to-LECA lineage.

- Line 337 – "We explored trees that showed Nucleocytoviricota as the direct sister to LECA and that were in the same acquisition wave. 226 out of the 505 genes (45%) had a bacterial sister that preceded the divergence between LECA and the viral taxonomic groups, suggesting the DNA viruses may have mediated gene transfer into the LECA lineage." This is interesting, but what about the other 55%? What were their sister lineages in those cases? Archaeal? A mix of sequences from archaea and proper eukaryotes?

***Response**: We apologise for the confusion. The "bacterial" in that sentence was a typo and was meant to be prokaryotic. The 55% remaining trees are those that only have viruses as donors to LECA. We have now updated the methodology (see response above) so that these trees are now classified as "unknown" given the uncertainty surrounding the directionality of the transfer.*

The viral data examined in this paper have passed certain bioinformatic criteria and thresholds for inclusion / interpretation. I've looked closely at their methods and Supplemental Discussion, and while the authors touch on the issue of directionality, they don't directly address it, as Irwin et al. did in 2022, where it was inferred that euk-to-virus transfers are much more abundant than the reverse (this paper is cited in the intro, but not in a specific way).

***Response**: We agree with the reviewer and, as explained above, we have addressed this by adding a new analytical step focused on the directionality and including the unknown and virus-mediated acquisition categories. Those trees with only viral homologues and whose transfer directionality cannot be disentangled are now flagged as unknown. Conversely, those trees with a topology ((LECA, viruses), diverse prokaryotes); are considered virus-mediated acquisitions. We also analysed the donors of the viral-mediated transfers in the manuscript (Figure 2c) and discussed this fraction of trees in the new version of the main text.*

So I am questioning whether the same criteria used for analysing prokaryotic and eukaryotic proteins are suitable for folding viral homologs into the broader analysis and interpretation. Can we be reasonably sure that the ddDNA viral pangenome core is stable enough to be included in an analysis like this? If it is, it is not demonstrated in this paper. The fact that papers carrying out deep phylogenetic analyses of viruses struggle to find more than a half-dozen genes that are even shared by all the viral lineages of interest – let alone genes that are suitable for phylogenetic analysis – suggests to me that there is no useful core for a deep level, eukaryogenesis analysis such as this.

***Response**: While it is true that we cannot establish a large core pangenome for viruses, we do not believe that this is necessary for this kind of analysis. In fact the aim of using a pangenome approach for prokaryotes is related to our need to downsample the large amount of prokaryotic sequences present in GTDB and maximise taxonomic diversity given the limited number of sequences we can put in a tree and still use sophisticated*

phylogenetic models. However, in the case of the viruses we used the clustered database (the representative sequence of the clusters using mmseqs2 at 95% identity percentage), not a core pangenome. We think this mitigates the conceptual boundaries of a pangenome in viruses, as pointed out by the reviewer. We have stressed this in the main text.

To be fair, the authors are suitably cautious in some places with respect the role of viruses. For example, “viruses may have mediated gene transfer into the LECA lineage” (above). But in their discussion (line 361) they say: “Our results also underscore a central CONTRIBUTION FROM Nucleocytoviricota, and suggest a potential role of these viruses in mediating some of the gene transfers from the bacterial donors.” In the abstract, they speak of a “facilitating role of Nucleocytoviricota viruses”, and their Supp Discussion starts off: “Our results suggest an important role of viruses during eukaryogenesis.

Response*: We thank the reviewer for appreciating our cautiousness, and agree that some of the wording might leave room for additional interpretations. We have now clarified this in every place where viral contributions are mentioned. We consider viruses acted as mediators of gene transfers, which provides a potential mechanism (in addition to endosymbiosis) for the origin of prokaryotic genes of a variety of origins in LECA. Even if mediating, this is an important role during eukaryogenesis.*

In the end I’m still left wondering what the role of viruses in eukaryogenesis actually was, **beyond gene transfer vehicles** within and between the cellular lineages that were in the mix. Is this very surprising? As long as there have been cells there have been viruses infecting them. From the quotations above, the authors are saying their data speak to viruses as facilitators of gene transfers between cellular life forms during LECA times; **they are not saying that viruses themselves were somehow important players in evolution as discrete biological entities** with stable gene contents and metabolisms that might be relevant to how different types of cells / biological entities came together and gave rise to eukaryotic cells. This tempered conclusion (which I think is appropriate) is in contrast to the sentiments of some of the papers cited, such as Guglielmini et al. 2019, Forterre and Prangishvili 2013, and Forterre and Gaia 2016. This last paper discusses the viral eukaryogenesis theory (and shows a figure illustrating how an engulfed virus might have given rise to the nucleus.). It may be the case that the viral factory gave rise to the protonucleus. If so, given everything we know about the genes extant genes viruses contain and how viruses evolve today, I see no evidence that we can obtain results for or against these hypotheses.

Let me apologize for this rant about viral evolution, but I think it's really important to get right in a paper like this. If over interpreted or misleadingly summarized, there is a danger that it can be picked up on too strongly by others. So to conclude: I would encourage the authors to reconsider how the viruses feature their analyses and whether or not they are confident that the data in hand actually have something meaningful to contribute to the eukaryogenesis question.

Response*: The role of viruses as facilitators of gene acquisitions during eukaryogenesis, has not been, to our knowledge, quantified before. In this work, we demonstrate cases of virus-mediated acquisitions to the LECA protein repertoire, assess their relative contribution in the LECA proteome as compared to direct acquisitions and innovations, and put forward*

the role of viruses (especially Nucleocytoviricota) in mediating gene gain. We believe this is an interesting and novel finding of our work, that can help better contextualize and shed light into some open questions in eukaryogenesis, such as the sparsity and patchiness of the ESPs in Asgard archaea (as we do find viruses mediated the acquisition of genes from the Asgard clade, apart from direct acquisition by vertical inheritance) and the presence of several donors outside the known asgard and proto-mitochondrial partners.

We agree with the reviewer that we do not have sufficient data to either prove or dispute the theories put forward by the aforementioned papers, and in particular the theory on the origin of the nucleus proposed by Gaia 2016. We do think that our work contributes specific data on viral-mediated transfers that can be further explored in relation to the origin of certain structures, but that this work is not the place to reformulate eukaryogenesis. We are happy that the reviewer thinks that our tempered discussion is appropriate. We made a big effort in presenting results emerging from the data and the potential implications for eukaryogenesis, which are more speculative. The comments from this and other reviewers made us realise that a bigger effort in contextualizing the potential contribution of viruses would be beneficial for the manuscript. Therefore, we have modified our assessment of gene trees with viral sequences in a more cautious framework and clarified several passages in the manuscript.

Specific additional comments

-Line 41/42 – cites references 14-16, some of which were published long before the discovery of the second of the two “established partners”, i.e., Asgard archaea. So I would suggest dropping them out. They are not relevant to the specific phylogenomics question at hand.

Response: *This part of the introduction provides an historical perspective and the previous attempts to reconstruct the phylogenetic affiliation and metabolic features of the proto-mitochondrial partner. We think this is relevant for the question at hand, which does a first attempt for the additional potential bacterial partners identified here.*

-Line 49 – archaeal not archeal

Response: *The typo has been corrected, thank you for pointing this out.*

-Line 59 – purged OF low-quality sequences…

Response: *This has been corrected.*

-FLUAs, FLUOs and FLUPs. These acronyms are a bit cumbersome. I would suggest the authors define them again in the legend to Figure 1 so that readers don’t have to go searching for them.

Response: *Good point, we have defined them again in the legend of Figure 1, as suggested.*

-Line 149 – reference for original description of the ESP concept (at least, when it became explicit in that way, presumably the very first Ettema papers on Asgard).

Response*: We have added the original paper by Hartman and Fedorov, 2002 as this is the first definition of an Eukaryotic Signature Protein. We did not include the reference to Ettema's list of ESPs as they are always in relation to their presence in Asgard archaea, and thus they represent a subset of those proteins that could be thought to represent "eukaryotic signature" processes.*

-Line 333 – "Nucleocytoviricota, also referred to as nucleocytoplasmic large DNA viruses, is a phylum of viruses known to infect diverse eukaryotes and to comprise giant viruses with large genomes39." Nucleocytoviricota includes some 'giant' viruses with large genomes but it doesn't comprise them. As the authors are no doubt aware, the consensus now is that giant viral genomes evolved multiple times independently from ancestors with much smaller genomes by gene acquisition, mostly from their hosts (related to comments above).

Response: *The reviewer is right, we have changed "comprise" to "include".*

- Fig 2b/c. What is the black line connected with dots in part b, decreasing from low to high stringency? It is the number of trees, right? (labeled on the right part of the graph). Is there a way to make this more obvious (e.g., "Stress test for the number of trees (black dot connected by line)? As is, there is a lot going on in the figure, and it doesn't help that the color coding for part C is also used for part B (and includes a dark gray (not black) LECA box, which I can't see anywhere on the plots, nor the light gray control, which is I believe the point of these 'negative controls').

Response*: This line was intended to show the thresholds that we used to detect the most prevalent donors beyond Alphaproteobacteria and Asgardarchaeota. We realize that this figure might have been confusing. We have decided to remove these panels and discuss these methodological aspects in the methods sections, and supplementary material (supplementary figure 5). This allowed us to use Figure 2 to focus on results to better explain the origins of the LECA proteome and transferred the methodological discussion of the donor identification to the methods and supplementary discussion.*

Fig 2c. "This is in stark contrast to the observed relationships of LECA genes with bacterial ancestors (Fig. 2c), indicating ancestries that cannot be explained by vertical inheritance through the mitochondrial ancestor." Where are the bacterial signals here, beyond Alphaproteobacteria? Again, is the point that they are not there in Fig 2c? This is important to know, as the conclusions on lines 213-218 mention Thermoproteota (part a, 2%). The language here is very important to get right for the reader to grasp the take-home message.

Response*: We have re-written this whole paragraph to improve clarity. Fig 2c has now been moved to the supplementary, as it relates more to the methodology rather than to the results. We have added an additional panel to the figure to better explain the interpretation of the results and have extended the explanation on the test in the methods section: "Verticality test" and in the Supplementary discussion (Rationale of the verticality test).*

Fig 3a (and b). I'd suggest making the euk, mitoanc., and Asgard colors jump out more in part a relative to the other bacterial groups. Currently Fig 3b is described in the text before Fig. 3a, and it doesn't help that the color coding appears at the very bottom of the figure, under part d (I didn't notice at first). Readers will also be confused by use of

“Thermoproteota” in the main text but not in the figure. What does this term apply to, exactly? Domain Archaea? The former Crenarchaeota? Clarity needed.

Response: The color code is maintained through all the figures, the difference here is that we created a new category for something that joins two existing categories, that is, mitoancestor for the grouping of alpha- and gammaproteobacteria and arcancestor for the grouping of Asgard and Thermoproteota. The color coding is maintained to guide the reader into this grouping. We have, however, decided to change the colour to a lighter one for the mitoancestor and the arcancestor and a darker one for Heimdallarchaeota.

Line 232. “Our results (Fig. 3b, Extended Data Fig. 2) depict distinct branch length distributions peaking at different values, suggesting different relative timings.” As far as I can tell, Fig 2b is simply a schematic representing trends from a microbial mat study published by Gutierrez-Preciato 2018. Did you mean to say Fig. 3a here?

***Response**: The reviewer is right, the correct figure is 3a. We are sorry, and we have updated the main text to reflect the correct citation.*

Fig 3. The term Arcancestor is used here in two important places. What does the term mean, exactly? It’s not used in the main text. Blue refers to Asgard (part a), Archaea (part b), and Arcancestor (parts c and d) all in the same figure. Non-specialists will find it difficult to connect all the dots, in part because the color coding is a tiny afterthought at the very bottom of the figure.

***Response**: We agree with the reviewer that this was not properly explained. We have split the figures (Fig 3 into Fig 3 and Fig 4) in order for the reader to better understand the terms arcancestor and mitoancestor. They are artificial categories derived by the merging of the genomes overlapping the KOs contribution to LECA of Asgardarchaeota and Thermoproteota (for the arcancestor) and Alpha- and Gammaproteobacteria (for the mitoancestor). We merged these categories according to two different reasons in each case: i) we detect many genes from Thermoproteota likely due to the low sampling of Asgard genomes and ii) we cannot affiliate the mitochondria to have evolved from within alphaproteobacteria, so the ancestor may be in between Alphaproteobacteria and Gammaproteobacteria. We now better explain these definitions and their meaning in the text.*

Fig 3c. “Proportion of transferred GENES from the donor

***Response**: The Figure 3 has been extensively reworked, and this has been corrected.*

Fig 3c. Nucleocytoviricota. Chromatin (top line). Why does the line not have a circle? Why does it extend beyond 5 on the horizontal axis?

***Response**: As we modified the assessment of genes with viral homologs (see our response above), this over-representation analysis has changed and the term Chromatin has disappeared. In this case, the chromatin was highly over-represented and axes were cut. This is related to the many observed eukaryote-to-virus transfers in these genes*

(10.1038/s41564-024-01707-9), which are now excluded from the analysis by using the unknown origin category for the mLECA-OGs.

Referee #4 (Remarks to the Author)
In this article, Bernabeu et al. reconstruct the proteome of the last eucaryotic common ancestor (LECA) based on publicly available eucaryotic genomes, functionally annotate it, and then investigate the origins of the LECA proteome using phylogenetics. In particular, they use a methodological approach that points at different potential contributors aside from the Proteobacterial ancestor of the mitochondrion and the Asgard ancestor. Their phylogenetic analyses point at six major contributing lineages: Alpha and Gamma-Proteobacteria, Myxococcota, Planctomycetota, Asgard and Nucleocytoviricota (a lineage of giant viruses). They reconstruct the core genome of potential contributors from the different lineages and establish their "metabolic profile". The authors also revisit from their data the list of the eucaryotic specific proteins (ESP). Overall, this study finds that there is the same level of evidence for other bacterial and viral contributions than the ones generally acknowledged from the Alphaproteobacterial mitochondrion ancestor and the Asgard.

This study builds upon a previous study published in 2016 in Nature by Pittis and Gabaldón where a late acquisition of mitochondrion was postulated, and where the contribution of several other prokaryotic lineages was pointed. Some of the methodology from this previous work was reemployed here ("stem length analysis"). The novelty here resides on a **systematic investigation of the origins of different contributions to the LECA** proteome, which is made possible by the availability and efficient analysis of datasets **covering much more of the biodiversity** than the ones employed in similar studies before. It also comes from the assessment of several acquisition waves by LECA, from different donor lineages.

In general, I found the article **very well written,** and the **dataset gathered seems very solid** considered available genomic data for both procaryotes and eucaryotes. I found the **conclusions drawn well balanced and supported** by the data. The topic is of **exceptional interest** to the readership of Nature and in a context of a highly debated process of eucaryogenesis. However, I had some methodological concerns and questions that I would like to discuss. Moreover, I believe that the manuscript would benefit from clarifying further in the text what truly constitutes novel findings compared to those from the Pittis and Gabaldón paper from 2016.

Response: *We thank the reviewer for the positive and constructive comments, and the appreciation of the interest of the results and the rigor in the analyses. As you will see, we have clarified the methodological concerns and we have made more clear the novel findings uncovered by the present work.*

Major comments
- My main concern comes from the use of the **stem length analysis**. To me it is unclear how this length can really relate to the timing of acquisition. Once acquired, a gene can endure different selective pressures that could temporarily affect its evolutionary rate before it is fixed, and I don't see why this force should be constant and homogeneous among different gene families, as it would depend on the function of the gene acquired and the genetic

background of the receiving ancestral organism. Could the authors elaborate on this, and explain why they think selective pressures would not affect the stem lengths or would be consistent across the different waves of acquisition? How could this impact the relative timing of the waves measured? Have the authors tested for instance if the stem length varies between different functional categories? Could this be disentangled from having more or less ancient donors from a same lineage?

***Response**: We acknowledge that the methodology may have been received by some skepticism, as the one pointed by this reviewer. However, we reiterate here the same arguments we discussed in our response to reviewer 3: The use of the stem length ratio method for the relative timing of events in eukaryogenesis was introduced in Pittis and Gabaldón 2016 and further tested and developed with a dataset of mammalian gene trees (Bernabeu et al, bioRxiv 2024.09.30.615760). We do not ignore that branch lengths are influenced by diverse factors, but these are unlikely to result in patterns in which branch lengths correlate with the phylogenetic origin of the protein family rather than with their structural properties or their molecular or cellular function. Of note, the issue of potential biases of this methodology has been largely explored by us and others. See, for instance section 1 (testing functional biases) and 5 (Control for other biases) of the Supplementary Material of (Pittis and Gabaldón 2016), where it is shown that differences in stem lengths are not resulting from functional differences, changes in subcellular localization, or changes in mutational rates in specific species. Further tests and an analysis of an Ascomycota dataset showing that, despite huge diversity in functions and protein structure, branch lengths peak at values corresponding to the expected species divergence is shown in a preprint (https://doi.org/10.1101/064873). In our latest preprint, currently under review (Bernabeu et al, bioRxiv 2024.09.30.615760), the method is further improved and tested on a dated tree, and, despite the large variability in factors potentially shaping branch lengths, the results clearly show that distribution peaks, as inferred in the current analysis, accurately correspond to their expected relative position in terms of time of divergence. Hence, since the first publication of the methodology in 2016 this has been refined and further validated. Despite much initial criticism, our approach and previous results have survived rigorous scrutiny by other authors and the overall limitations of the framework have been extensively discussed (PMID: 34097914). In the absence of a clear alternative hypothesis that explains the structure of the observed differences in branch lengths depending on the phylogenetic affiliation of the protein family (and not depending on other factors), the simple assumption that these protein families diverged from their ancestors at different times remains the most plausible explanation. We hope the reviewer considers this a valid working hypothesis, which is not in contradiction with the fact that branch lengths and evolutionary rates are influenced by many other factors. We nevertheless have now included a new control (Supplementary Figure 9) that shows that when analyzing transferred proteins by functional properties and not by taxonomic assignment, no pattern emerges.*

- If one wants to infer that a protein was ancestrally found, one needs to show that the protein phylogeny follows the species phylogeny, indicative of vertical inheritance. Regarding the phylogenetic analyses of the origins of the LECA proteome, it seems that there is no real “sanity check” of the eucaryotic phylogeny in the eucaryotic part of the tree. Could the authors elaborate on the reasons why they think that a protein was vertically transmitted from LECA to contemporary organisms?

Response*: We want to make an important consideration here: verticality across the whole eukaryotic species tree is not relevant for this study as we are interested only in the pre-LECA origins of a family and not how it evolved across eukaryotes after its acquisition. Eukaryotic HGT could confound our analysis only at the level of distinguishing a genuine LECA node in contrast to a gene with a similar widespread distribution across supergroups due to HGT among these groups. Considering the suggestion of the reviewer, we assessed the topology of the eukaryotic clade to evaluate the possibility of the LECA node being the result of such inter-eukaryotic HGT. For this, we scanned the first split of the LECA node and assessed the last common ancestor of each of the two resulting sub-clades. If we speculate that the inferred LECA node is the result of an HGT event between eukaryotic supergroups, meaning this event happened after LECA had already diverged, then we would expect that neither of the sub-clades would map very deeply in the eukaryotic species tree. Instead, if one of the two sub-clades maps to the root of eukaryotes, marked here by the presence of species from Stem 1 and Stem2 in the same sub-clade (now supported by Williamson et al. 2025), then a potential HGT event would have happened before LECA and, therefore, it does not affect our inference regarding the origin of that gene family, nor the qualification of that family as being present in LECA. Based on this reasoning, we analyzed the mLECA trees in the TOLDBA dataset and found that in 96% of those trees, at least one of the splits mapped to the root of Eukaryotes. As the LECA node is often classified as a duplication, we scanned the mLECA tree for the first eukaryotic speciation node and repeated the analysis for that node. The results did not change much, with 94% of the trees having at least one split mapping to the Eukaryotic root. Therefore, the selected LECA nodes are unlikely to be the result of an HGT event between supergroups after LECA resulting in the species distribution we use to detect it. This sanity check is now mentioned in the section "Reconstruction of the ancestral eukaryotic proteome" of the main text and the methodology is expanded on in the methods (section Monophyletic LECA orthogroups – mLECA-OG–).*

- I do not fully understand the rationale of looking at sisterhood of LECA-inferred proteins in phylogenetic trees to find donors, and not try to find also "LECA-inferred" proteins within a monophyletic clade of bacteria (as opposed to sister to), as a proof for bacterium-to-eucaryotes transfer. Usually, "received" genes descending from an ancestral transfer form a clade nest within the (broader) donating lineage. How often was this found? Does the proposed method also consider those cases? How can this work in a context where the root of the gene trees is unknown? Otherwise, do the authors consider that the transfers are so ancient that the respective last common ancestors of contemporary prokaryotic lineages are all younger than the ancestral donating procaryote?

Response*: We agree with the reviewer that having LECA nested within a specific monophyletic prokaryotic clade rather than as a sister-clade would be ideal in a standard analysis. However, there are some considerations that need to be taken into account. First of all, our phylogenetic trees are built using homologs selected from a taxonomic representative database of prokaryotic pangenomes and clustered viral proteins, meaning that our trees limit the depth of each clade. As a result, many sister-to relationships could in fact be nested-within if the full spread of sequences of that clade would be used. Thus the consideration of sister-to relationships is in part a necessity of the streamlined database use. Secondly, as the reviewer indicates, our transfers are ancient (precede the origin of LECA) and the sister-clades are the closest living relatives of the ancient donors to LECA, the true*

donor could easily be an extinct or unsampled lineage, in which case we would not expect LECA to fall within a given taxonomic group. HGT between prokaryotes will also have had an impact on the clarity of the trees and as such we prefer to use the more relaxed approach of only searching for the sister group.

- What was the rationale behind the artificial rooting of the gene trees (e.g. line 680-681)? This was already discussed in Supplementary Discussion. Could the authors explain briefly in the main text how the root picked could influence the assignment of the LECA proteome origins, or how the controls made help ensuring the methodology is robust?

***Response**: We have now added this information in the main text and we detail the choice of rooting method in the Supplementary methods section "Rooting method". The additional filtering we have set in place to classify trees with few non-eukaryotic sequences as unknown ensures that the rooting method as described in the supplementary methods will not affect the definition of LECA and its sister clade.*

- Regarding the contribution of viruses (but this also apply to procaryotic contributions), how to make sure of the directionality of the transfers? Or what points toward a virus-to-eucaryote rather than the converse? This could be clarified/discussed in the text.

***Response**: As we have mentioned to other reviewers that raised this same point, we have now created a new donor category named Unknown where we place all such trees that have a limited number and small taxonomic diversity of sister sequences, for virus and prokaryotic contributions. This ensures that all those LECA groups for which we establish a donor would be difficult to explain based on the assumption of a wrong rooting method.*

- This work points at several waves of transfers to the ancestor of LECA, yet no specific case is directly shown using phylogenetic trees. I think it would be important to convince of the credibility of the methodology, to display and discuss several cases of modules transferred, and how it reflects in phylogenetic trees. It could be from some of the cases further discussed in the Supplementary Discussion?

***Response:** Discussing two or three protein families in detail would disproportionately focus the reader on some cherry-picked examples. The literature has extensive examples of detailed analysis of specific families (see, for instance, PMID: 39849086, 40033103, 38472392, 35697693, 32747565, among others). At those levels, one can curate better the data and inspect the phylogeny in a manual fashion, but the overall picture is lost. This study is a large-scale study, where we analyzed pervasive trends among 60,000 trees analyzed automatically. This is not a study focused on a few families. Both types of study are complementary and important and we do not want to give the wrong impression of what type of study this one is. In the paper we cite some specific studies showcasing families with the same ancestries as the major donors identified here, which we feel provides independent support to our findings.*

Minor comments

- Some of the interpretations would require revising the text to make it more justified and clearer. For instance on lines 215-216, it is unclear why the authors consider that

Gammaproteobacteria signals may actually be Alphaproteobacterial. And on lines 214-215, why Thermoproteota signals are interpreted as Asgards'.

Response: *We first considered that gene loss or weak signal mapping to sister clades of true donors could be a more parsimonious explanation of the many donors observed rather than considering each signal a different donor. We next performed a test on a set of Asgard archaeal and Alphaproteobacterial gene trees to assess their behavior within our pipeline in absence of their closest sister (i.e Thermoproteota and Alphaproteobacteria) and saw that these results actually fit our data (detailed in the Methods and Supplementary figure 5). We have thoroughly revisited this section of the manuscript to improve readability of the text.*

- I think it would be great for the non-specialist readership that is not aware of the recent literature to instill more clearly what constitutes truly a novelty here when compared to previous work, in particular what is a new hypothesis or constitute new results, and what previous hypotheses are challenged by this work. To me, this is not enough emphasized as is.

Response: *We now stress better the novel findings of our work.*

- It is said on line 607-608 that one genome per genus from the GTDB was used and the ones with maximal completeness/minimal contamination selected. Could the authors please indicate the range of genome quality for the MAGs used in the end? Was a threshold of genome quality applied?

Response: *We selected one genome per genus from the GTDB species representatives, which already pass the GTDB's criteria for being selected as representatives (see 'Quality of GTDB representative genomes' on their release 214 statistics). The median CheckM completeness is 94.50% (range 18.13-100, mean 89.53) and the median contamination is 0.990% (range 0-46.77, mean 1.492). We now indicate this in the manuscript.*

- For clarity, could the authors display on Sup Fig 1 the number of genomes available for the different eucaryotic lineages?

Response: *We have added the information on the number of genomes per lineage in eTOLDB to the corresponding Supplementary figure*

- On a same note, could a Venn diagram representing the different eTOLDB datasets added to the sup data? This would help to apprehend the overlap of the A, B, C datasets.

Response: *We have added to the Supplementary Figure 1 an upset plot including this information, indicating the weight of each clade in each intersection, as we feel is more informative.*

- Lines 738-739, maybe cite the supplementary tables?

Response: *Given the extension of this table we have not included it in a supplementary table rather it can be found in the zenodo repository (https://zenodo.org/records/13897946?preview=1&token=eyJhbGciOiJIUzUxMiJ9.eyJpZCI6ImMyYWNiMDY4LWEzNTQtNDFlOC1*

hMTQ4LTNjNjBjNGFhOWRmNSIsImRhdGEiOnt9LCJyYW5kb20iOiJhZDQ5NjQyOTRkMGViZWU3ZjQ0OGYyMTNlMmEwMjIyYiJ9.RTmHhPfEGirPatRDnaJQxMqvqz9xG-ndHU5qzJwricKpmZfRlpZ5hlQd12lnaf7UmOX_vNTO1pL9aEGwOAVdgQ) *within the mLECA-OGs folder. We now omit the word "table" in the text to avoid confusion.*

- Sentence on lines 389-842: please rephrase to make it clearer.

Response: *The sentence covering 839-842 has been removed from this part of the text and moved to the methods' subsection "3.2. "Stress test" for identifying the main non-eukaryotic contributors", where it corresponds. To provide some details on this part, we have included a new section in the Supplementary Discussion about this: "Selection of putative donors using the 'stress test'".*

- Figure 3D, please increase the size of the legend for taxonomical groups at the bottom.

Response: *This figure has been re-worked. The legend corresponds to Figure 3A-B, which is now Figure 3. This has allowed us to move the legend and make it more legible.*

- Ext data fig 4: would have been great to indicate more clearly the donating lineage to LECA.

Response: *Extended Data Figure 4 is a reconstruction of the prevalence of different metabolic modules amongst the genomes from each donor lineage that most resemble the protein footprint left by the gene acquisitions by LECA. In this sense, all of them are donating lineages to LECA. We do observe certain trends, such as the informational/metabolic archaeal/bacterial divide, but as we mention throughout the paper, the LECA proteome is a mosaic and metabolic pathways are often a mixture of acquisitions from different donors and innovations. As such, we cannot say that a given pathway was entirely donated to LECA by a given lineage. In any case, to help readers understand this, we have created a new supplementary figure (Extended Data Figure 5) of the metabolic modules in LECA which follows the same structure as Extended Data Figure 4 but depicts the percentage of KOs from each origin among the major donors.*

Referee #5 (Remarks to the Author)
A SUMMARY: The manuscript reports a reconstruction of the last eukaryotic common ancestor's proteome, and traced back the most likely phylogenetic origin of each protein family. They show evidence for several waves of horizontal gene transfer from diverse bacterial and viral donors some of which apparently prior to the mitochondrial endosymbiosis.

B ORIGINALITY & SIGNIFICANCE: This manuscript makes a **considerable contribution** to understanding the evolutionary origin of eukaryotes. Using the latest genomics data, it provides an **updated perspective** on eukaryogenesis, reinforcing existing models of serial symbiotic interactions with a diverse array of bacterial, archaeal, and viral taxa. It also offers more precise insights into the relative contributions of prokaryotic donors and their taxonomic affiliations than previous studies.

C-F METHODOLOGY, STATISTICS, UNCERTAINTIES &:The methodology appears **robust,** and the conclusions seem **well-founded**, often discussing alternative interpretations.

G REFERENCES: The manuscript relies heavily on **self-citations.** More credit should be given to previous work by others (see below).

H CLARITY & CONTEXT:neome Abstract and introduction are clearly presented. In certain places, the results and conclusions are not sufficiently clear (see below).

Response: *We thank the reviewer for the positive comments and the appreciation of our work. Some paragraphs that were unclear have been extensively worked upon, and we have improved the figures as well to make them more easily understandable. We have polished the text to clarify some key concepts, and we have expanded citations where we considered appropriate.*

DETAILED COMMENTS AND SUGGESTIONS:
Line 19-20 Throughout this section and the entire manuscript, prioritize citing early foundational papers on the topics instead of relying heavily on self-citations.

Response: *We have added some citations to other foundational papers. We have extensively worked in this area and we have kept citations where relevant, even if they included some of us as authors.*

20 Numerous papers highlight the involvement of other bacterial partners and viruses in eukaryogenesis. Clarify that the debate is not about their involvement but about the specific type of cellular organisms involved.

Response: *We have clarified this issue*

48-51 Rephrase the sentence to improve clarity.

Response: *We have re-written this sentence*

115 To improve readability, the figure legends should spell out abbreviations.

Response: *We have spelled out the abbreviations that we felt could be misleading, we have kept abbreviations used extensively in the text*

120 The Suppl Info file should discuss the sometimes considerable difference between the frequencies in LECA and FLUPs (e.g. signal transduction).

Response: *As we mention in the main text, differences between LECA COG category distribution and those of extant free-living phagotrophs (FLUPs), are likely due to post-LECA expansion of the protein repertoire for these functions and probably reflect lineage-specific adaptations. This is especially true for signal transduction, and, to a lesser extent, cytoskeleton and cell cycle, which bodes well with our depiction of LECA as a rather complex organism, but lacking finer regulatory and signaling machinery that is present on*

extant eukaryotes. Our aim with this figure is to assess our LECA reconstruction against the backdrop of extant unicellular organisms, and characterizing the innovations and expansions that drove the increase in certain COG categories is beyond the scope of this paper. Nevertheless, we added a section in the supplementary (see "LECA reconstruction") commenting on this issue.

159 Instead of a textual explanation, depict graphically the size of the Innovation rectangle. Also, indicate and graphically represent the affiliation of the remaining bacteria-like (23%), archaea-like (4%) and virus-like (4%) proteins (e.g., as unresolved).
Indicte the actual nr. of proteins within the rectangles of 'Acquisition', 'Innovation', 'Bacteria', Archaea', and 'Viruses'.

Response: *We have extensively modified this figure. Now, a pie chart displays the proportions of the type of gene families. Additionally, we have now added a category ("unknown") for those whose origin cannot be ascertained. Moreover, we show percentage rather than number of proteins, as these numbers are the consensus of the contributions of these clades through all the tested datasets, we could include them in case of specific datasets (as it is shown in the Supplementary Figure 4). We hope that, with this, the figure is more easily understandable.*

162 The description of fig.2b should be clarified. Explain the y axis and the black dots.

Response: *We have modified this figure, which has now been moved to the supplementary (Supplementary Figure 5) we included the x-axis and explained the dashed line.*

169 Legend 2c would benefit from a more detailed and clearer explanation.
The term 'verticality test' needs to be defined.

Response: *We have now marked with the title "Verticality test" the section of the methods giving the definition and the details of this test. The explanation has also been extended in the main text to ensure it is clearer. Moreover, we included a scheme in the Supplementary Figure 5 (where this figure is currently placed) describing the rationale behind this test and a more in depth explanation in the Supplementary discussion "Rationale of the verticality test".*

175-195 This paragraph, along with the unclear legend of Figure 2b, is difficult to follow.

Response: *We have rewritten extensively this paragraph to improve its readability. Figure 2b has been additionally moved to the Supplementary and reworked to make it more understandable.*

197-220 Again, this paragraph, along with the unclear legend of Figure 2b, is difficult to follow. Also consider exploring alternative explanations for the findings.

Response: *As mentioned in the comment above, we have improved the wording of this section of the text to improve its readability.*

202 Give examples of the closest sister groups.

Response: *we have added this information*

205 This conclusion needs more explanation.

Response: *This paragraph has been re-written. The explanation behind the verticality test is, additionally, in the Supplementary Methods.*

223-287 This text does not fit under the header 'metabolism'. One could introduce a separate section with its own header.

Response*: We have changed the title to "Relative timing and potential metabolic nature of gene donors", which better reflects the content of this section.*

232 Fig. 3a (not 3b).

Response: *Thank you for pointing to this typo, we have now corrected it.*

237 The 'longer lengths in Thermoproteota' is not obvious from Fig.3a.

Response*: We added a supplementary figure (Supplementary Figure 8) with the branch length distributions that show the Thermoproteota distribution, whose empirical mode is considerably larger.*

252 Legend lacks essential details and lacks clarity ('of the mode'?; 'mitoanc';)
Specify the dataset to which the shwon distribution ordering corresponds, and the datasets for which it is different. Otherwise, the figure is misleading.
The color scheme of taxa is too small, barely readable.
Thermoproteota, mentioned in the text, are not referred to in the figure.
Explain abbreviations in d:('Aut', 'Het').

Response: *Figure 3 has been split into several figures now. As for "posterior distribution of the mode" we are referring to the mathematical mode, that is, the most frequent value (the "peak" in the distributions) . This has been clarified. The term "mitoancestor" as well as other abbreviations in Figure 3d (now Figure 4) have also been explained in the text. We further worked on the current Figure 4, to clarify the legends and caption. Now, the colour legend is embedded in the axis, and all the acronyms are properly defined in the figure caption. Regarding the citation of Thermoproteota in the text, this corresponds to the current Figure 3, in which we now include the information for this clade, as we want to emphasise the functional contribution of each detected ancestry.*.

278 Cite literature or mention earlier findings indicating that functions acquired from the one or other taxon are enriched in core or metabolic functions.

Response: *We have re-written the paragraph and the information related to trends in functional categories across donor lineages has been moved elsewhere in the text. We have added additional literature citations.*

351-389 The discussion largely repeats ideas already discussed in the Results section.

Response: *We feel the need to stress the implications of the main findings, which requires some re-statements of what those findings are. We have worked on the discussion to minimize repetitions and streamline it, while keeping clarity.*

365 The manuscript reports evidence that eukaryogenesis involved more partnes than Asgard ancestor and an alphaprotobacteria, a view that has been brough forward earlier and by other researchers. Therefore, for clarity and fairness, it should be stated which of the current models of eukaryogenesis the presented data fit (instead of stating which they do NOT fit).

Response: *We do not believe a single existing model fits all of our observations, but we now highlight also some aspects of some models that are in line with the findings. Our aim here is also to constructively discuss some of the aspects of the existing models that could be reconsidered in the context of our findings.*

376-391 This paragraph would benefit from more conciseness, and elimination of ideas mentioned in the results section.

Response: *We have re-written this paragraph which is now more concise.*

393-398 Repeats ideas discussed in various places earlier in the manuscript.

Response: *This paragraph has also been re-written to improve conciseness*

Response to Reviewers comments (our responses indicated in red color).

Referee #1 (Remarks to the Author):
The authors made a good effort to address and clarify the concerns and comments raised by the reviewers, and as a result the robustness of the analyses/results and clarity of the manuscript has improved significantly. Still, the following comments are insufficiently addressed to warrant publication:

Response: *We thank reviewer 1 for the appreciation of our efforts towards providing more clarification and robustness to our results, and we agree that the manuscript has improved thanks to their comments. We now have addressed the remaining points.*

1. Metabolisms of prokaryotic donors
- The authors maintain that the metabolism of the selected taxa that contain the donated genes is reminiscent of the ancient donor. This remains a weak point in the manuscript (despite the context provided in the response letter). The limitations of the approach employed by the authors should be explicitly mentioned in the text, indicating that more appropriate methods exist to infer ancient metabolic properties (ancestral state reconstruction).

Response: *We now explicitly mention in the text that our approach is different from that of ancestral state reconstruction. We have considered this option but concluded that we do not have enough resolution on the bacterial species tree representing all the genomes used nor the exact phylogenetic placement of the ancestor in the tree - we approximate at the phylum level but we do not want to reconstruct the ancestor of e.g. all alphaproteobacteria. Although ancestral reconstruction methods might be considered conceptually more appropriate, these sophisticated methods require appropriate models and sufficiently resolved phylogenetic context, which we consider not to be in place thereby leading to unreliable results.*

Our chosen approach relies on the two lines of evidence where we consider we have high certainty: i) the taxonomic affiliation of the extant genomes and 2) the phylum-level taxonomic affiliation of the origin of LECA genes. Using these robust data, our approach approximates some functions that were likely to be present in an unknown ancestor within that phylum based on the pathways that commonly co-occur in that phylum with the ones that an ancestor of that phylum transferred to LECA. The assumption is then that pathways that co-occur in that phylum today also co-occurred back in the time when the genes were transferred to LECA. We think this is a reasonable assumption, and we hope the reviewer agrees with the usefulness of that approach, despite its associated uncertainties. We now clearly acknowledge in the manuscript that this approach is a proxy rather than an accurate reconstruction of the ancestral donor's metabolism; we now stress this limitation more in our article. We also think that providing the results in terms of prevalence of the pathway on extant descendants sharing transferred functions, gives a sense that this is not a certain assessment but rather an assessment of the likelihood that a pathway was present.

- To illustrate the point above: Assuming most genomes encode at least 50% of inferred donated genes, wouldn't this analysis bias the inferred metabolisms to Lokiarchaeia (most genomes/GTDB genera) for the Arcancestor and Rhizobiales for the Alphaproteobacteria? Please clarify.

Response: *We agree with the reviewer that the reconstruction might be affected by the composition of the Databases, but this is true for any reconstruction method based on extant sequence data (including ancestral state reconstruction). We have opted to select one representative per genus (following a criterion of maximum completeness/minimum contamination) to alleviate the relative weight of better-sampled clades within GTDB. However, we agree that there are still imbalances, so we now acknowledge that limitation in the methods section and provide information on the relative representation of each class for the relevant donor clades (Newly added Supplementary **Figure 12**).*

*In addition, to assess how the over-representation of some classes may influence our reconstructions, we performed a re-sampling with replacement strategy. For this, we first selected one representative per genus, and kept the genomes sharing at least 50% of the genes transferred from that group to LECA in its acquisition wave, and annotated the genomes. We then subsampled genomes, such that the classes within the phyla (or order, for the mitochondrial ancestor) were equally represented (100/n genomes per class, rounded to the closest integer, n being the number of classes). This process was carried out 100 times, and we calculated the prevalences of the KOs and modules across all iterations. The results we obtained were very similar and are now on the newly added **Supplementary Figure 13**.*

- Furthermore, it is unclear which genomes were used for the inference of the metabolism of prok donors ("donor-like genomes" not found in Zenodo data, numbers also not mentioned, only 162 for the arcancestor in the supplementary discussion). Please provide this data.

Response: *The data was already provided in the Zenodo repository under donor_reconstruction/donor_genomes.tsv. The README file may have not been explicit enough. Moreover, we include the number of genomes used for each donor in the "Robustness of the donor metabolic feature inference" section in the Supplementary Methods. We have now updated it to explicitly state that the file contains the genomes we use to build the donor reconstruction. We are sorry for the confusion.*

- Stating "metabolic potentials of the major donors" in the abstract is a too strong statement in light of the limitation of the approach used by the authors (above). Please tone down or remove.

Response: *We have toned down this to "plausible traits of the major donors".*

2. Speculation about models for eukaryogenesis
- The authors should not confuse 'genetic contributions' with 'stable interactions'. This should be adapted/toned down in the discussion section, in particular the added section in lines 381-388. Genetic contributions? Yes certainly! Stable interactions? Symbiotic interactions could explain this, but alternative scenarios could also account for the observed patterns. It is inappropriate to discredit eukaryogenesis models that only include 2 partners (archaeal host + bacterial endosymbiont) based on the provided evidence.

Response: *We agree with the reviewer that 'genetic contributions' does not necessarily indicate symbiosis. We, however, want to remark that we used symbiosis in a very general*

sense, which includes many different types of possible interactions. In addition, given the many genes acquired by each of the donors, similar to those detected to be acquired from the mitochondrial ancestor, we believe this amount of transfers and their subsequent integration in the pre-LECA host strongly indicate some form of stable interaction. These interactions could have been diverse, from syntrophy, specific predation, or even parasitism, but not a one-off event unrelated to any form of interaction. We hope the reviewers see our point. We have nevertheless revised the whole paragraph to not give the wrong impression that we are "discrediting" prior eukaryogenesis models, we just argue that they could better accommodate our observation by considering additional interactions (in whatever form).

- Regarding alleged Planctomycetota interaction: the explicit mention of phagocytosis in regard of eukaryogenesis models should be toned down given the limited distribution within planctomycetes (1 genus) and lack of knowledge about which genes are involved in this process. Why not simply mention that Planctomycetota and Myxococcota as potential symbionts instead, without inferring endosymbiosis for which this analysis does not provide any evidence?

Response: *We have rephrased this part. We already mentioned Planctomycetota and Myxococcota as potential partners earlier in the text. We now stress that phagocytosis in Planctomyetota has been observed in a single genus, and explicitly mention that our approach cannot provide evidence on host or endosymbiotic nature of the partners. Nevertheless, we consider it useful to discuss how our results could influence other models. In particular, Plantomycetota has the same properties invoked for the host proposed by the syntrophy hypothesis and here we identify a conspicuous signal in LECA for this group and an earlier origin than Myxococcota. This does not prove whatsoever the hypothesis, but it is important to state that, if Myxococcota is considered a plausible host by some models, Planctomycetota could as well be an alternative to consider, given the findings here. We hope the reviewer agrees with us that the current tone is appropriate.*

Referee #2 (Remarks to the Author):
I thank the authors for providing the code and extra supplementary materials through the updated Zenodo link, and for their responses to my concerns. However, several of my major concerns remain, and while I appreciate the authors' efforts to address them, I still feel that these do not go far enough to produce results that are sufficiently robust to support the claims made.

Response: *We thank the reviewer's appreciation of our efforts to address their concern. We have carefully considered all the remaining concerns and have addressed them. We hope the reviewer agrees with our modifications.*

Major points
1)Data availability and referencing

I believe something's gone wrong with the supplementary material file names, information or both. I wanted to look up TATA element modulatory factor (shown as a eukaryotic innovation, K20286 which is correct), and found the annotation listed as:

mLECA_OGs/annotation/TOLDBA_LECA_KO.tsv:OG0009484|LECA_1 K20286 14 14.0 1.0 1.0
mLECA_OGs/annotation/TOLDBA_LECA_KO.tsv:OG0019899|LECA_1 K20286 7 7.0 1.0 1.0
mLECA_OGs/annotation/TOLDBB_LECA_KO.tsv:OG0005880|LECA_1 K20286 25 25.0 1.0 1.0
mLECA_OGs/annotation/TOLDBC_LECA_KO.tsv:OG0007360|LECA_1 K20286 18 18.0 1.0 1.0

But then for corresponding alignment files, using

ls mLECA_OGs/alignments/TOLDB*/*/OG0009484*

I find only mLECA_OGs/alignments/TOLDBC/Folder_6/OG0009484_1.final.metalign , and for

ls mLECA_OGs/alignments/TOLDB*/*/OG0019899*
, nothing.
When I check mLECA_OGs/alignments/TOLDBC/Folder_6/OG0009484_1.final.metalign, that seems to contain sequences of bacterial carboxylases.

For LECA_OGs instead of mLECA_OGs, it's similar. I chose another example at random, VPS8 (K20178), and in the alignment files for this found what seem to be bacterial Condensation domain proteins (OG0021610) and Argininosuccinate synthase (OG0002568), respectively. So this seems to be generally a more widespread issue with the supplementary materials; I hope it will turn out to be a simple naming problem with an easy fix, but the authors should check to ensure that it hasn't affected parts of their overall conclusions. (The Readme file also says that the annotation files contain information on the donor lineage for the mLECA_OGs, I didn't see that.)

Response: *We are sorry for the problems the reviewer may have encountered when searching the supplementary information. This is a large-scale project and we provide a huge amount of supplementary data to allow reproducibility and make our study useful for the community. We checked the problems mentioned by the reviewer. The problems result from us missing to upload the alignments of the "innovations" that come from the first tree reconstructing round, that is, the gene families that in the first search do not have any non-eukaryotic homologs. We did not upload these alignments as no downstream analysis was performed on them. When the reviewer searches for the OG he/she is using a general command that searches in all the TOLDBs (ls mLECA_OGs/alignments/TOLDB*/), this, coupled with the missing alignments, means they obtain an alignment of the same OG tag but in a different TOLDB. As OGs in different TOLDBs are not equivalent, as they are named after independent runs of OrthoFinder, the function is of course not congruent. We apologize about this issue, which results from the amount and complexity of the data shared. To solve this, we have now added the missing alignments to the repository and created a summary file that contains the relationship between OGs and all the files associated with them so that it is easier to track.*

The authors should more often directly reference supplementary tables and supplementary files that provide data underlying each of their claims - including those claims for which main or supplementary figures only are currently referenced. This should be done consistently throughout the main and supplementary texts.

Response: *We have now revised the whole text to make sure the relevant data supporting each claim is referred to.*

The authors mention testing different parameters for phylogenetic reconstruction, and finding little difference between these results and their previous results, but the raw data underlying these finds don't seem to be available in the zenodo repository.

Response: *This was done on a subset of the data. As we explain below, we have now recomputed all data including the complex models and the topology search algorithm suggested by the reviewer. We now base the discussions on the results from this most comprehensive computations. As the reviewer will see, the main conclusions of the previous version of the manuscript remain. The use of more complex models for the tree inference has resulted in trees with overall better support and has allowed us to be more stringent as compared to our previous version of the manuscript. This has had the side effect of losing one of the donors (Chloroflexota), but we believe that stricter thresholds are preferable as they increase the confidence in our results and provide more robust conclusions. The remaining donors have a larger number of trees that pass our thresholds and they do so across all three tested datasets unlike before where we only required they pass the threshold in one database, therefore we have gained increased confidence on our reconstruction. We think the reviewer suggestion has considerably improved the reliability of our results and are therefore thankful for this suggestion. We discuss this in more detail below.*

2)Concerns about the reconstructed LECA proteome

The authors' draft manuscript, as currently presented, would have two major groups of findings: a) those relating to the donors of different groups of eukaryotic genes; and b) a reconstruction of the LECA proteome, including an updated set of Eukaryotic Signature Proteins.

To a concern about annotation of the LECA proteome that pertains to the quality of b), the authors respond that "We [...] hope the reviewer understands that careful manual curation of the potential ancestral functions of hundreds of protein families considered in this study is beyond the scope of this type of analysis as it requires time and expertise beyond the reach of a single research group"

I completely understand and sympathise with this; indeed, my original review included acknowledgement of this ("if a LECA proteome is being presented, then the functional work needs more specialized curation/interpretation by collaborators familiar with the specific pathways/cellular functions under discussion (this is too much work to realistically expect the small number of authors here to do well)"). I fully acknowledge the amount of work that has clearly gone into this section - on top of all of the other work in the paper! Nor can any LECA proteome reconstruction be perfect. However, any work claiming to be an updated LECA

proteome published in Nature will be used as a reference by Nature's broad readership (especially as it is absolutely not trivial for the readership to double-check the results from this manuscript, given the amount of data to wade through), and so it must be reasonably reliable and robust. At this time, EggNOG's approach, while still the best automated tool available, fails for the many gene families in which correct orthology assignment is difficult because domain shuffling has taken place, or where lower than average levels of sequence homology are present. These include gene families with major implications for the functional potential of the LECA, such as those involved in membrane deformation, adhesion or signaling. Until better tools are available, manual curation that employs expert knowledge of the underlying biology, with careful presentation of the results in a manner that is informative to both specialist and non-specialist audiences, is therefore still essential (which as the authors agree is indeed beyond the reach of a single research group).

This also extends to discussion of the topic: currently, this section in the supplementary materials lacks almost any citations of or discussion of previous work on the origins and evolution of these pathways. This is inadequate scholarship, and doesn't allow the readers to compare the authors' results with what might be expected from earlier work. The vague wording also makes it difficult to double-check the results from the authors' own analyses, or even to understand what is meant in some cases. Supplementary Tables 4 and 5 are referenced, but these don't mention gene names, so that when the authors discuss (for instance) "We inferred a LECA that also had a basic cell adhesion mechanism" or "Our LECA model contains most of the genes that are predicted as shared between Human and the algae C. reinhardtii and were obtained through a combination of acquisitions and innovations [no citations]" or "Our LECA inference depicts almost complete modern DNA and RNA processing systems (e.g., polymerases, transcription factors, ribosome, etc.) with some specific RNA polymerases missing", with no further detail given in the supplementary tables referenced, their use to the reader is very limited and must be largely taken on faith. The 'signaling and adhesion' section mentions adhesion proteins and quorum sensing proteins - the latter of which is really interesting if true - but I can't check these results as they aren't mentioned anywhere in the supplementary tables, and no pointers to this evidence are given in the text (no supplementary files are referenced, no gene names or orthogroup numbers are given).

As a related point, Supplementary Table 9 mentions annotations of new putative Eukaryotic Signature Proteins, which the authors define themselves in their table title as "Innovations are those KOs annotated in trees of any database and for which *we do not find any non-eukaryotic homolg* [sic]" This title is immediately contradicted by the table itself, which lists the KO MRCAs for just over 6% of these as 'LUCA', as they are found in Bacteria as well as eukaryotes (these still include photosystem genes, as well as kynurenine 3-monooxygenase, aldolase and other genes that are notoriously not eukaryotic innovations). Clearly there is an issue in the overall annotation process, either with the OG analysis underlying these data, or with the KO MRCA. Since this affects at least 20% of the annotated pathways even after reannotation, it therefore calls into question the paper's conclusions with regard to the ancestral proteomes and their functions overall (of the LECA but also of the prokaryotic donors).

This goes back to my point in my initial review that bacterial proteins were among the supposed ESPs. The authors countered that this was down to "current biases in annotations,

which are mostly based on a few model organisms" - but if the genes under discussion are supposed not to have any non-eukaryotic homologs (by the authors' own definition in the table legend and given their use of the term 'innovation'), then it shouldn't have been possible for these to be misannotated as prokaryotic proteins. It's possible that the table simply mistakenly included non-eukaryotic innovations and that every line with LUCA as the KO MRCA should have been removed; but then the number of ESPs should be updated in the text. I also don't believe that these are the only genes affected: at minimum the ribosomal proteins listed aren't all eukaryotic innovations (RPL28 is in at least Heimdallarchaeota, RPL36 and RPS7 are found in bacteria, RPL29 is an ortholog of bacterial RPL35; hedgehog family proteins arose from a domain fusion along the animal stem lineage, but if any hedgehog domain-containing proteins are being counted in this category, then it makes less sense to classify VPS proteins with domains found in Asgard archaea as eukaryotic innovations...).

Supplementary figure stpf10, the probability of various photosynthetic pathways having been present in the mitoancestor are shown as being higher than those for most amino acid degradation pathways, and on the order of that for F-type ATPase. If this is true, then it's a very interesting finding that should be discussed in more detail; but it's a lot more likely that these are pathway components that carry out non-photosynthetic roles, and are present in this capacity.

The claims presenting these findings as an updated LECA proteome and a new set of ESPs should therefore be removed from the abstract, main text, Figure 1 and supplementary discussion. The supplementary tables can still be included to aid in understanding and interpreting the authors' findings related to the donor lineages (a) in my description above). The authors are then free to use their reconstructions as the basis for subsequent high-impact work through the initiatives that they mention.

Response: *We agree with the reviewer that current automated approaches and databases are limited and can only go to a certain extent in reconstructing the traits of an ancient organism such as LECA. However, we humbly disagree with the idea that such an automated reconstruction should be removed from the paper. The reviewer asks us to remove any description or downstream analysis of the updated LECA proteome and the ESPs from the manuscript. We consider that this solution is not constructive and would not improve the manuscript but rather reduce its value. We understand the reasons of the reviewer to prefer a different approach that is beyond the scope of this manuscript, but we would like to argue in favor of the value of including the reconstructed LECA proteome and the discussed results, while acknowledging its limitations. The reviewer says "any work claiming to be an updated LECA proteome published in Nature will be used as a reference by Nature's broad readership", and we think our backbone reconstruction is a valid reference used in the context of the methodological limitations of the approach - as all previous references discussed below and also future reconstructions, which will also have their own limitations.*

To accommodate the useful views from this reviewer but still present results that are central to the article, we propose a constructive solution, which we think represents a reasonable compromise between our two different perspectives. We also want to stress that the remaining four reviewers had no issue with this part of the manuscript and several of them

validated the manuscript as a whole. In the following paragraphs we argue for our proteome reconstruction as a valid approach providing useful insights, in brief:

1- While acknowledging the limitations, we provide some important examples from the literature where similar approaches have been used to gain insights on ancestral organisms, while no comprehensive reconstruction of the entire LECA uses (as of yet) the ideal approach of manual expert curation suggested by the reviewer. We thus argue that our approach is framed within the state-of-the-art in the field, it is robust, and provides useful insights.

2- We next argue that providing functional inferences, beyond the list of LECA protein families, is a necessary validation that the reconstructed proteome provides reasonable results. Many such inferences corroborate results from earlier studies - many of them from small-scale studies performing manual curation, as proposed by the reviewer. Figure 1 and the supplementary discussion is a means for validation of the reconstruction, in relation to previous findings, rather than a discussion on a newly discovered LECA feature. We now make clear in the manuscript that this is the purpose of this analysis and acknowledge the limitations of an automated methodology.

3- We agree with all the specific points raised by this reviewer on the inconsistencies found, and the lack of proper citation to previous works. We have extensively revised all the supplementary text and material, and we outline all changes performed to accommodate the reviewer's suggestions and corrections. We think this part has now extensively improved over the previous version, and we took special care that what we wrote was not only based on our automated annotations but also validated by previous works, which are now properly cited.

1- On the validity and usefulness of the LECA proteome reconstruction
First of all, the reconstruction of the protein family set that was likely present in LECA is a necessary step before our analysis of putative donors. The reviewer implicitly agrees with this as they understand the tables (protein family lists, i.e. the proteome) should be included in the manuscript. This list in fact is a reconstruction of a LECA proteome, and it is updated from previous reconstructions because the databases are now taxonomically rich. It is thus an updated reconstruction of the LECA proteome.

This reconstruction is admittedly incomplete or subject to errors, but definitely robust and informative even in the absence of expert curation. Similar (but less sophisticated approaches) have previously been used to gain insights on LECA (see below) and it is unclear why we should refrain from doing it here. The argument that more detailed insights could be gained in the future with further efforts could be used to invalidate any scientific progress, and we think our reconstruction is robust and comparable in accuracy and detail to previous efforts supported by reputable and highly-cited publications, including some in this very same journal, Nature.

We consider that our reconstruction is robust, as it is based on three independent TOLDB datasets, and obtained highly similar results with several alternative computational pipelines. Moreover, our reconstruction follows the state-of-the-art in the field. To argue this, we have searched the literature for any LECA reconstruction using "manual curation that employs

expert knowledge of the underlying biology", as suggested by the reviewer. We have found no such study that encompasses the full LECA protein repertoire, only small-scale studies on specific proteins or pathways and review-type compilations, which is understandable given the difficulty and amplitude of this task, as pointed out by the reviewer. This is consistent with the reviewer not pointing to any specific study as the way to go, in their response. Finally, comprehensive reconstructions using automated methods have contributed important insights and have been published in reliable journals, and encompass articles that have been highly cited by the community and used as a departing point for more expert curation in subsequent analyses, underscoring their usefulness. Beyond the studies in which some of us participated (Vosseberg et al. Pittis et. al.) that include similar LECA reconstructions, we have identified several others that support the use of similar approaches to gain insights on ancient organisms or processes, below we list some of those.

Cox, Rachael M., Ophelia Papoulas, Shirlee Shril, Chanjae Lee, Tynan Gardner, Anna M. Battenhouse, Muyoung Lee, et al. 2024. "Ancient Eukaryotic Protein Interactions Illuminate Modern Genetic Traits and Disorders." bioRxivorg. https://doi.org/10.1101/2024.05.26.595818.

Deutekom, Eva S., Julian Vosseberg, Teunis J. P. van Dam, and Berend Snel. 2019. "Measuring the Impact of Gene Prediction on Gene Loss Estimates in Eukaryotes by Quantifying Falsely Inferred Absences." PLoS Computational Biology 15 (8): e1007301.

Dmitrijeva, Marija, Janko Tackmann, João Frederico Matias Rodrigues, Jaime Huerta-Cepas, Luis Pedro Coelho, and Christian von Mering. 2024. "A Global Survey of Prokaryotic Genomes Reveals the Eco-Evolutionary Pressures Driving Horizontal Gene Transfer." Nature Ecology & Evolution 8 (5): 986–98.

Moody, Edmund R. R., Sandra Álvarez-Carretero, Tara A. Mahendrarajah, James W. Clark, Holly C. Betts, Nina Dombrowski, Lénárd L. Szánthó, et al. 2024. "The Nature of the Last Universal Common Ancestor and Its Impact on the Early Earth System." Nature Ecology & Evolution 8 (9): 1654–66.

From this, we argue that, despite being limited and subject to improvement by manual curation, our approach is valid, follows the state-of-the-art, and constitutes a valuable update of the LECA proteome reconstruction.

2- Downstream analysis validate LECA proteome reconstruction by assessing consistency with previous findings

The reviewer suggests removing Figure 1 and all references to the reconstructed LECA proteome in the main text and supplementary, but agrees to provide the reconstruction as supplementary tables. However, we believe this will be detrimental for the article as it would leave the readers with no means to assess the validity or completeness of the reconstructed proteomes. The reviewer says "Any work claiming to be an updated LECA proteome published in Nature will be used as a reference by Nature's broad readership (especially as it is absolutely not trivial for the readership to double-check the results from this manuscript, given the amount of data to wade through), and so it must be reasonably reliable and robust.", but at the same time agrees to provide the reconstructed LECA proteome as supplementary. We believe that providing the reconstructed items simply as a list of items without any downstream analysis or discussion that reinforces the value of the reconstruction would be misleading and would not reflect how complete or consistent this reconstruction is. With this assessment we do not propose any new feature for LECA, but

discuss how complete the reconstruction is and how congruent it is with previous findings. These comparisons and discussion reinforce the completeness and validity of our reconstruction, which we consider central to the message of the article and not the subject of a separate study.

We nevertheless understand the reviewer's concerns. We now express the purpose and value of this reconstruction more carefully by saying that we "approximate" the functional potential of LECA, and clearly state the purpose of these analyses by saying "To assess the completeness and validity of our reconstructed LECA proteome, we assessed the reconstructed functional potentials in terms of its congruence with previous inferences about LECA.". We also acknowledge the limitations and correct all specific issues pointed out by the reviewer (see next section of this response).

3- Acknowledging limitations and reducing the impact of annotations

To make absolutely clear the purpose and limitations of our reconstruction to any readership, and correct all issues mentioned by the reviewer, we did the following:

- *We now use a taxonomic-aware filtering step to automatically curate annotations. With that filtering, metabolic modules that in KEGG are restricted to non-eukaryotic groups or to too narrow eukaryotic groups (e.g. mammals) are removed. In addition we have manually curated a certain number of pathways whose taxonomic distribution was wrongly annotated or artificially restricted in KEGG (e.g. mitochondrial ATPase was restricted to animals in that database). We acknowledge this amount of curation is insufficient to amend all potential database errors, but we consider it has considerably improved the level of annotation, which is now up to a higher standard than previous automated reconstructions. Moreover, these new filterings correct most of the cases mentioned by the reviewer.*

- *We start the relevant LECA reconstruction section in the supplementary with a subsection entitled "Limitations of automated reconstructions" where we mention the limitations pointed out by the reviewer and make clear the purpose of these analyses.*

- *We explicitly mention in the main text that we reconstructed the LECA proteome "using an automated approach similar to previous studies" to avoid unrealistic expectations from readers and set the level of our reconstruction to similar preceding efforts.*

- *We agree with the reviewer that our supplementary discussion was not rich in citations to previous work, and we have amended this by extensively citing previous studies and re-writing the whole text. As a result, the number of citations in the supplementary text has almost doubled.*

- *From some of the comments of this reviewer, we realize that we did not properly explain our use of KO annotations as a proxy for functional potential of the LECA OGs. We must note that our OGs and KEGG KOs do not have a one-to-one correspondence, as KEGG has a different scope and approach -focused on broad functions across the tree of life- than ours -focused on the origin of LECA families-. This explains some of the cases highlighted by the reviewer in which an OG is an*

"innovation" but belongs to a KO that is distributed beyond eukaryotes. This may be due to a shared domain between two otherwise unrelated OGs that KO considers as the same functional entity. We now clearly specify this by saying "Note that there is not an exact correspondence between KOs, which we use to approximate functional annotations to OGs, and the OGs reconstructed here. This means that an OG may be restricted to eukaryotes but its functional annotation is described by a KO that has a broader distribution, perhaps due to domain sharing with other OGs. This also means that our functional annotations are broad and may not specifically describe the diversity of specific functions within extant eukaryotes, or the ancestral function in LECA. Despite these limitations, KO-based annotations provide a useful mean to explore the metabolic completeness and the overall cellular features likely present in LECA"

- *Similarly, some of the issues mentioned by the reviewer such as the presence of homology beyond eukaryotes for some of the families annotated as "putative innovations" are inherent to the complexity of protein family evolution and the intrinsic problems of homology and orthology definitions. Any threshold for homology detection will inevitably result in false positives and false negatives and different approaches will set the balance between false positives and negatives differently. We acknowledge that some of the protein families recognized as "putative innovations" by our approach may share domains or functional annotations with prokaryotic proteins. These likely correspond to protein families that have a domain reshuffling or sequence divergence in eukaryotes sufficiently large to be discarded by our homology filters. This is why we used the word "putative". We now stress this even more by saying "Note that such putative innovations may include some protein families with protein domains or KOs annotations present in prokaryotes, as domain reshuffling or extensive sequence divergence may result in failure of homology detection by our procedures".*

- *We have rewritten the text to avoid any vague wording and now we are more explicit, indicating the number or fraction of proteins detected for that specific pathway or process.*

- *Now the last columns of table 4 include the KO names that are missing or included in the pathway so that the reader can identify the gene family we are referring to. We have also updated the annotation files in the zenodo repository to include protein codes, you can find them in (data/mLECA_OGs/annotations/).*

- *As suggested, we have removed references to quorum sensing or adhesion proteins, they were removed from the above mentioned filters.*

- *As suggested, we now discuss that our inference lends some support for a potentially photosynthetic mitochondrial ancestor.*

- *We also point out that the data we provide in the zenodo repository can be used in conjunction with the KEGG database to obtain an overview of which pathways are included in our LECA reconstruction. Indeed we also provide the KEGG images*

coloured according to our reconstruction. This can be a useful tool to better explore manually our LECA proteome from a metabolic point of view.

In summary, we have rewritten all the discussion of potential LECA traits, focusing on congruence with previous studies, which are now extensively cited. We have also shortened this section to not include families or traits that were found to be unclear by the reviewer. We now explicitly discuss the limitations of the automated procedures and anchor the definitions used here to the underlying thresholds and methodology used.

3)Phylogenetic methods

Supplementary methods, 'Parameters for tree reconstruction': if I'm understanding the authors right, they used the command shown in the box (iqtree2 -s {exp_LECA_og}.metalign.trim -T 8 --mset DCmut,JTTDCMut,LG,WAG,VT,LG+C10,LG+C20,LG+C30,LG+C60,LG4X,LG4M -m MFP -B 1000 --prefix iqtree/{exp_LECA_og}) to assess the effects of including mixture models, but then chose to go ahead with trees computed without the C-series mixture models in the interests of time. It would be helpful for the reader to include the final command that was used for these trees. Was it iqtree2 -s {exp_LECA_og}.metalign.trim -T 8 --mset DCmut,JTTDCMut,LG,WAG,VT, LG4X,LG4M -m MFP -B 1000 --prefix iqtree/{exp_LECA_og})? The line "we decided to keep working with the current version of the trees inferred with the corrected command shared in the reviewed version of the manuscript" makes me unsure whether this was the case, or whether they used the trees originally computed for the non-revised manuscript.

More seriously, "The use of this option when using the stringent parameters used to select the donors (ufboot-bnni >= 75%, 7 supergroups, proportion of the donor in the sister >= 95% and taxonomic bootstrap >= 95%) caused a drop in the number of trees assigned to each donor. This drop was irregular between the sister groups ranging from 30% in Myxococcota and Nucleocytoviricota to 70% in Chloroflexota and Gammaproteobacteria. Despite this, and accounting for the overall reduction in the number of trees that pass the filters, the selected taxonomic groups remained stable.": the selected taxonomic groups remained stable, but the number of trees that pass the filters overall do not, and nor do the proportion of sister groups chosen. This is not something that the authors should simply be ignoring. This will affect the major conclusions (e.g. those shown in Fig. 2, Fig. 4; and since it affects the number of trees that pass filters overall, it is likely to also affect branch length distributions and therefore Fig. 3; Fig. 1 may also be affected, but for this, see my comments below). The authors should therefore use -bnni for all of the trees that are used to support their final conclusions. This is more computationally intensive, but appropriate given the need for robustly supported conclusions.

In their response to my comment on C-series mixture models, the authors state that "we did not observe differences in the results" between a set of trees produced in their initial analysis versus a set of trees computed with the option of these models. This is true only for the trees that finished running. This is an important distinction: I would expect simpler, less parameter-rich models to perform as well as mixture models for many proteins, but to be more prone to artifactual results, including artifically high support for incorrect topologies, in some cases. Those cases include particularly fast-evolving proteins, or datasets with some

fast-evolving taxa (for instance, viral sequences), and I would expect the C-series models to run for longer in just such cases. This affects 4% of the phylogenies that the authors double-checked; how many of these included viral sequences? Primarily presenting results generated using a methodology that's less robust to artifacts over this time scale is in any case not very robust, but at minimum, the authors should mention whether the proportion of the dataset that could not be assessed in this way had any overrepresentation in terms of Nucleocytoviricota presence (Nucleocytoviricota are notably not mentioned in Supplementary Tables 11 and 12), or function.

Response: *In our previous revision we used a subset of the data to show that the use of more complex models did not significantly affect the downstream results. Given these results and considering arguments of reproducibility (large computational resources for running such complex models at such scale are not broadly available), and green computing (molecular phylogenetics can have very large carbon emission footprint (https://doi.org/10.1093/molbev/msac043), we opted not to re-do all the analyses. As the reviewer is not satisfied with this solution and insists on using the most sophisticated models available, we have now recomputed everything using these complex models in the model selection step and the -bnni ultrafast bootstrap optimization. We must note that we could only do this in a few months thanks to our privileged access to the Mare Nostrum 5 supercomputer, one of the most powerful in Europe. The overall results remain very similar, despite some numeric variations and the fall of one of the previously considered putative donors below the stricter thresholds we now apply. However, we do agree that several indicators suggest the more complex models were a better choice. Overall the trees are better supported and we have more trees (genes) that passed our strict thresholds, this expands our reconstructions and increases our confidence. Despite the struggle of having to redo the whole study again, we thank the reviewer for this suggestion as it has overall improved the support to our results and has made our manuscript methodologically stronger.*

Minor points
Lines 29-31, "our results suggest that ancient eukaryotes originated within complex microbial ecosystems through a succession of symbiotic interactions that left a footprint of horizontally transferred genes" - the authors have toned down references to symbiosis in their title and abstract, appropriately given the different possible uses of the term 'symbiosis' (as they have pointed out in their response) and the difficulty of interpreting the nature of the relationships between gene donors and recipients in ancestors of eukaryotes. This sentence should also be changed to be consistent with this: "our results suggest that ancient eukaryotes originated within complex microbial ecosystems through a succession of interactions that left a footprint of horizontally transferred genes". Speculation on the nature of the interactions can then be had in the discussion.

Response: *We have re-worded this sentence and dropped the word symbiosis, as the reviewer finds it problematic. We agree that it is difficult to interpret the nature of relationships and with the suggestion of including such speculations in the discussion.*

Lines 40-41, "a host with an Asgard archaeal component", "a host with an Asgard archaeal affiliation" or "a host with an Asgard archaeal ancestry" would be more accurate here

Response: *We have re-worded this sentence as suggested by the reviewer.*

Line 94, “Our reconstructed LECA proteomes comprised an average of 10,255 or 5,995 mLECA-OGs” - the appropriate supplementary materials supporting these figures should be cited here

Response: *Supplementary table 3 supports these figures, and it is cited.*

Line 351-352, “being the detected specific contributions from Alphaproteobacteria (8%), Planctomycetota (8%), and Asgard archaea (4%)” should read “with the detected specific contributions being from Alphaproteobacteria (8%), Planctomycetota (8%), and Asgard archaea (4%)”

Response: *We have corrected this text in the supplementary, as suggested.*

Line 361-362, “The different mechanisms evolved mainly during eukaryogenesis” - we can’t be sure of this, as a significant amount of time elapsed between eukaryogenesis (whatever criteria for this are used) and the emergence of the LECA. Furthermore, the authors’ own conclusions here and in previous work suggest that eukaryogenesis may have been a protracted process that may have been largely complete by the time of the mitochondrial symbiosis. “The different mechanisms evolved mainly in the eukaryotic stem lineage” might be closer to what the authors intended.

Response: *We have rewritten this sentence as suggested by the reviewer.*

Supplementary figure stpf10, a couple of typos: Histidine degadation (should be degradation); Hydroxypropionate-hydroxybutirate cycle (should be Hydroxypropionate-hydroxybutyrate)

Response: *Interestingly, this is a typo that exists in the KEGG database, as we simply transposed the names. We corrected these typos in the figure.*

Referee #3 (Remarks to the Author):

The authors have provided thoughtful, well-reasoned responses to the comments of five reviewers and done a very thorough revision of their manuscript. The improvements are, in my opinion, substantial. There is now more clarity around various key elements of their results and interpretation, including the stem length analysis; this will provide clearer substrate for future discussions and, I suspect, debate. The tweaks to the viral contributions to LECA are well-received (namely addressing the question of directionality); the data say what they say, and I think the authors now present them in a way that is less open to over interpretation.

Response: *We thank the nice comments by reviewer 3 and in particular the appreciation of our efforts during the revision, to accommodate all the suggestions. We agree that the manuscript has improved.*

Having looked at the full set of comments and revised manuscript, I am left wondering about the revised title, and the broader take-home messages from the revised data / results / interpretation. The authors have removed 'symbiotic', which leaves:

"Diverse ancestries reveal complex interactions during eukaryogenesis"

I think this is not sharp enough to be useful.
Diverse ancestries of what? (inferred LECA genes?)
What type of interactions? (syntrophic? Or any and all types of interactions as long as they are 'tight'?)

I would encourage the authors to think of a more explicit description of their results.

The authors feel that I (and perhaps R2) were overly focused on endosymbiosis, which is fair enough. In their rebuttal, they talk about their intended (and more generic) usage of symbiosis, i.e., "tight co-existence". So what does that mean, exactly? Readers will want to know.

And regardless of the intended meaning of 'symbiosis', when it comes to a multiple "acquisition waves" model, the authors are still invoking multiple, sequential 'tight co-existences', between different cells at different time points -- each making their mark in sequence on a proto-eukaryotic cell somewhere between FECA and LECA.

So removing 'symbiosis' from the title does nothing to improve clarity, since the intended meaning is (sort of) still retained in the abstract ("succession of symbiotic interactions...") and elsewhere.

I'd encourage the authors to give the title a rethink. If metabolic interactions is what they feel their results speak to, then may 'syntrophic interactions' is the answer.

Response: *We agree with the reviewer that the current title is perhaps so neutral that it provides little information. We want to note that we are talking here about multiple partners and probably each partner might have had a different type of interaction. Although syntrophy is likely involved in some of the interactions between the prokaryotic partners we cannot discard other possibilities. We thought symbiosis captured the tight coexistence which we think is necessary to explain the amount of transferred genes and was open enough not to suggest a specific type of symbiosis (endo/ecto, syntrophy, predatory, etc). But, as the reviewer noted, we were told to eliminate this word by some reviewers. Given the remaining remarks from one of the reviewers with respect to the word “symbiosis”, we believe that the use of the more specific term “syntrophy” in the title will be problematic for that reviewer and prefer to keep a neutral stand in the title and clearly discuss the findings in the manuscript where ideas can be expressed more clearly. We nevertheless changed the title to specify that with “ancestries” we do refer to “gene ancestries” and changed complex “interactions” to “multiple microbial associations” that conveys the idea that there were multiple microbial partners with strong links (associations), without using words that might be over-interpreted.*

Referee #4 (Remarks to the Author):
I would like to thank the authors for the thorough rebuttal letter, which provided very useful information based on my own comments and those of my colleagues. I am very satisfied with answers to these comments and with the comprehensive experiments that were added to address them. This is reflected by extensive text revisions and additional supplementary texts and figures, and I judge that the manuscript was well improved in the process, gaining depth and clarity. Hence, I have no further comments, and I congratulate the authors on their hard, interesting work that - I think - opens many new perspectives for future studies.

Response: *We appreciate the nice words by reviewer 4 and we are happy that they are satisfied, we have addressed the minor comment and corrected the typo.*

Minor comment/typos
- In the figure Ext. Data Fig. 2 (Line 837) => “Alphaproteobacteria” is misspelled.

Response: *This has been corrected. Thank you for spotting it.*

- Of note however, the Github page link was not working when I attempted to test it: https://github.com/Gabaldonlab/LECAdonors

Response: *Given competing interests in other groups we prefer to keep the repository as private until acceptance of the paper. Unfortunately, github does not allow anonymous shared access to private repositories and this is why we created a folder with the code in the same Zenodo repository for the reviewers to access. This should be accessible anonymously (see response below). The github repository will be made publicly available upon manuscript acceptance.*

- The Zenodo page was available, but with restrictions on its content, which is not practical for review of the file contents.

Response: *We suspect the reviewer may have not used the long version of the link provided. In the Zenodo repository, there is a message about restrictions: “Restricted. The record is publicly accessible, but files are restricted to users with access.” This message affects people without the longer link. The following link: https://zenodo.org/records/13897946?preview=1&token=eyJhbGciOiJIUzUxMiJ9.eyJpZCI6ImMyYWNiMDY4LWEzNTQtNDFlOC1hMTQ4LTNjNjBjNGFhOWRmNSIsImRhdGEiOnt9LCJyYW5kb20iOiJhZDQ5NjQyOTRkMGViZWU3ZjQ0OGYyMTNlMmEwMjIyYiJ9.RTmHhPfEGirPatRDnaJQxMqvqz9xG-ndHU5qzJwricKpmZfRlpZ5hlQd12lnaf7UmOX_vNTO1pL9aEGwOAVdgQ, grants access to all files to any person with this link.*

Referee #5 (Remarks to the Author):
This second version of the manuscript has clearly improved by putting the work better in the context of ongoing work by others in the field, and providing a more critical assessments of results.

Summary of key results
The manuscript reports a reconstruction of the last eukaryotic common ancestor's proteome, and traced back the most likely phylogenetic origin of each protein family. They show

evidence for several waves of horizontal gene transfer from diverse bacterial and viral donors some of which apparently prior to the mitochondrial endosymbiosis.

Originality & significance
This manuscript makes a considerable contribution to understanding the evolutionary origin of eukaryotes. Using the latest genomics data, it provides an updated perspective on eukaryogenesis, reinforcing existing models of serial symbiotic interactions with a diverse array of bacterial, archaeal, and viral taxa. It also offers more precise insights into the relative contributions of prokaryotic donors and their taxonomic affiliations than previous studies.

The methodology appears robust, and the conclusions well-founded.

Response: *We appreciate the nice words by reviewer 5, we have now addressed the remaining concerns.*

References
However, more credit needs to be given to previous work. The concluding statements of the manuscript (L373-388) give the impression that this is the first study to suggest a role for viruses, -and specifically Nucleocytoviricota—, in the transfer of non-eukaryotic genes to LECA. This claim appears inaccurate. Relevant prior studies by other researchers should be cited, and the findings of earlier work should be thoroughly discussed, particularly in terms of the extent of gene transfer proposed and the methodological robustness of the conclusions.

Response: *We agree with the reviewer that the role of viruses has been previously discussed, we now cite more articles in this regard.*

Clarity
Several places in the manuscript lack precision, which makes it difficult to read. E.g.,
- L 79 'alignment profile searches'. The type of the profile and the searches should be briefly specified.

Response: *We apologize, we need to be succinct in the main text due to space constraints. We have added protein profile similarity searches to be more specific, but further details are provided in the materials and methods section.*

- L238-240, to be rephrased.

Response: *This part corresponded to an older version of the figure, we have rephrased this part of the legend.*

- L363: Fig. 1c seems not to depict the assertion preceding the reference to this figure.

Response: *This was a typo, now corrected to Fig 2c, thanks.*

Several grammatical errors were spotted, e.g.,
- L128 'extant genome size' -> proteome size of extant FLUs
- L149 It is indicated that Fig. 2 shows topologies or sistergroups, but it doesn't.

- L160 'impact of ..thresholds "ON" not "in". Does 'trees' mean 'OGs'?
- L207 'trees... were of origin..' rephrase.
- L254 'with' -> 'from'
- L278-280 check
- L284-285 check
- L351 check

Response: *We have corrected all these errors. Thank you for pointing them out.*

Referees' comments:

Referee #1 (Remarks to the Author):
The authors have done a great job to accommodate the comments and suggestions that were brought forward by the reviewers. I think that overall the presented work is convincing, and the discussion about the results is in balance. Eukaryogenesis remains to be a vibrant topic, and I am convinced that the work by Bernabeu et al will, besides providing new insights, also spark new scientific debates. As such, I look forward to seeing this manuscript appear in print.

Response: We are thankful to the very positive comments on our work. We thank all referees for helping us improve our manuscript.

Referee #2 (Remarks to the Author):
I would like to thank the authors for addressing my comments. I am glad that using mixture models helped to improve the robustness of the authors' results, as strengthening the authors' case for their results has been my aim. I acknowledge the significant amount of time and effort that this has involved on the authors' part, and commend them for doing it. As the authors point out, I am the only reviewer to bring up some of these points; certainly other reviewers noticed plenty of points that I missed, that is part of the point of having multiple peer reviewers. My aim with my comments is not to attack the authors, who have done a tremendous amount of work, but to ensure that this work is presented completely and robustly, as it deserves.

Response: We appreciate the constructive criticism and we do think the suggestions by reviewer 2 have ultimately helped us strengthen our conclusions, as rightly pointed out. At no point we felt under attack. We understand there were diverging opinions on what was considered a sufficient level of curation for the purpose of this analysis, but we always felt the discussion was respectful and centered on the arguments and not the persons. We truly appreciate this referee's comments.

* What kind of decontamination, if any, was done on the eukaryotic datasets that were retained for the three eTOLDBs? It looks to me as though the finding of photosystem genes in the LECA (!) in Table ST4 is being driven by contamination from land plants in the ARCVU, LON04 and HET04 datasets (the first two supposedly amoebozoan, the last heterolobosean, but yielding sequences >= 99% similar to plant proteins).

I would like the authors to take the time to look into this more deeply rather than simply discarding the specific orthogroups in question, and to explain briefly how they then ensured that the eTOLDB databases then no longer contaminated, in order to ensure that any other contamination in these and/or other datasets isn't affecting wider conclusions regarding LECA donors. I say this because there are known to be issues with cross-contamination or abundant prokaryotic contamination in some datasets from poorly studied microbial eukaryotes.

Response: When compiling the genomes that comprise the different TOLDBs, we assessed quality by checking genome completeness and the proportion of proteins with low-complexity regions, as indicated in the method Methods. We additionally discarded proteomes with high

bacterial contamination using OMArk. As we trusted the data sources, we did not perform additional decontamination, which we acknowledge could result in some misleading annotations, as pointed out.

To address the reviewer's concern and assess the extent to which contaminant proteins could have driven false positives in the presence of features in our LECA reconstructions, we have performed two additional sanity-check analyses (see Supplementary Methods).

First, we addressed the non-eukaryotic to eukaryote contamination by following the strategy from Williamson et al. (2025), but using a pan-genome approach for a database comprising all proteins of the GTDB reference, eukaryotes in uniprot, eukaryotes in EukProt and a selection of p10K. We additionally assessed potential eukaryotic contamination by using a clustering approach and finding proteins from different species with high levels of sequence identity and alignment coverage, which we considered as potential contaminants. Given that highly conserved proteins could pass these thresholds and that we cannot assess the directionality of the potential contamination, we consider this set of potential contaminants to likely include false positives. Hence, this is a highly conservative approach that allows us to assess the maximum impact of contamination on our analyses.

Having run those filters to our entire dataset, we checked how many mLECA-OGs would have been removed from our final selection. Only 0.09% to 0.22% of the mLECA-OGs were potentially affected, showing a negligible impact of contamination in our final dataset. More interesting was to explore which KO terms were affected by the deletion of these mLECA-OGs in the metaproteome, which we use for the functional reconstruction. Only nine KOs (out of 5370, 0.16%) were flagged as potentially affected by contamination. Importantly, these included two photosystem KOs that raised the concern of the reviewer. However, we also found bona-fide LECA proteins such as the mitochondrial F-type H+-transporting ATPase subunit beta, reinforcing the abovementioned idea that eukaryote-to-eukaryote contamination detection is prone to false positives, preventing us to use it as a general filtering approach.

These analyses compellingly show that our conclusions and reconstructed LECA traits are not significanty affected by potential contaminants in our dataset. We have now included this analysis in the supplementary material, and we have flagged the affected modules and KOs in supplementary table ST4, changing their completeness to the reconstruction without the putative contaminant KOs. We additionally provide the list of mLECA-OGs with any putative contaminants in the zenodo repository (see folder called contamination_analysis).

* I'm finding it difficult to find the source data underlying the donor KOs and modules given in the donors_reconstruction folder (i.e., which sequences give rise to which KO numbers); it doesn't seem to be as simple as cross-referencing donors_reconstruction/donor_genomes.tsv with the donor KOs information found in mLECA_OGs/annotations/ (at least, not with regard to the mitoancestor)

Response: We are sorry that the amount and complexity of the data provided make it a bit difficult to navigate the information. To facilitate this task, we have added a file to the

repository that links donor sequence to its annotation (see file anno2seq.tsv in the donors_reconstruction folder) .

* I very much appreciate that the authors have added a degree of ‘sanity checking’ to the data and more detailed discussion regarding the LECA proteome, including more citations. I appreciate the authors noting in their response that the new filters seem to have modified some of the putative LECA functions that they had previously mentioned. I would like to reiterate that my concern hinged on the fact that there would most likely have been other such examples that I was too unfamiliar with to be able to comment on.

Response: We agree this comment was very helpful and the additional level of curation has likely removed most cases of concern.

A major concern of mine in this section was precisely because it seemed to me that a small but significant proportion of misidentified LECA proteins could point to issues affecting conclusions with regard to the ancestry of the LECA proteome as well. The authors state that “To minimize annotation artefacts we used a taxonomy-informed approach, assessing the taxonomical breadth of proteins annotated as each KO in KEGG and removing KOs that were domain-specific to Archaea or Bacteria, or that were too restricted to a specific eukaryotic group (e.g. mammals) (see Methods), therefore restricting the annotation to KOs that were either unique to eukaryotes but broadly covered within them, or KOs that are widespread among both eukaryotes and prokaryotes”, so I would just like to know whether removing these proteins affects the set of putative LECA proteome donor lineages.

Response: We are sorry for this mistake as this sentence does not clearly reflect our procedure. As it is written the sentence suggests that we removed proteins annotated with non-general KOs. However, we did not remove them, we simply annotated using the subset of general KOs, as mentioned in the suppl. Methods. The mLECA-OGs that did not have a suitable KO from this general set were annotated with the function of the sister group, if available, as this is the best proxy for the function of that LECA family. We now have rephrased this sentence.

As for the remark regarding the LECA proteome donor lineages. In any case, the set of putative LECA proteome donor lineages are inferred from the phylogenetic trees directly, for each TOLDB/criterion independently, irrespective of its functional annotation.

If these aren’t significantly affected, then since the abstract and major messages of the paper highlight LECA proteome ancestry rather than its specific composition, I am going to simply request the following change:

The authors point in the supplementary discussion, “We thus stress that the purpose of this reconstruction is to approximate gene families and functional categories likely present in the ancestral LECA proteome, rather than to provide a carefully curated and detailed picture of LECA’s traits” is an important qualifier, and I would like to see it added to the section on the LECA proteome in the main text. I do not feel that this will invalidate the authors’ hard work on this section, or the main messages of the paper.

Response: We agree, we are happy to accommodate this request and we have included this qualifier in the main text. Thank you.

Minor comments and suggestions

- Regarding another reviewer's comment, the Zenodo link that the authors provided in their response didn't allow me access, but the link provided in the main article file did allow me to download all of the files together (though not to download each file individually).

Response: The Zenodo link will be fully public upon publication and these issues won't exist anymore. We apologize for the inconvenience, as this was the only way to share the data during the review process.

- Discussion of the LECA proteome will necessarily need to be limited (despite the amount of work that the authors clearly put into expanding the discussion!), but I did find it striking that mitochondria weren't mentioned in the LECA proteome discussion section at all. I don't have strong feelings regarding whether these are discussed specifically or not, but I would like to ensure that mitochondrial proteins weren't accidentally discarded as the result of one of the filtering steps, and it would probably be helpful to the reader for the authors to at least mention whether discussion of them was omitted in the interest of space or for another reason.

Response: We agree with the reviewer as mitochondrial pathways are an important core set of LECA, we have these proteins and we now briefly mention them in the supplementary discussion.

- Line 116, "suggesting that LECA was most likely aerobe" should read, "suggesting that LECA was most likely an aerobe"

Response: Thank you for pointing this out, we have corrected this.

- The Table S6 legend states that "We further restricted to those KOs that are pan-eukaryotic according to KEGG", but some entries, despite having a KO MRCA in eukaryotes, mention only animal and fungi KEGG kingdoms, so I'd suggest clarifying in the table legend that the eukaryote MRCA was used as a criterion (presumably).

Response: We have included this in the table legend, as suggested.

- For discussion of gene transfers mediated by members of the Nucleocytoviridicota, I'd suggest also citing Karki et al. 2025 https://doi.org/10.1007/s00239-025-10246-8 .

Response: We have now included this citation.

Referee #3 (Remarks to the Author):

I thank the authors for their thorough second revision and response to reviews. I am satisfied and have no further comments on the manuscript.

Response: We thank the reviewer for the comments.

Referee #4 (Remarks to the Author):

I would like to thank again the authors for the extensive revisions and discussions provided across the rounds of revisions, the manuscript is much improved and present clearly the data and results, while offering a balanced discussion on the limitations of the methodology. I also agree with the new version of the title.

This work constitutes a major contribution to the ongoing debate on eukaryogenesis.

Response: We are very thankful for the positive comments of this reviewer, and the appreciation of our work.

I have no further comments, except for a typo on line 351: « Desulfobacterota » is the correct name for the phylum.

Response: thanks, we have now corrected it.

Referee #2 (Remarks to the Author):

I would like to thank the authors for the extra steps taken to decontaminate their data. I acknowledge that this is a great deal of work, and share the authors' frustration that at this time, biological, methodological and taxon-sampling limitations mean that data from most eukaryotic groups must be treated with caution.

However, the decontamination analyses have been performed as a post hoc step, rather than being incorporated into the results shown in the manuscript or the supplementary files provided: the numbers of mLECA-OGs reported remain identical in this revised version of the main manuscript, as do the contents of supplementary tables 4, 5 and 7, supplementary folders with mLECA-OGs, inferred_metabolisms, and so on. The authors do provide lists of putative contaminated KOs and OGs in the zenodo archive, but the task of subtracting these from the results presented in the rest of the manuscript is left as an exercise to the reader.

Response: We thank the reviewer for their comments, we agree that data should be considered with caution. Regarding potential contamination that we identified in the reviewed manuscript, we want to emphasize that: while it is true that the identified contaminants were flagged but not removed from the proteome, they were not considered in any downstream analyses including the inference of LECA metabolic modules or discussed LECA traits. Moreover they were removed before recomputing all calculations and figures that depend on KOs such as the inference of FLEUKs or donor descendants. As such all the presented results were clean with regards to these potential contaminants and the reader did not have the need to subtract the contaminants from these results, only from the list of mLECA-OGs where they remained. We are sorry that we failed to explain this with more clarity.

Our decision not to re-run (for a third time) all computed trees was based on this minimal impact of contaminants and on grounds of the large computing time (and therefore carbon footprint) involved. Nevertheless we appreciate the concern of the reviewer that this is a post-hoc analysis, therefore we now have performed an even more stringent filtering and have removed the identified proteins before recalculating the phylogenetic trees, the mLECA-OGs and all subsequent analyses. As you will see, this has indeed improved the signal, as expected. This has led to secondary signals such as that of Thermoproteota or Gammaproteobacteria (which we showed in previous versions that they derived from Alphaproteobacterial and Asgard signals) to not pass the thresholds, while the proposed additional contributors (Myxococota, Planctomyctetota and Viruses) are now supported by more stringent thresholds. Despite slight changes in relative percentages in our current reconstructions, the overall trends and discussed results remain the same.

I appreciate that the authors report that **only a small proportion of KOs and OGs are affected**, and understand their position that the **overall conclusions are therefore not changed**. However, showing analyses **known to be based on flawed data** is inappropriate, and it is quite unreasonable to expect the casual reader to **cross-reference each of the reported results with the supplementary materials to infer the true result**, free of contamination (especially when the main text gives no indication that decontamination was done post hoc - on the contrary, "To alleviate potential artefacts arising from phylogenetic reconstruction, including the effects of unsampled lineages, contamination, and recent HGT,

we used state-of-the-art methodologies and compiled curated datasets" would lead anyone reading only the main text to believe that the data were throughly decontaminated from the start).

Response: We are happy to read that the reviewer acknowledges that only a small proportion of KOs and GOs are affected and understands that this does not change the overall conclusions. We disagree in the consideration that the data is "flawed", we have shown that the level of noise is very limited and that this does not affect the conclusions, hence the results cannot be considered invalidated by the existence of some putative imperfections in the data, all analytical measurements have some level of noise or uncertainty and all filtering choices face a delicate balance between false positives and false negatives, as we have discussed in previous revisions.

Having said that, we took the reviewer's concern very seriously and have now re-run phylogenetic computations and all downstream analysis after applying stringent contamination filters. Therefore, we have redone all the analysis, as the reviewer suggests. This implies that no cross-reference or data querying is now necessary from the side of the reader to understand that the sets of mLECA-OGs have no detected contaminants. The contamination cleaning step is explained in the methods after the orthogroups' inference (section 2.2). In summary, we removed 18,256 out of 5,100,325 eukaryotic sequences from eToLDB (0.36%), and it deleted 873 OGs (2.1%), which stopped being LECA due to the species distribution and modified 6.674 (16%) OGs for which we recomputed the trees. The reviewer will be happy to see that this step has indeed improved the signal, while the overall results remain the same. Despite the additional efforts and carbon footprint, we thank the reviewer for the insistence as this additional re-analysis has further strengthened our conclusions.

"However, we also found bona-fide LECA proteins such as the mitochondrial F-type H+-transporting ATPase subunit beta" - actually looking at the data makes it obvious why this was flagged as affected by contamination: OG0001062_3 isn't the mitochondrial F-type H+-transporting ATPase subunit beta, but the chloroplast one, with **plant contaminants from amoebozoan and heterolobosean datasets**. So, "reinforcing the abovementioned idea that eukaryote-to-eukaryote contamination detection is prone to false positives" - not really, if anything in this case it's underestimated the degree of contamination.

Response: We're unsure why the reviewer is talking about OG0001062_3. This is a family that has been correctly flagged as contaminated, but is associated in our files to K02111 ("F-type H+/Na+-transporting ATPase subunit alpha", not F-type H+-transporting ATPase subunit beta) and is associated to photosynthesis. K02133, which is the true F-type H+-transporting ATPase subunit beta, is associated to oxidative phosphorylation pathway and, as seen in the supplementary material, belongs to family OG0011792_1.final in TOLDBB and to OG0014150_1.final in TOLDBC. Therefore, its exclusion is indeed a valid concern, as this protein should be found in LECA.

I picked another tree containing some of the **contaminated heterolobosean data** at random, OG0010678, and found it was **yet another gene of plastid origin with plant contaminants**; this wasn't listed among the OGs affected by contamination. I am not

performing a full analysis of the dataset, so if I'm (again) able to find this so easily through a random check, clearly there are more such cases out there, and the underlying issues have not been fully resolved. The failure to realise this by simply looking at the tree, instead choosing to go with an explanation in line with preconceptions, is to me emblematic of the problems with the paper.

Response: we acknowledged the issue and located the problem, it being that small fragments of genes were not included in the clustering. We have now improved our contamination detection pipeline between eukaryotes by performing a three fold analysis: First we run a homology search of all proteins against all proteins in the three TOLDBs together. We perform a context based analysis where proteins with more than 85% of the hits in the opposite step as the query are considered contaminants. Based on the same analysis we also consider all proteins of different stems with an identity above 95% as contaminants, in this case as we cannot establish directionality both query and target are considered contaminants despite that not being the case. In a final step, we perform a multiple sequence alignment with mafft of all the proteins in an OG and then we calculate pairwise identities between proteins of different stems. Again, those that have an identity above 85% are considered contaminants.

I therefore recommend that the authors carry out a **comprehensive reanalysis of the dataset**, removing as much contamination as possible and **following up on trees with possible indicators of contamination** (e.g. very long branches, topologies suggestive of plastid origins). Without this, it will be difficult to be sure that the data underlying the paper's major conclusions on LECA protein origins are solid.

Response: We appreciate the recommendation, and we have followed it. We consider it important that the reviewer is aware that each re-analysis of the full dataset including the sophisticated models this reviewer asked in a previous iteration, requires over 2 million hours of computation time each time. We were only able to perform this thanks to our privileged access to the Mare Nostrum 5 supercomputer, at the cost of de-prioritizing projects both from our group and others, and by requesting additional computing hours from the Spanish Supercomputing Network (RES). This is the third time we re-run all the analyses. Despite each iteration admittedly increased confidence, the major discussed trends and our major conclusions are based have remained virtually the same. We hope the reviewer acknowledges the efforts and agrees with us that our results are based on consistent trends emerged from robust analyses of the best possible data that we can gather to address the hard question at hand.

Minor comments

Line 114, I'd suggest using alternative phrasing to 'primitive'

Response: we changed the word to "incipient".

Figure 2d, I'd suggest using a darker colour for 'Virus-mediated' as the current dark yellow is hard to read

Response: we have increased the darkness of the Virus-mediated yellow colour, and saturated the Nucleocytoviricota one.

-=-=-=-=-=-=-=-=-=

Comments by R1 on the report from R2

In our view, the reviewer is correct in the statement that contamination is a pertaining issue in eukaryotic data. And indeed**, it would have been best if Gabaldon and co-workers would have made an effort to remove potentially contaminating sequences at the start** of the analyses. The main questions are whether 1) potentially contaminating sequences have a severe effect on the conclusions of the analyses, and 2) whether is is fair to ask from the authors to (yet again) perform a significant amount of work that will lead to the same conclusions.

Response: We agree with them. As they will see, we have now performed the decontamination prior to re-doing all analyses.

Regarding 1:
It is important to realise that **contamination is an important issue that affects all analyses that include environmental sequence data** (MAGs, SAGs, eukaryotic genomes, etc), this is hard to avoid. In our view, Gabaldon and co-workers have **tried to mitigate negative effects of contamination** by **imposing strict requirements** for including eukaryotic KOs and OGs (e.g. by requiring presence in multiple major eukaryotic clades - in effect they have been more strict than the recent analyses by Tobiasson et al). And indeed, in their final analyses, the authors identify only a **relatively small amount of KOs and OGs** that are potentially affected by contaminating sequences. This to us indicates that, even though a certain amount of potential contamination cannot be excluded, the main conclusions of their analyses hold up. **As such we see no ground to be worried.** The fact that the reviewer is able to identify potentially contaminated OGs does not invalidate this notion.

Response: We thank the reviewer for the comment, and we agree with their vision. We tried to mitigate these errors by being strict. Similar recent studies (10.1038/s41586-025-09808-z, 10.1038/s41586-025-09960-6), had either a not exhaustive contamination assessment or none, facing the same scientific problem and with exactly the same limitations.

As we respond to reviewer 2 above, we re-ran our analysis after removing putative contaminants from the OGs. As Reviewer #1 points out, the true impact of contaminating sequences has been anecdotal. Just 2.1% mLECA-OGs were lost initially and 16% of trees needed to be reconstructed. This small share of affected families did not have a significant impact, as we and reviewer 1 anticipated. However, we gained robustness in our annotation, on average, the distribution of KOs across the mLECA-OGs is more homogeneous. We recovered a 99% of homogeneity –percentage of mLECA-OG sequences with the same KO– in the first quartile of its distribution (median 100%, mean 91%), while in the previous run we just recovered 90% in the first quartile (median 100%, mean 90%). Overall, these changes resulted in a more complete metabolic map for LECA (with 2 more modules inferred

in LECA). The impact in the main donors was also gaining robustness of the established donors, which remain the same Alphaproteobacteria, Asgardarchaeota, Myxococcota and Planctomycetota. Their relative contributions to the proteome slightly decreased, but the general trends and conclusions about the functional and features profiling of LECA have not changed. Overall, we believe this additional effort strengthened our dataset and results, while the major trends and conclusions remained stable.

Regarding 2:
Given what is outlined above, **we see no reason why the authors should have to essentially redo their project to come to the same conclusion**. Furthermore, it is important to realise that the **identification of contaminating sequences is not necessarily straightforward**, as it is hard to distinguish these from recent horizontal gene transfers. So even if the authors were make an effort to remove contaminating sequences, the **data will never be perfect**. Finally, the computational resources used to perform this work have been significant, and the results in this project could only be obtained by making certain methodological choices. For as long as the authors are aware of the potential pitfalls of these analyses (and data), and account for these in a reasonable way, **I see no reason why they should start their project from scratch.**

Response: We thank reviewer 1 for their comments. Our decision of not redoing the analysis after the contamination detection in our previous iteration was based on the same arguments. We put in a balance the small anticipated changes, the confidence on the main results after three previous re-analyses each changing different parameter choices, and the significant resources and environmental impact involved in yet another re-analysis. We acknowledge that reviewer 2 weighted this balance differently and we performed a fourth re-analysis. We agree with reviewer 1, as we have discussed in the article and in previous review iterations, any empirical analysis requires data and methodological choices that can be optimal but never ideal. We believe we honestly discuss our choices and limitations in the manuscript. Our major conclusions have survived several changes to these choices, underscoring their robustness, despite the inherent difficulty of discerning ancestral signals. Compared to previously published analysis, our approach can be considered particularly thorough and careful.

General comment: data and code are available in the following Zenodo repository preliminary link: https://zenodo.org/records/13897946?preview=1&token=eyJhbGciOiJIUzUxMiJ9.eyJpZCI6ImMyYWNiMDY4LWEzNTQtNDFlOC1hMTQ4LTNjNjBjNGFhOWRmNSIsImRhdGEiOnt9LCJyYW5kb20iOiJhZDQ5NjQyOTRkMGViZWU3ZjQ0OGYyMTNlMmEwMjIyYiJ9.RTmHhPfEGirPatRDnaJQxMqvqz9xG-ndHU5qzJwricKpmZfRlpZ5hlQd12lnaf7UmOX_vNTO1pL9aEGwOAVdgQ. This link will be substituted by its DOI.

Referees' comments:

Referee #1 (Remarks to the Author):

The authors have yet again have done a substantial analyses to alleviate concerns that were raised by reviewer 2 regarding potential effects of contamination in the data on the overall conclusions of the study. Even though I would like to underscore that in my view it was unnecessary to do yet another re-analyses of the data (the authors had convincingly motivated that the impact of contaminating sequences would not violate their conclusions), I am happy to see that the latest analyses show that 1) the main conclusion that Myxococota, Planctomyctetota and Viruses have been genetic contributors to eukaryotic gene content remains robustly supported, and 2) that ‘secondary signals’ pointing to Gammaproteobacteria and Thermoproteotota (likely derived from Alphaproteobacterial and Asgard archaeal signals) were no longer significantly supported. I hereby conclude that any remaining concerns of Reviewer 1 have been convincingly addressed, and hope to see the revised manuscript published without further delay.

Minor comment:
Line 303: Change ‘Heimdallarchaeota’ into ‘Heimdallarchaeia’ (as is used in the rest of the manuscript).

Response: We thank the reviewer for their comments, we have modified the word.
